# Scaffold-based [Fe]-hydrogenase model: H_2_ activation initiates Fe(0)-hydride extrusion and non-biomimetic hydride transfer†

**DOI:** 10.1039/d0sc03154b

**Published:** 2021-09-10

**Authors:** Spencer A. Kerns, Junhyeok Seo, Vincent M. Lynch, Jason Shearer, Sean T. Goralski, Eileen R. Sullivan, Michael J. Rose

**Affiliations:** Department of Chemistry, The University of Texas at Austin Austin Texas 78712 USA mrose@cm.utexas.edu; Department of Chemistry, Gwangju Institute of Science and Technology Gwangju 61005 Republic of Korea; Department of Chemistry, Trinity University One Trinity Place San Antonio Texas 78212 USA

## Abstract

We report the synthesis and reactivity of a model of [Fe]-hydrogenase derived from an anthracene-based scaffold that includes the endogenous, organometallic acyl(methylene) donor. In comparison to other non-scaffolded acyl-containing complexes, the complex described herein retains molecularly well-defined chemistry upon addition of multiple equivalents of exogenous base. Clean deprotonation of the acyl(methylene) C–H bond with a phenolate base results in the formation of a dimeric motif that contains a new Fe–C(methine) bond resulting from coordination of the deprotonated methylene unit to an adjacent iron center. This effective second carbanion in the ligand framework was demonstrated to drive heterolytic H_2_ activation across the Fe(ii) center. However, this process results in reductive elimination and liberation of the ligand to extrude a lower-valent Fe–carbonyl complex. Through a series of isotopic labelling experiments, structural characterization (XRD, XAS), and spectroscopic characterization (IR, NMR, EXAFS), a mechanistic pathway is presented for H_2_/hydride-induced loss of the organometallic acyl unit (*i.e.* pyCH_2_–C=O → pyCH_3_+C≡O). The known reduced hydride species [HFe(CO)_4_]^−^ and [HFe_3_(CO)_11_]^−^ have been observed as products by ^1^H/^2^H NMR and IR spectroscopies, as well as independent syntheses of PNP[HFe(CO)_4_]. The former species (*i.e.* [HFe(CO)_4_]^−^) is deduced to be the actual hydride transfer agent in the hydride transfer reaction (nominally catalyzed by the title compound) to a biomimetic substrate ([^Tol^Im](BAr^F^) = fluorinated imidazolium as hydride acceptor). This work provides mechanistic insight into the reasons for lack of functional biomimetic behavior (hydride transfer) in acyl(methylene)pyridine based mimics of [Fe]-hydrogenase.

## Abstract 2

We report the synthesis and reactivity of a model of [Fe]-hydrogenase derived from an anthracene-based scaffold that includes the endogenous, organometallic acyl(methylene) donor.

## Graphical abstract

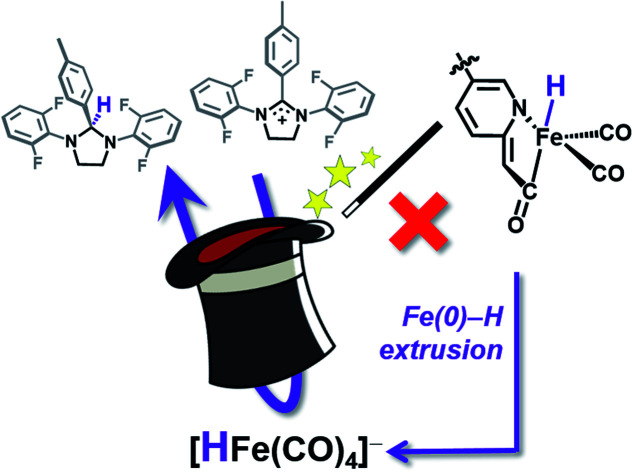

## Introduction

**Scheme 1:**
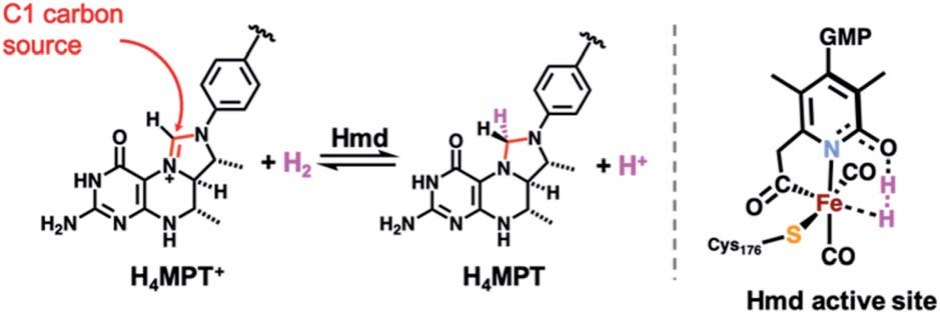
H_2_ activation and hydride transfer reaction catalyzed by Hmd (*left*) and active site's putative key intermediate in H_2_ activation and hydride transfer (*right*).

The search for earth abundant substitutes for precious metal catalysts in energy-related chemical transformations has led researchers to investigate biological precedents that utilize first-row transition metals.^1–6^ Of these enzymes, the [FeFe] and [NiFe] H_2_ases have been studied in detail for their redox active sites for the generation and metabolism of dihydrogen (H_2_).^7–9^ Less studied is the ‘third hydrogenase’ — namely the redox inactive [Fe]-hydrogenase (Hmd). The single iron site in this enzyme heterolytically activates H_2_ and catalyzes hydride transfer to the C_1_ carrier substrate methenyl-tetrahydromethanopterin (H_4_MPT^+^, Scheme 1), thus generating methylene-tetrahydromethanopterin (H_4_MPT).^10^ The refined crystal structure reported by Shima in 2009 identified the active site environment,^11,12^ and a 2019 report^13^ described the crystallized enzyme in both the open (inactive) and closed (active, substrate-bound) conformations. The latter report precisely defined the proximity of the H_4_MPT^+^ hydride transfer substrate to the iron center, and proposed detailed a mechanism of H_2_ activation and hydride transfer using QM/MM calculations^14^ based on the new protein crystal structures.

Since 2009, researchers have significantly advanced structural models of Hmd. However, the scope of functional mimics of Hmd remains limited. Hu and coworkers developed functional systems derived from hybrid molecule|protein systems^15^ and a small molecule system that incorporates an abiotic diphosphine ligand with a pendant amine base.^16^ Our group has reported model systems capable of hydride abstraction^17^ (the enzymatic ‘reverse’ reaction) and hydride transfer^18^ (enzymatic ‘forward’ reaction) with biomimetic substrates. However, both of our reported systems replicated the strong *trans* influence of the Fe–C_acyl_ σ bond in the form of ‘carbamoyl’ ligation (*i.e.* –N^H^C^=O^) as a synthetically more accessible proxy for the endogenous methylene-containing acyl unit (*i.e.* –C^H2^C^=O^); synthesis of the former was originally demonstrated by Pickett.^19,20^ Indeed, the preparation of acyl-containing synthetic systems that rigorously replicate the primary coordination sphere of Hmd *and* exhibit biomimetic reactivity has proven to be a particular challenge due the inherent instability of such compounds and their apparent — and as yet unexplained — sensitivity to base.

In this report, we have more faithfully replicated the Hmd active site in comparison to our previous work by installing the biomimetic methylene linkage. Our synthetic approach uniquely uses an ‘anthracene scaffold’ that provides an accurate and stable means of emulating the biomimetic *fac*-CNS ligation motif. We first describe the synthesis of the model complex and its well-described reactivity in the presence of base. We then demonstrate functional H_2_ activation by a deprotonated iron-acyl model complex that results in liberation of ligand and reduction of the Fe center instead of hydride transfer to a model substrate. Additional base in solution did, in fact, result in successful hydride transfer to the model substrate. However, through a series of control experiments we identify the active hydride transfer agent as the tetracarbonylhydridoferrate species, [HFe(CO)_4_]^−^. Lastly, we describe a mechanistic pathway for reductive conversion of the Fe-acyl unit based on our observations from the structural (XRD, XAS, EXAFS) and spectroscopic (^1^H/^2^H NMR, IR) data collected. These observations provide clear benchmarks and ‘warning signs’ of false positives for other researchers working in the area of biomimetic [Fe]-hydrogenase systems.

## Results and discussion

### Ligand and metal complex syntheses

**Scheme 2:**
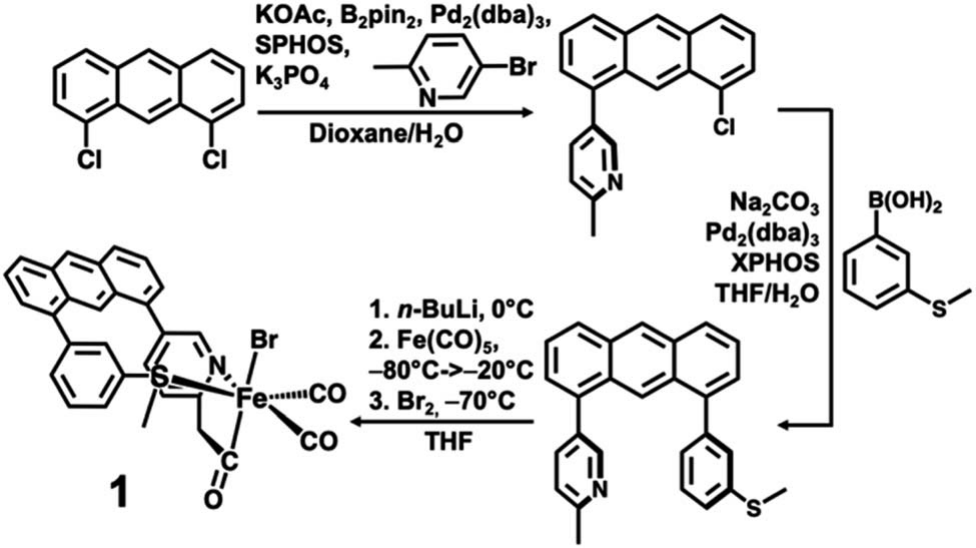
Ligand and metal complex syntheses.

The desired methylpyridine/thioether ligand Anth·C^H3^NS^Me^ (Scheme 2) was synthesized *via* selective mono-coupling of the 2-methylpyridine unit to 1,8-dichloroanthracene, followed by introduction of the aryl-thioether moiety. Briefly, 5-bromo-2-methylpyridine undergoes tandem borylation/Suzuki coupling using B_2_Pin_2_, Pd_2_(dba)_3_/SPhos (∼2 mol%), and weak base (KOAc). The 1,8-dichloroanthracene unit then coupled with the *in situ* prepared boronic acid, affording the asymmetric synthon Anth·C^H3^N·Cl (58% yield, 2.07 g). Subsequent coupling of Anth·C^H3^N·Cl to 3-(methylthio)phenylboronic acid catalyzed by Pd_2_(dba)_3_/XPhos (4 mol%) afforded the target ligand Anth·C^H3^NS^Me^ (Fig. S1†) in good yield (70%, 1.58 g). Similar to reported procedures,^21^ the methylpyridine moiety of the Anth·C^H3^NS^Me^ was lithiated with *n*BuLi in THF at 0 °C, followed by addition of Fe(CO)_5_ (−80 → −20 °C) and Br_2_ (−70 °C) to generate the target complex [(Anth·C^H2^NS^Me^)Fe(CO)_2_(Br)] (**1**) in 77% yield.

The ^1^H NMR spectrum of **1** in *d*^*8*^-THF solution (Fig. S2†) exhibits diamagnetic proton resonances with the characteristic methylene proton resonances observed as diastereotopic doublets at 3.97 and 4.52 ppm consistent with the ligation of the anionic acyl (–C^H2^C^=O^) group to the iron center. The ^13^C NMR under 1 atm ^13^CO (Fig. S3†) revealed the iron-bound carbon of the acyl moiety (*δ* 254 ppm) to be exchangeable (*t*_1/2_ ≈ 3 d), while the ^13^C≡O ligands exchange slightly faster (*t*_1/2_ ≈ 2 d). Facile CO exchange of the acyl moiety was also reported in a complex reported by Hu.^21^

**Fig. 1:**
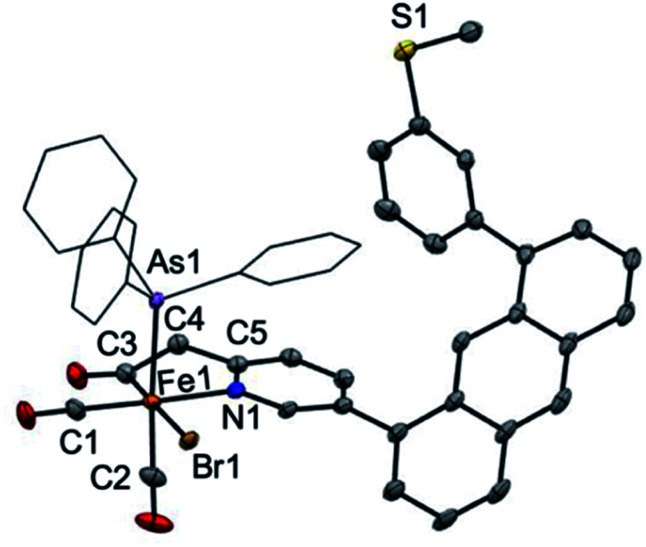
ORTEP representation (30% thermal ellipsoids) for [(Anth·CH^2^NS^off^)Fe(CO)_2_(Br)(AsPh_3_)]. Hydrogen atoms are omitted for clarity and the phenyl groups of AsPh_3_ are depicted as wireframes. Selected bond distances (Å) and bond angles (°): Fe1–C3 = 1.942(3), Fe1–N1 = 2.036(2), Fe1–As1 = 2.4050(5), C3–Fe1–N1 = 83.55(11), C3–Fe1–As1 = 91.76(11), N1–Fe1–As1 = 88.97(6).

Attempts at isolation of single crystals of **1** were unsuccessful. Structural evidence supporting the core motif of **1** was obtained from the derivative complex bound with AsPh_3_. Addition of one equiv. of AsPh_3_ to **1** enabled the isolation of single crystals of the closely related complex [(Anth·C^H2^NS^off^)Fe(CO)_2_(Br)(AsPh_3_)] (Fig. 1). The AsPh_3_ adduct exhibits *fac*-arrangement of the C, N, As donor atoms, with the AsPh_3_ ligand displacing the thioether-S ligand. The orthogonal face is occupied by *cis* carbonyl ligands and the bromide is located *trans* to the acyl–C ligand as proposed in the structure of **1**. Upon coordination of AsPh_3_, a small red-shift is observed in the *ν*(C≡O) stretches to 2024 and 1971 cm^−1^ and a notable blue-shift (∼13 cm^−1^) to 1642 cm^−1^ is observed in *ν*(C=O) stretch of the acyl unit (Fig. S22†). Notably, the bound state of the original thioether-S in **1** was supported by XPS analysis (Fig. S36†).

### Methylene-acyl deprotonation by exogenous base

It is proposed that Hmd utilizes the pendant pyridonate-O as a proton acceptor to facilitate heterolytic cleavage of H_2_. Due to the absence of this basic functionality in the present ligand design, we previously reported^18^ a system in which a bulky phenolate base, NEt_4_[MeOtBu_2_ArO], participated in H_2_ activation to ultimately drive hydride transfer. We thus attempted the analogous H_2_ activation in the presence of this base. However, in a synthetic scale reaction, treatment of **1** in THF with one equiv. NEt_4_[MeOtBu_2_ArO] immediately generated a red-orange solution, accompanied by a precipitate (NEt_4_Br). This contrasts carbamoyl-based systems (NH linkage, not CH_2_), wherein no direct reaction with the same bulky phenolate is observed. Concentration of the filtered solution and successive washes with pentane and Et_2_O removed the protonated phenol byproduct (MeOtBu_2_ArOH), which was identified by ^1^H NMR.

**Fig. 2:**
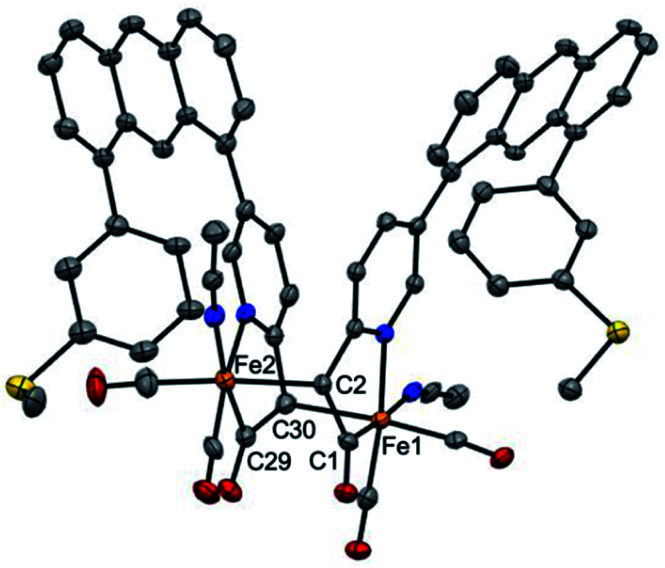
ORTEP diagram (30% thermal ellipsoids) of one of two molecules of **2** in the asymmetric part of the unit cell; hydrogen atoms and solvent molecules are omitted for clarity. Selected bond distances (Å): Fe1–C1 = 1.943(7), Fe1–C30 = 2.186(6), Fe2–C29 = 1.973(7), Fe2–C2 = 2.194(6).

Extraction of the resulting powder into MeCN produced X-ray quality crystals at −20 °C. The resulting structure (Fig. 2) revealed a remarkable result: a *dimeric* complex in which two iron centers bridge *via* the formation of a new Fe–C bond between the deprotonated methine-C (formerly the methylene unit) of adjacent, identical units. The new dimeric species is formulated as [(Anth·C^H^NS^off^)Fe(CO)_2_(MeCN)]_2_ (**2**). The bond distances of the new bridging Fe–C bonds are quite long at 2.186(6) and 2.194(6) Å. These bond distances are significantly longer than the Fe–C_acyl_ bonds at 1.973(7) and 1.943(7) Å.^23^ Notably, the C–C and C–N bond lengths in the pyridine ring of **2** do not significantly deviate from those observed in [(Anth·C^H2^NS^off^)Fe(CO)_2_(Br)(AsPh_3_)] and are thus inconsistent with de-aromatization observed in other methylene-bridged pincer systems upon deprotonation.^22–25^

**Scheme 3:**
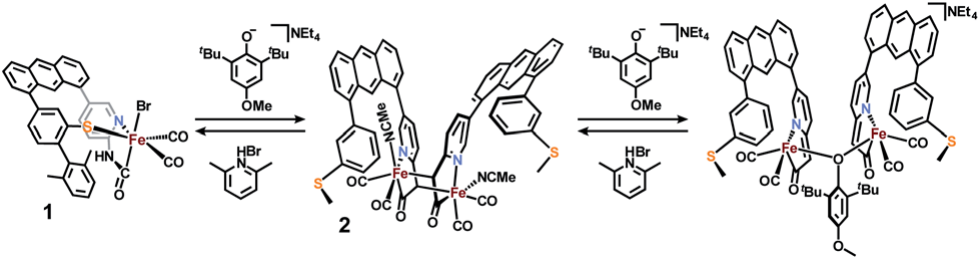
Reversible deprotonation of **1** to form **2**, and proposed bridging coordination of base. Note that the sequence to isolate **2** was performed in MeCN, while the sequence to examine the base-bridged dimer (*far right*) by EXAFS was performed in THF.

**Fig. 3:**
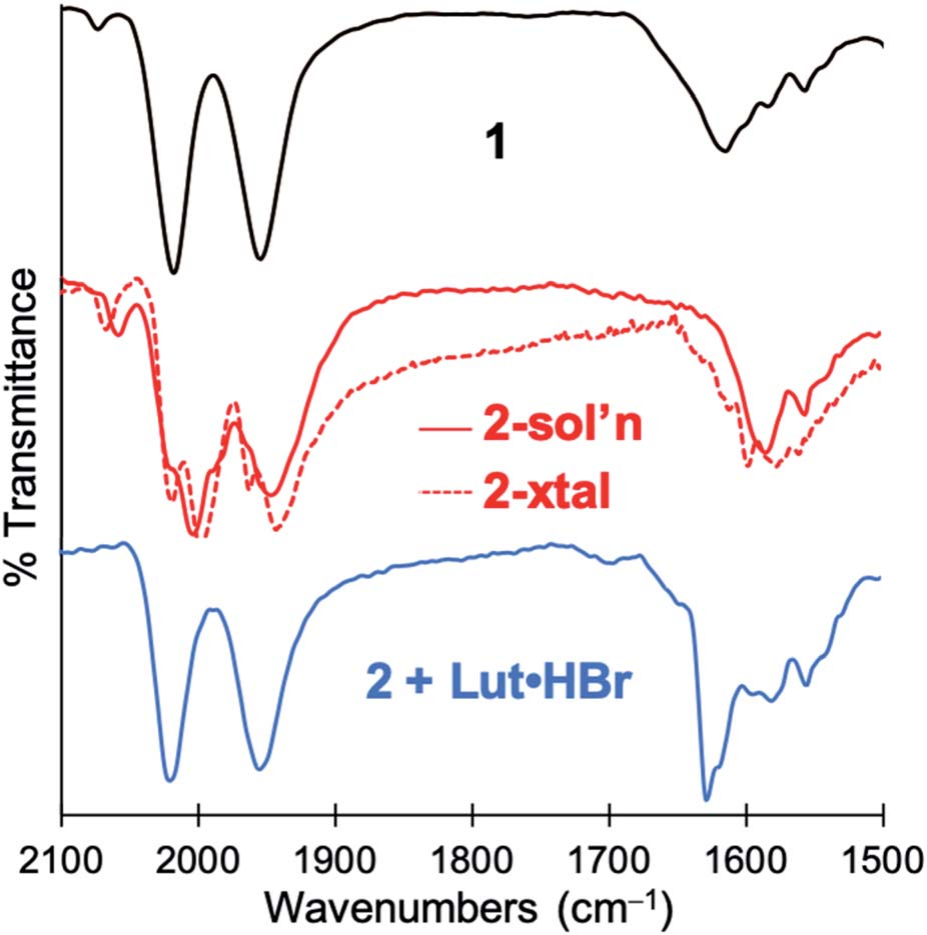
IR spectra demonstrating reversible deprotonation of monomeric **1** (*top*, black line) to form dimeric **2** (*middle*, red line), and protonation of **2** to regenerate **1** (*bottom*, blue line).

Deprotonation of a methylene proton was also evident through shifts in the IR spectrum and changes in the ^1^H NMR spectrum resulting from base addition. The solution *ν*(C≡O) features in the IR spectrum of **1** (2021, 1956 cm^−1^) red-shifted significantly to 2005, 1947 cm^−1^ upon addition of base. The expected four *ν*(C≡O) features for the *C*_2_-symmetric dimer **2** are only observable in the ground crystalline sample at 2021, 1998, 1962, and 1943 cm^−1^ (Fig. S23†). The deprotonation event (Scheme 3) resulting in generation of **2** was also achieved with weaker bases such as NEt_4_[*p*-BrtBu_2_ArO] or NEt_4_[*p*-CNtBu_2_ArO] but not NEt_4_[*p*-NO_2_*t*Bu_2_ArO] — underscoring the surprising acidity of this C–H bond. The deprotonation was clearly reversible upon addition of one equiv. of the weak acid Lut·HBr (2021, 1955 cm^−1^) (Fig. 3). This conversion was also evidenced in the ^1^H NMR spectrum by disappearance of the characteristic diastereotopic methylene proton resonances of **1**, and a new resonance at 4.45 ppm in **2**.

The structure of **2** unequivocally confirms deprotonation of the methylene proton as proposed (but not unambiguously proven) in another acyl-containing model compound (a *mer*-CNS dicarbonyl) recently published by our group,^26^ suggesting that this mechanism is broadly applicable. Furthermore, deprotonation of the methylene-acyl moiety has been observed in another model compound by Song and coworkers through a suggested keto–enol tautomerization and acylation mechanism, although the analogous intermediate was not identified in that case.^27^ These observations suggest that this acyl moiety is rather reactive, and must be fully understood in structural and functional synthetic mimics of this enzyme. Indeed, exogenous base has been noted to decompose previous non-scaffolded acyl-containing model compounds,^16^ perhaps related to this process. The scaffold-supported {Fe(CO)_2_}^2+^ motif of complex **2**, however, is stable and even accommodates further addition of base.

### Bridging coordination of base to the Fe centers (XAS)

Treatment of **1** with *two* equiv. of NEt_4_[MeOtBu_2_ArO] in THF resulted in a more dramatic color change from orange to dark red. Additionally, the IR spectrum of the resulting solution exhibited further red-shifted carbonyl stretching frequencies observed at 1996 and 1923 cm^−1^ (Fig. S24†) in comparison to **1** or **2**. The significant red-shift is consistent with binding of the anionic phenolate donor to displace the Fe–C_methine_ bonds. Coordination of bridging or terminal 2,6-di-*tert*-butylphenolates is not unprecedented in the generation of low-coordinate iron centers.^28,29^ The fully reversible nature of this event was demonstrated by treatment of the dark red solution with two equiv. of 2,6-lutidine·HBr to re-generate a solution of **1** as followed by IR spectroscopy (Fig. S25†).

**Fig. 4:**
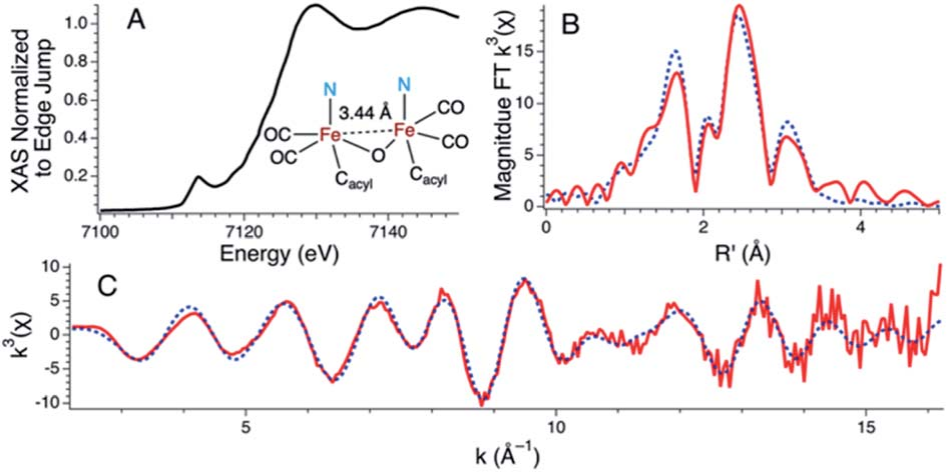
Fe K-edge X-ray absorption data for **1** following treatment with two equiv. of NEt_4_[MeOtBu_2_ArO] base with an inset ChemDraw representation of the proposed primary coordination sphere as modeled by the EXAFS data. (A) The XANES region of the Fe K-edge X-ray absorption spectrum (B) The magnitude Fourier transform of the *k*^3^-weighted EXAFS data depicted with experimental data (red line) overlaid on the best fit model (dotted blue line) (C) *k*^3^-weighted EXAFS data are depicted with experimental data (red line) overlaid on the best fit model (dotted blue line). Reported Model: *E*_o_ = 7131.9 eV; Shell #1 (Fe–CO): N = 2, R = 1.767(2) Å, *σ*^2^ = 0.002(1) Å^2^, Fe–C–O *θ* = 176.8(7)°, Fe⋯O R′ = 2.883(3) Å; Shell #2 (Fe–N) N = 3, R = 2.028(9) Å, *σ*^2^ = 0.004(2) Å^2^; Shell #3 (Fe–Fe) N = 1, R = 3.442(4) Å, *σ*^2^ = 0.002(1) Å^2^; Shell #4 (Fe–C): N = 3.2(4), R = 2.54(1) Å, *σ*^2^ = 0.005(1) Å^2^. *ε*^2^ = 1.51 (over the range of *k* = 2.2–16.2 Å and R = 1.00–3.75 Å).

Attempts to determine the molecular structure resulting from the treatment of **1** with two equiv. of NEt_4_[MeOtBu_2_ArO] (or, equally, treatment of **2** with one equiv. of base) by X-ray crystallography were unsuccessful. The resulting species was thus probed by iron K-edge X-ray absorption spectroscopy (Fig. 4). The XANES region of the iron K-edge X-ray absorption spectrum displays a pronounced pre-edge peak at 7113.5(1) eV corresponding to a nominal Fe(1s → 3d) transition (Fig. 4A); the intensity of this peak is consistent with iron contained in a non-centrosymmetric coordination environment (*e.g.* 5-coordinate distorted square pyramidal).^30^ The EXAFS data for **1** treated with two equiv. of base are best modeled as a dimer of five-coordinate Fe centers ligated by two short CO ligands at 1.77 Å and three additional light atom ligand donors, modeled as N-scatterers, at 2.03 Å, which is similar to the two short carbonyl ligands (1.79 Å) and 3–4 light atom donors, modeled as N-scatterers, at 2.05 Å obtained from the model to the EXAFS data for **2**. It is therefore likely that the three light-atom ligand donors modeled at 2.03 Å in **1** treated with two equiv. of NEt_4_[MeOtBu_2_ArO] — are the acyl-C donor, a pyridine-N donor, and an additional coordinated phenolate-O donor. The Fe–CO bond length observed in **1** with two equiv. of NEt_4_[MeOtBu_2_ArO] is slightly shorter than the average Fe–CO distance observed in **2**, and is consistent with the increased π-backbonding as corroborated by the red-shifted carbonyl stretching frequencies. In addition to the Fe–CO significant multiple scattering pathways found between R′ = 2.5–3.5 Å in the Fourier transform, which dominates the EXAFS of both **1** treated with two equiv. of NEt_4_[MeOtBu_2_ArO] and **2**, an Fe⋯Fe vector could also be located. For **1** treated with two equiv. of NEt_4_[MeOtBu_2_ArO], the Fe⋯Fe vector is found at 3.44 Å; a wavelet transform of the EXAFS data of **2** clearly shows the Fe⋯Fe single scattering pathway is resolvable from the Fe–CO multiple scattering pathways, supporting this assignment (Fig. S42†). In contrast, the XAS data for **2** yields an Fe⋯Fe single scattering pathway at 3.80 Å, which is consistent with the crystallographic results. Taken together, these data are fully consistent with the formulation of **1** with two equiv. of NEt_4_[MeOtBu_2_ArO] as a phenoxyl-bridged Fe⋯Fe dimer (Fig. 4).

### Biomimetic H_2_ activation by the first dimer (**2**)

#### Complex **2** without base

**Fig. 5:**
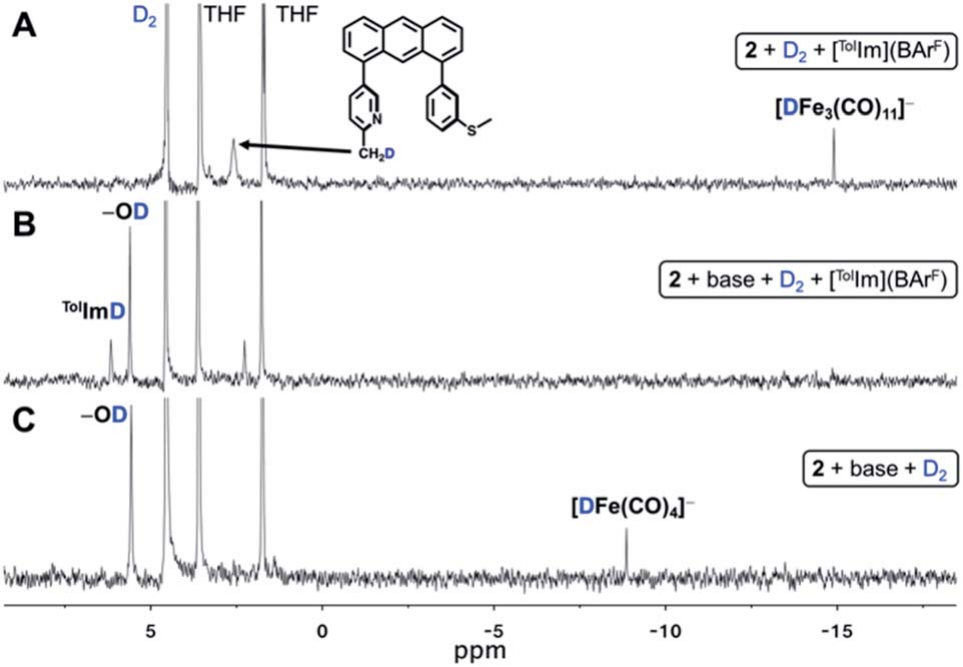
^2^H NMR spectra demonstrating (A) D_2_ activation by **2** in the presence of model substrate [^Tol^Im](BAr^F^). Deuterium labelling is observed at 2.59 and −14.90 ppm, corresponding to the formation of D-labelled free Anth·C^H2D^NS^Me^ ligand and [DFe_3_(CO)_11_]^−^, respectively; (B) D_2_ activation by **2** in the presence of an additional equivalent of base and model substrate [^Tol^Im](BAr^F^). Deuterium labelling is observed at 5.57 and 6.11 ppm, corresponding to the formation of D-labelled MeOtBu_2_ArOD and ^Tol^ImD, respectively; (C) D_2_ activation by **2** in the presence of an additional equivalent of base. Deuterium labelling is observed at 5.56 and −8.87 ppm, corresponding to the formation of D-labelled MeOtBu_2_ArOD and [DFe(CO)_4_]^−^, respectively.

Generation of **2** results in two analogous features of the Hmd active site: (i) a labile coordination site *trans* (MeCN) to the acyl unit and (*ii*) a basic site on the ligand. Notably, in contrast to the endogenous pyridone-O or PNP pincer complexes,^31^ the location of the deprotonated methine-C basic site on the ligand framework *trans* to the open site is not positioned favorably for cooperative H_2_ activation; nevertheless we hypothesized that the deprotonated **2** may still activate H_2_. A crystalline sample of **2** was dissolved in a THF solution containing model substrate [^Tol^Im](BAr^F^) as hydride acceptor and incubated with 7 atm D_2_. The ^2^H NMR spectrum (Fig. 5A) of the reaction was monitored, revealing new resonances at 2.59 ppm and −14.90 ppm, corresponding to deuteration of the 2-methylpyridine moiety of the Anth·C^H3^NS^Me^ ligand and an Fe–*D* species, respectively. No hydride transfer product (^Tol^Im*D*) was observed after three days of monitoring. The isotopic inverse reaction (*d*^*8*^-THF, H_2_) was performed with the free ligand and Fe–*H* species first being observed after 24 hours (Fig. S7†). Incorporation of deuterium into the free ligand indicates that while **2** is competent for D_2_ activation, D_2_ activation and protonation of the methine-C results in the liberation of ligand from the {Fe(CO)_2_} unit. During this process, heterolysis of D_2_ presumably results in the transient generation of the neutral species [(Anth·C^HD^NS^Me^)Fe*D*(CO)_2_]; however, provided only the detection of the liberated Anth·C^H3^NS^Me^ ligand, we were initially unable to unambiguously ascribe the Fe–*H* or *D* resonance at −14.90 ppm.

#### Complex **2** with base

Provided our previous work,^18^ we postulated that an extra equivalent of base in solution would drive H_2_ activation and prevent protonation of the methine-C responsible for ligand loss. Therefore, **1** was first treated with two equiv. of base (*i.e.* NEt_4_[MeOtBu_2_ArO]) and the model substrate [^Tol^Im](BAr^F^). The THF solution was incubated with 7 atm D_2_ and the reaction was monitored by ^2^H NMR spectroscopy. Two new resonances were observed in the ^2^H NMR spectrum at 6.11 ppm and 5.57 ppm (Fig. 5B), corresponding to the *successful hydride transfer product*^Tol^Im*D* and MeOtBu_2_ArO*D*, respectively. Additionally, an unassigned peak at 2.21 ppm was observed that was distinct from the free Anth·C^H3^NS^Me^ ligand resonance. Attempts to optimize the desired hydride transfer reaction and suppress the peak at 2.21 ppm were unsuccessful.

### Competitive formation of reduced Fe-carbonyl species

#### H_2_ activation without substrate (definitive reduced iron extrusion)

To date, spectroscopic observation of a biomimetic Fe–H species capable of hydride transfer to an organic substrate has remained elusive in both Hmd enzyme and synthetic systems. To observe the putative Fe–H intermediate responsible for hydride transfer, we repeated the experiment in the absence of the substrate [^Tol^Im](BAr^F^) with the intention of trapping the reactive intermediate. A THF solution of **1** was first treated with two equiv. of base (*i.e.* NEt_4_[MeOtBu_2_ArO]) and incubated with 7 atm D_2_. Indeed, the ^2^H NMR spectrum exhibited two new resonances at 5.56 ppm and −8.87 ppm, corresponding to MeOtBu_2_ArO*D* and an Fe–*D* species, respectively (Fig. 5C). The isotopic inverse reaction (*i.e. d*^*8*^-THF, H_2_) was carried out and the ^1^H NMR displayed the analogous Fe–*H* resonance at −8.85 ppm within 1 hour of incubation (Fig. S9†). The resulting ^1^H NMR spectrum demonstrated a mixture of products over the course of the reaction, and we therefore attempted to more cleanly generate the Fe–H species through the use of the strong hydride donor, NaHBEt_3_. Again, *in situ* generated **2** treated with 0.9 equiv. of NaHBEt_3_ resulted in a ^1^H NMR spectrum displaying the same Fe–*H* resonance at −8.83 ppm (Fig. S10†).

**Scheme 4:**
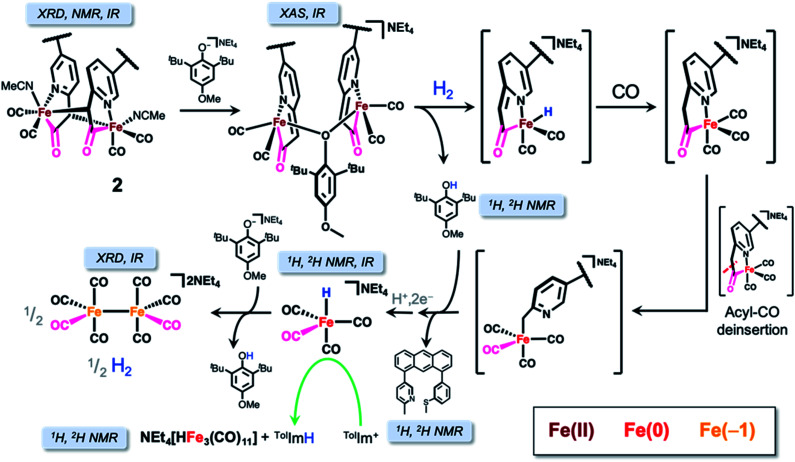
Mechanistic pathway for the reactions of the iron-acyl unit with H_2_ and substrate, with the corroborating structural and spectroscopic evidence as indicated for both the observed and proposed intermediates.

We serendipitously obtained dark red crystals from the THF solution of both the H_2_/D_2_ and NaHBEt_3_ reactions in the NMR reaction tube which were — contrary to our optimistic expectation — identified as the known di-iron carbonyl dianion (NEt_4_)_2_[Fe_2_(CO)_8_] by X-ray diffraction, proving the reduction of the ferrous starting material to Fe(−1). Provided the overwhelming evidence of reductive chemistry and our previous observance of unbound ligand, we considered a conversion pathway to better explain the formation of (NEt_4_)_2_[Fe_2_(CO)_8_] (Scheme 4) in the context of the observed Fe–*H* or *D* resonance and extrusion of the metal center from the anthracene scaffold.

We first contemplated the retrosynthesis of the observed (NEt_4_)_2_[Fe_2_(CO)_8_] product, hypothesizing its derivation from bond formation between two simple {Fe(CO)_4_} building blocks. Upon inspection of known, simple iron tetracarbonyl compounds, we intuited that the product could be derived from initial protonation or deprotonation of one NEt_4_[HFe(CO)_4_] unit, thus providing the necessary 2e^−^ for the reduction of 2Fe^0^ to 2Fe^−1^, concomitant with generation of H_2_ (*i.e.* Fe^0^–H + B → (Fe^2−^ + BH) + Fe^0^–H → 2Fe^1−^ + H_2_ + B). Furthermore, the ^1^H NMR resonance of the Fe–H of NEt_4_[HFe(CO)_4_] was previously reported at −8.8 ppm (*d*^*8*^-THF),^32,33^ which is obviously consistent with the Fe–H resonance (*δ* H/D ≈ −8.8) observed upon H_2_ activation in our studies. To confirm this hypothesis, we independently synthesized PPN[HFe(CO)_4_] (Fig. S11 and S26†) according to literature procedure^32^ and treated it with one equiv. of NEt_4_[MeOtBu_2_ArO] base to deprotonate the Fe–H species. Within minutes of base addition, we observed line broadening in the ^1^H NMR spectrum (Fig. S12†), consistent with reduction to form the intermediate paramagnetic Fe(−1) species concomitant with formation of a red precipitate, confirmed as (NEt_4_)_2_[Fe_2_(CO)_8_] by IR spectroscopy (Fig. S28†). Indeed, PPN[HFe(CO)_4_] is a known reductant^33^ and the control experiment reacting independently synthesized PPN[HFe(CO)_4_] and [^Tol^Im](BAr^F^) (Fig. S13†) proved successful hydride transfer, thus strongly indicating NEt_4_[HFe(CO)_4_] was the active hydride transfer agent in our previous experiments. Furthermore, at longer timepoints in this reaction (days), a new resonance at −14.79 ppm was observed — similar to the previously observed, unassigned Fe–H/D species in Fig. 5A. We now conclusively assign this Fe–H species as NEt_4_[HFe_3_(CO)_11_], a known side-product in hydride transfer reactions of NEt_4_[HFe(CO)_4_].^33^ Indeed, [^Tol^Im](BAr^F^) was separately treated with NEt_4_[HFe_3_(CO)_11_], but no hydride transfer reaction was observed over the course of several days (Fig. S14†), further supporting the role of NEt_4_[HFe(CO)_4_] as the exclusive active hydride transfer agent.

Identification of NEt_4_[HFe(CO)_4_] also confirms the loss of ligand which was observed by ^1^H NMR spectroscopy in both gas reactions utilizing H_2_ (Fig. S15†) and upon treatment with NaHBEt_3_ (Fig. S16†). Furthermore, we re-emphasize the observation of a feature at 2.51 ppm corresponding to deuteration of the methylpyridine moiety of the ligand in the ^2^H NMR spectrum upon generation NEt_4_[DFe(CO)_4_] (Fig. S8†).

The liberation of ligand is predicated upon de-insertion of the acyl unit, which is capable of serving as a CO source in the generation NEt_4_[HFe(CO)_4_]. Upon de-insertion (Scheme 4, right side), the methyl carbanion coordinates to the Fe center to generate an intermediate related to that proposed in the synthesis of the acyl unit by Song and coworkers.^34^ These observations are also consistent with a less electrophilic CO ligand bound to Fe(0) in comparison to Fe(ii) and the demonstrated lability of the acyl unit from labeled ^13^CO exchange experiments.^21^

We investigated the reactivity of the proposed carbanion bound intermediate NEt_4_[(Anth·C^H2^N^off^S^off^)Fe^0^(CO)_4_] by independent synthesis of the lithium methyl-carbanion salt *via* lithiation of Anth·C^H3^NS^Me^ and addition of Fe(CO)_5_ (*i.e.* omitting oxidation by Br_2_ from the synthesis of **1**). The IR spectrum of Li[(Anth·C^H2^N^off^S^off^)Fe^0^(CO)_4_] exhibited CO stretching frequencies of similar energy to the related complex described by Song^34^ and to NEt_4_[HFe(CO)_4_] and did not exhibit an *ν*(C=O) feature above 1600 cm^−1^, as would otherwise indicate acyl ligation (Fig. S29†). We hypothesized heterolysis of H_2_ across the Fe center and bound ligand could explain the generation of NEt_4_[HFe(CO)_4_] and protonation to liberate the free ligand; however, no reaction was observed upon treatment of Li[(Anth·C^H2^N^off^S^off^)Fe^0^(CO)_4_] with D_2_ by ^2^H NMR spectroscopy (Scheme 4, bottom). Instead, treatment of Li[(Anth·C^H2^N^off^S^off^)Fe^0^(CO)_4_] with two equiv. MeOtBu_2_ArOD indicated formation of D-labeled free ligand, Anth·C^H2D^NS^Me^, and NEt_4_[DFe(CO)_4_] by ^2^H NMR spectroscopy (Fig. S17†). Analogous control experiments performed with 2,6-lutidine·HCl provided similar results, supporting that the phenolic proton was the active agent — rather than H-atom or other radical chemistry. As indicated in Scheme 4, the extruded {Fe(CO)_4_} unit undergoes further chemistry to form NEt_4_[HFe(CO)_4_]; however, the nature or mechanism of this particular reaction remains elusive at this time.

Lastly, we considered the initial reduction event of the ferrous starting complex to Fe(0). Based on the activation of H_2_/D_2_ mediated by **2** and the control reaction treating **2** with NaHBEt_3_—and the spectroscopically detected reduced Fe carbonyl species—we postulate that reduction of the ferrous metal center occurs by loss of the unobserved, reactive hydride as a proton along with two electron reduction to form Fe(0). Consistent with our previous work,^18^ detection of the highly reactive (especially anionic) Fe–H species of [Fe]-hydrogenase synthetic models is difficult. Intriguingly, this reductive pathway contrasts the well-characterized intramolecular hydride transfer reaction resulting in methylthiol extrusion observed in another model system from our group (*mer*-CNS; no scaffold),^35^ likely due to the unbound state of the thioether-S^Me^ unit downstream of **1** in this case.

## Conclusions

In summary, we have prepared an acyl-containing anthracene-scaffolded [Fe]-hydrogenase model compound that exhibits a dynamic *fac*-CNS donor motif and performs H_2_ activation. The subtle structural replacement of the previously studied carbamoyl ligation for the methylene-acyl moiety provides a dramatically different reaction pathway to H_2_ activation, which first involves clean and structurally characterized deprotonation of the methylene linker. Notably, the anthracene-scaffolded model complex exhibits well-controlled reactivity upon base treatment in comparison to non-scaffolded systems, possibly due to the controlled hemi-lability of the thioether-S. The methine-ligated dimer **2** resulting from base addition was, itself, competent for H_2_ activation, but hydride transfer to a biomimetic substrate was not observed. Instead, isotopic D-labeled gas experiments revealed formation of free ligand and the reductively extruded hydridoferrate species [HFe(CO)_4_]^−^ (which converts to [HFe_3_(CO)_11_]^−^ over several days). The former species is unambiguously proven to be the active hydride transfer agent in the present study, while the latter species is more stable and thus ineffective for hydride transfer in this system.

Attempts to utilize exogenous base for H_2_ activation in concert with **2** to prevent the loss of ligand and Fe reduction were unsuccessful, but importantly enabled us to structurally and spectroscopically characterize relevant intermediates in this process. Numerous control reactions delineate a mechanistic pathway describing these conversions. This enhanced understanding of this deleterious, competitive process may contribute to the design of a more robust biomimetic reactivity system for understanding the reactivity of acyl(methylene)-containing synthetic analogues of [Fe]-hydrogenase. The inclusion of the authentic and biomimetic pyridone and/or thiolate motifs may drastically alter the reactivity profile(s) described herein, thereby providing more enlightened insight into Nature's delicate choice of donor identity and location in the [Fe]-hydrogenase active site.

## Experimental

### General considerations

Commercially available reagents were used without further purification unless otherwise noted. Suppliers of relevant reagents are described in the ESI.† Solvents used for synthesis were procured from Fisher Scientific and dried over alumina columns using a Pure Process Technology solvent purification system, and stored over 3 Å molecular sieves until use; THF was stored over 3 Å molecular sieves and small pieces of sodium. High-pressure NMR tubes were purchased from Wilmad Labglass (Cat No. 524-PV-7). Infrared spectra were recorded on a Bruker Alpha spectrometer equipped with a diamond ATR crystal, all contained under inert atmosphere. UV/vis spectra were recorded on an Agilent Cary 6000i spectrometer. The ^1^H, ^2^H, and ^13^C were collected using Varian Direct Drive 400 MHz, 500 MHz or 600 MHz instruments. X-ray diffraction and X-ray absorption instrumentation and experimental techniques are described in the ESI.† All cross-coupling reactions and syntheses of metal complexes were performed under N_2_ atmosphere using Schlenk technique or glovebox conditions.

### Ligand syntheses

#### 5-(8-Chloroanthracen-1-yl)-2-methylpyridine (Anth·C^H3^N·Cl)

A mixture of 5-bromo-2-methylpyridine (2.02 g, 11.8 mmol), KOAc (3.43 g, 35.0 mmol), B_2_Pin_2_ (4.43 g, 17.4 mmol), Pd_2_(dba)_3_ (0.213 g, 0.233 mmol), and SPhos (0.194 g, 0.473 mmol) were prepared in 100 mL of dioxane under N_2_ atmosphere inside a glove box. The reaction mixture was refluxed for 6 h, and the resulting orange color solution was used in a next step without isolation. In a separate vessel, 1,8-dichloroanthracene (3.16 g, 12.8 mmol) was prepared in 20 mL of dioxane, and K_3_PO_4_ (7.40 g, 34.9 mmol) was dissolved in 15 mL of degassed water. The anthracene solution and then the K_3_PO_4(aq)_ solution were added into the reaction solution. After refluxing for 12 h, the reaction solution was cooled to room temperature and filtered over Celite pad. The organic products were extracted with ethyl acetate (EA) and dried over Na_2_SO_4_. The product was further purified by silica gel column chromatography (7 : 1 to 4 : 1 hexane/EA) to afford a yellow solid. Yield: 2.07 g (58%). ^1^H NMR (400 MHz, CDCl_3_): *δ* 2.72 (s, 3H), 7.36 (d, *J* = 7.5 Hz, 1H), 7.39 (d, *J* = 8.5 Hz, 1H), 7.45 (d, *J* = 5.8 Hz, 1H), 7.57 (m, 2H), 7.85 (dd, *J* = 7.9, 2.3 Hz, 1H), 7.94 (d, *J* = 8.6 Hz, 1H), 8.06 (d, *J* = 8.5 Hz, 1H), 8.52 (s, 1H), 8.75 (d, *J* = 2.3 Hz, 1H), 8.86 (s, 1H). ^13^C NMR (100 MHz, CDCl_3_): 24.36, 121.73, 122.81, 125.26, 125.54, 125.68, 127.25, 127.35, 127.46, 128.30, 129.17, 130.59, 132.20, 132.28, 132.29, 133.14, 137.05, 137.67, 149.75, 157.67. IR (solid-state): 3036, 1614, 1533, 1307, 1028, 888, 735 cm^−1^. HR-MS (ESI): calcd for [C_20_H_14_ClN + H]^+^ 304.0888; found: 304.0899.

#### 2-Methyl-5-(8-(3-(methylthio)phenyl)anthracen-1-yl)pyridine (Anth·C^H3^NS^Me^)

A mixture of 5-(8-chloroanthracen-1-yl)-2-methylpyridine (Anth·C^H3^N·Cl) (1.75 g, 5.76 mmol), 3-(methylthio)phenylboronic acid (0.967 g, 5.75 mmol), Na_2_CO_3_ (0.610 g, 5.75 mmol), [Pd_2_(dba)_3_] (0.105 g, 0.115 mmol), and XPhos (0.111 g, 0.233 mmol) was prepared in 160 mL of THF : H_2_O (7 : 1) under N_2_ atmosphere. The reaction solution was heated at 85 °C for 12 h under N_2_ atmosphere. After cooling the solution to room temperature, the mixture was quenched with a saturated NH_4_Cl_(aq)_ solution (∼10 mL). The organic product was extracted with DCM and washed with saturated brine (2 × 100 mL). The product was dried over Na_2_SO_4_ and concentrated under vacuum, and further purified by silica gel column chromatography (4 : 1 to 1 : 1 hexane/EA) to afford a yellow solid. Yield: 1.58 g (70%). ^1^H NMR (400 MHz, *d*^*8*^-THF): *δ* 2.45 (s, 3H; thioether-CH_3_), 2.55 (s, 3H; pyridine-CH_3_), 7.26 (s, 1H), 7.28 (s, 2H), 7.34 (m, 1H), 7.41 (m, 3H), 7.53 (t, *J* = 7.6 Hz, 2H), 7.75 (dd, *J* = 8.0, 2.4 Hz, 1H), 8.07 (t, *J* = 7.5 Hz, 2H), 8.55 (s, 1H), 8.60 (s, 1H), 8.61 (s, 1H). ^13^C NMR (100 MHz, *d*^8^-THF): 15.66, 24.51, 122.95, 124.07, 126.23, 126.32, 126.40, 127.36, 127.50, 127.54, 128.11, 128.56, 128.87, 129.16, 129.64, 131.12, 131.20, 133.15, 133.25, 133.98, 138.05, 138.27, 140.19, 141.20, 142.20, 150.64, 158.40. HR-MS (ESI) calcd for [C_27_H_21_NS + H]^+^: 392.1467; found: 392.1479.

### Metal complex syntheses

#### [(Anth·C^H2^NS^Me^)Fe(CO)_2_(Br)] (**1**)

A portion of Anth·C^H3^NS^Me^ ligand (0.20 g, 0.51 mmol) was prepared in 15 mL of THF under N_2_ atmosphere on the Schlenk line. After cooling the solution to 0 °C, 1.6 M *n*-BuLi in hexanes (0.32 mL, 0.51 mmol) was dropwise added into the solution and stirred for 30 minutes. Next, the reaction solution was cooled to −80 °C, and 67 μL (0.50 mmol) of Fe(CO)_5_ (diluted in 5 mL of THF) was injected into the solution over 1 min. The solution was slowly warmed to −20 °C while stirring for 3 h under dark conditions. In a separate flask, 26 μL (0.50 mmol) of Br_2_ was diluted in 5 mL of THF under N_2_ atmosphere. Next, the reaction solution was cooled to −70 °C, and the Br_2_ solution was dropwise added into the reaction solution. After stirring for 2 h at −70 °C, the volatiles were removed under vacuum at room temperature. The residual solid was washed with pentane and Et_2_O to afford an orange-yellow powder. Yield: 240 mg (77%). ^1^H NMR (400 MHz, *d*^*8*^-THF): *δ* 2.46 (s, 3H), 3.97 (d, *J* = 20.6 Hz, 1H), 4.52 (d, *J* = 20.2 Hz, 1H), 7.44 (m, 10H), 8.05 (m, 3H), 8.55 (m, 2H) ppm. IR (solid-state, cm^−1^): *ν*C≡O 2039 (s), 1978 (s), *ν*C=O 1629 (m), *ν*C=N 1584 (m). Anal. calcd for C_30_H_20_BrFeNO_3_S: C 59.04, H 3.30, N 2.30; found: C 58.97, H 3.44, N 2.54.

#### [(Anth·CH^2^NS^off^)Fe(CO)_2_(Br)(AsPh_3_)]

Compound **1** (40 mg, 65 μmol) and AsPh_3_ (20 mg, 65 μmol) were stirred in 5 mL of DCM at room temperature for 2 hours then stored overnight at −20 °C. The solvent was removed *in vacuo*, and the residual solid was extracted with Et_2_O. The Et_2_O soluble fraction was concentrated to afford a yellow-orange solid. Single crystals for X-ray diffraction were grown from vapor diffusion of pentane in to a vial of the complex dissolved in FPh at −20 °C. Yield: 37 mg (62%). ^1^H NMR (400 MHz, *d*^*8*^-THF): *δ* 2.46 (s, 3H), 4.10 (d, 1H), 4.56 (d, 1H), 6.70 (d, 1H), 7.14 (m, 2H), 7.31 (s, 15H), 7.37 (d, 2H), 7.47 (m, 2H), 7.57 (m, 2H), 7.73 (d, 1H), 8.12 (m, 3H), 8.61 (d, 1H), 8.66 (s, 1H). IR (solid-state, cm^−1^): *ν*C≡O 2024, 1971; *ν*C=O 1642. Anal. calcd for C_48_H_36_BrAsFeNO_3_S: C 62.83, H 3.95, N 1.53; found: C 58.24, H 4.08, N 1.08.

#### [(Anth·C^H^NS^off^)Fe(CO)_2_(MeCN)]_2_ (**2**)

Compound **1** (0.050 g, 0.082 mmol) and [(2,6-ditertbutyl-4-methoxyphenolate)(NEt_4_)] (0.030 g, 0.082 mmol) were each separately dissolved in 5 mL THF and mixed. The THF solution of **1** turned red and a white precipitate [(NEt_4_)Br] formed upon mixing. The resultant solution was filtered over Celite and the solvent was removed by vacuum. The deep red residue was washed with pentane and Et_2_O to extract 2,6-ditertbutyl-4-methoxyphenol, affording a red-orange powder. The powder was treated with acetonitrile to give a turbid red-orange solution which was placed at −20 °C producing orange plates suitable for X-ray diffraction. Yield: 54.5 mg (62%). ^1^H NMR (*d*^3^-MeCN, 400 MHz): *δ* 2.51 (s, 3H), 4.45 (s, 1H), 6.90 (d, 1H), 7.13 (s, 2H), 7.35 (d, 2H), 7.45 (m, 4H), 7.58 (m, 1H), 7.67 (s, 1H), 7.86 (d, 1H), 7.92 (d, 1H), 8.10 (d, 1H), 8.50 (s, 1H), 8.59 (s, 1H) ppm. ^13^C NMR (1 : 1 CD_2_Cl_2_, *d*^*3*^-MeCN, 100 MHz): 212.98, 208.67, 172.33, 148.72, 140.92, 139.94, 138.91, 136.71, 136.06, 132.04, 131.85, 129.99, 129.15, 128.12, 127.99, 127.80, 127.27, 126.64, 126.38, 125.71, 125.46, 125.33, 122.92, 115.83, 67.13, 15.45. IR (crystalline solid, cm^−1^): *ν*_C≡O_ 2021 (s), 1998 (s), 1962 (s), 1943 (s) *ν*_C=N_ 1599 (m). Anal. calcd for C_64_H_44_Fe_2_N_4_O_6_S_2_: C 67.38, H 3.89, N 4.91; found: C 67.21, H 4.04, N 4.76.

## Data availability

Crystal structure data has been deposited in the Cambridge Crystal Structure Database, and additional spectra and experimental details are contained in the ESI.†

## Author contributions

Experiments were conceived and designed by J. Seo, SAK and MJR and executed by all co-authors. EXAFS data were acquired and analyzed by J. Shearer. X-ray diffraction data were analysed by VML. ERR and STG performed supporting syntheses and spectroscopic characterizations, respectively.

## Conflicts of interest

There are no conflicts to declare.

## Supplementary Material

SC-012-D0SC03154B-s001

SC-012-D0SC03154B-s002
