# Supplementary material for: Scaffold-based [Fe]-hydrogenase model: H2 activation initiates Fe(0)-hydride extrusion and non-biomimetic hydride transfer
Source: Chem Sci. 2021 Sep 10;12(38):12838–46. doi: 10.1039/d0sc03154b (PMC8494020; doi:10.1039/d0sc03154b)
Supplement: SC-012-D0SC03154B-s001 [file SC-012-D0SC03154B-s001.pdf]

Electronic Supplementary Material (ESI) for Chemical Science.
This journal is © The Royal Society of Chemistry 2021

Supporting Information for:

Scaffold-Based [Fe]-Hydrogenase Model: H_2 Activation Initiates Fe(0)-Hydride Extrusion and Non-Biomimetic Hydride Transfer

Spencer A. Kerns[†*], Junhyeok Seo[‡*], Vincent M. Lynch[†], Jason Shearer[§], Sean T. Goralski[†], Eileen R. Sullivan[†] and Michael J. Rose[†*]

[†]*Department of Chemistry, The University of Texas at Austin, Austin, Texas 78712, United States*

[‡]*Department of Chemistry, Gwangju Institute of Science and Technology, Gwangju 61005, Republic of Korea*

[§]*Department of Chemistry, Trinity University, One Trinity Place, San Antonio, Texas 78212, United States*

[*]*Equal authorship*

Supporting Information Contents **Page**

Section	Page
**Syntheses and Experimental Procedures**	2-14
**NMR Spectra Characterization**	15-20
**^{1}H and ^{2}H NMR of Reactivity Studies**	21-32
**IR Spectra Characterization**	33-42
**UV/vis Spectra**	43-45
**X-ray Photoelectron Spectroscopy**	46-47
**X-Ray Crystallography Experimental Details and Characterization**	48-57
**Iron K-edge X-ray Absorption Spectroscopy**	58-64
**References**	65

Materials and Methods

Reagents, Procedures and Physical Methods. $Fe(CO)_5$, $Pd_2(dba)_3$, $AsPh_3$ were purchased from Strem Chemicals; 2,6-di-*tert*-butyl-4-methoxyphenol, 2,6-lutidine, thallium formate, sodium hydride, H_2 and D_2 from Sigma-Aldrich chemicals; phosphorus pentoxide, hexamethyldisiloxane, *p*-toluic acid, 1,2-dibromoethane, triethylamine K_3PO_4, Br_2, $CDCl_3$ and d^8-THF from Acros Organics; 1,8-dichloroanthroquinone from Alfa Aesar; KOH, KOAc, $NaHCO_3$, NaBr, H_2SO_4, and $HBr_{(aq)}$ from Fisher Scientific; bis(pinacolato)diboron (B_2Pin_2) from Frontier Scientific; Sodium tetrakis(3,5-bis(trifluoromethyl)phenyl)borate, 3-(methylthio)phenylboronic acid, 2-dicyclohexylphosphino-2′,4′,6′-triisopropylbiphenyl (XPhos) from Astatech; 2-dicyclohexylphosphino-2′,6′-dimethoxybiphenyl (SPhos) from Ark Pharm; 5-bromo-2-methylpyridine, 2,6-difluoroaniline, $NaBH_4$ from Oakwood Chemical; NEt_4Cl from TCI; d^3-MeCN from Cambridge Isotope Laboratories, Inc; deuterium oxide from Isotec Inc.

The compounds of $[Fe(CO)_4(Br)_2]$,[1,2] 2,6-difluorophenyl-2-(4-tolyl)imidazolium ($[^{Tol}Im](BAr^F)$) and imidazolidine (^{Tol}Im-H),[3] thallium tetrakis(3,5-bis(trifluoromethyl)phenyl)borate (or $[Tl](BAr^F)$),[4] 1,8-dichloroanthracene[5,6], $[HFe(CO)_4]PPN$[7], $[NDEt_3][DFe_3(CO)_{11}]$[8] were synthesized following the literature procedures. Solvents used for synthesis were procured from Fisher Scientific and dried over alumina columns using a Pure Process Technology solvent purification system, and stored over 3 Å molecular sieves until use; THF was stored over 3 Å molecular sieves and small pieces of sodium. High-pressure NMR tubes (Cat No. 524-PV-7) were purchased from Wilmad Labglass. All cross-coupling reactions and syntheses of metal complexes were performed under N_2 atmosphere as using Schlenk technique or glovebox. Infrared spectra were recorded on a Bruker Alpha spectrometer equipped with a diamond ATR crystal. UV-vis spectra were recorded on an Agilent Cary 6000i spectrometer. The

routine ^{1}H, ^{2}H, and ^{13}C were collected using Varian DirecDrive 400 MHz, 500 MHz or 600 MHz instruments.

Ligand Synthons:

5-(8-chloroanthracen-1-yl)-2-methylpyridine (Anth•^{CH3}N•Cl). A mixture of 5-bromo-2-methylpyridine (2.02 g, 11.8 mmol), KOAc (3.43 g, 35.0 mmol), B_2Pin_2 (4.43 g, 17.4 mmol), $Pd_2(dba)_3$ (0.213 g, 0.233 mmol), and SPhos (0.194 g, 0.473 mmol) were prepared in 100 mL of dioxane under N_2 atmosphere inside a glove box. The reaction mixture was refluxed for 6 h, and the resulting orange color solution was used in a next step without isolation. In a separate vessel, 1,8-dichloroanthracene (3.16 g, 12.8 mmol) was prepared in 20 mL of dioxane, and K_3PO_4 (7.40 g, 34.9 mmol) was dissolved in 15 mL of degassed water. The anthracene solution and then the $K_3PO_{4(aq)}$ solution were added into the reaction solution. After refluxing for 12 h, the reaction solution was cooled to room temperature and filtered over Celite pad. Organic product was extracted with ethyl acetate (EA), and dried over Na_2SO_4. The product was further purified by silica gel column chromatography (7:1 to 4:1 hexane/EA) to afford a yellow solid. Yield: 2.07 g (58%). ^{1}H NMR (400 MHz, $CDCl_3$): δ 2.72 (s, 3H), 7.36 (d, *J* = 7.5 Hz, 1H), 7.39 (d, *J* = 8.5 Hz, 1H), 7.45 (d, *J* = 5.8 Hz, 1H), 7.57 (m, 2H), 7.85 (dd, *J* = 7.9, 2.3 Hz, 1H), 7.94 (d, *J* = 8.6 Hz, 1H), 8.06 (d, *J* = 8.5 Hz, 1H), 8.52 (s, 1H), 8.75 (d, *J* = 2.3 Hz, 1H), 8.86 (s, 1H). ^{13}C NMR (100 MHz, $CDCl_3$): 24.36, 121.73, 122.81, 125.26, 125.54, 125.68, 127.25, 127.35, 127.46, 128.30, 129.17, 130.59, 132.20, 132.28, 132.29, 133.14, 137.05, 137.67, 149.75, 157.67. IR (solid-state): 3036, 1614, 1533, 1307, 1028, 888, 735 cm^{-1}. HR-MS (ESI): calcd. for $[C_{20}H_{14}ClN+H]^+$ 304.0888; found: 304.0899.

2-methyl-5-(8-(3-(methylthio)phenyl)anthracen-1-yl)pyridine (Anth•C^{H3}NS^{Me}). A mixture of 5-(8-chloroanthracen-1-yl)-2-methylpyridine (Anth•^{CH3}N•Cl) (1.75 g, 5.76 mmol), 3-(methylthio)phenylboronic acid (0.967 g, 5.75 mmol), Na_2CO_3 (0.610 g, 5.75 mmol), $[Pd_2(dba)_3]$ (0.105 g, 0.115 mmol), and XPhos (0.111 g, 0.233 mmol) was prepared in 160 mL of THF:H_2O (7:1) under N_2 atmosphere. The reaction solution was heated at 85 °C for 12 h under N_2

atmosphere. After cooling the solution to room temperature, the mixture was quenched with a saturated $NH_4Cl_{(aq)}$ solution (~10 mL). The organic product was extracted with DCM and washed with saturated brine (2 × 100 mL). The product was dried over Na_2SO_4 and concentrated under vacuum, and further purified by silica gel column chromatography (4:1 to 1:1 hexane/EA) to afford a yellow solid. Yield: 1.58 g (70%). ^{1}H NMR (d^8-THF, 400 MHz): δ 2.45 (s, 3H; thioether-CH_3), 2.55 (s, 3H; pyridine-CH_3), 7.26 (s, 1H), 7.28 (s, 2H), 7.34 (m, 1H), 7.41 (m, 3H), 7.53 (t, *J* = 7.6 Hz, 2H), 7.75 (dd, *J* = 8.0, 2.4 Hz, 1H), 8.07 (t, *J* = 7.5 Hz, 2H), 8.55 (s, 1H), 8.60 (s, 1H), 8.61 (s, 1H). ^{13}C NMR (100 MHz, d^8-THF): 15.66, 24.51, 122.95, 124.07, 126.23, 126.32, 126.40, 127.36, 127.50, 127.54, 128.11, 128.56, 128.87, 129.16, 129.64, 131.12, 131.20, 133.15, 133.25, 133.98, 138.05, 138.27, 140.19, 141.20, 142.20, 150.64, 158.40. HR-MS (ESI) calcd. for $[C_{27}H_{21}NS+H]^+$: 392.1467; found: 392.1479.

Synthesis of Metal Complexes

[(Anth•C^{H2}NS^{Me})Fe(CO)$_2$(Br)] (1). A portion of Anth•C^{H3}NS^{Me} ligand (0.20 g, 0.51 mmol) was prepared in 15 mL of THF under N_2 atmosphere in Schlenk line. After cooling the solution to 0 °C, 1.6 M *n*-BuLi in hexanes (0.32 mL, 0.51 mmol) was dropwise added into the solution and stirred for 30 minutes. Next, the reaction solution was cooled to −80 °C, and 67 µL (0.50 mmol) of $Fe(CO)_5$ (diluted in 5 mL of THF) was injected into the solution over 1 min. The solution was slowly warmed to −20 °C while stirring for 3 h under dark conditions. In a separate flask, 26 µL (0.50 mmol) of Br_2 was diluted in 5 mL of THF under N_2 atmosphere. Next, the reaction solution was cooled to −70 °C, and the Br_2 solution was dropwise added into the reaction solution. After stirring for 2 h at −70 °C, the volatiles were removed under vacuum at room temperature. The residual solid was washed with pentane and Et_2O to afford an orange-yellow powder. Yield: 240 mg (77%). ^{1}H NMR (d^8-THF, 400 MHz): δ 2.46 (s, 3H), 3.97 (d, J = 20.6 Hz, 1H), 4.52 (d, J = 20.2 Hz, 1H), 7.44 (m, 10H), 8.05 (m, 3H), 8.55 (m, 2H) ppm. IR (solid-state, cm^{-1}): $\nu_{C\equiv O}$ 2039 (s), 1978 (s), $\nu_{C=O}$ 1629 (m), $\nu_{C=N}$ 1584 (m). Anal. calcd. for $C_{30}H_{20}BrFeNO_3S$: C 59.04, H 3.30, N 2.30; found: C 58.97, H 3.44, N 2.54.

[(Anth•C^{H}NS^{off})Fe(CO)$_2$(MeCN)]$_2$ (2). Compound **1** (0.050 g, 0.082 mmol) and [(2,6-di^{tert}butyl-4-methoxyphenolate)(NEt_4)] (0.030 g, 0.082 mmol) were each separately dissolved in 5 mL THF and mixed. The THF solution of **1** turned red and a white precipitate [(NEt_4)Br] formed upon mixing. The resultant solution was filtered over Celite and the solvent was removed by vacuum. The deep red residue was washed with pentane and Et_2O to extract 2,6-di^{tert}butyl-4-methoxyphenol, affording a red-orange powder. The powder was treated with acetonitrile to give a turbid red-orange solution which was placed at −20°C producing orange plates suitable for X-ray diffraction. Yield: 54.5 mg (62%). ^{1}H NMR (d^3-MeCN, 400 MHz): δ 2.51 (s, 3H), 4.45 (s,

1H), 6.90 (d, 1H), 7.13 (s, 2H), 7.35 (d, 2H), 7.45 (m, 4H), 7.58 (m, 1H), 7.67 (s, 1H), 7.86 (d, 1H), 7.92 (d, 1H), 8.10 (d, 1H), 8.50 (s, 1H), 8.59 (s, 1H) ppm. ^{13}C NMR (1:1 CD_2Cl_2, d^3-MeCN, 100 MHz): 212.98, 208.67, 172.33, 148.72, 140.92, 139.94, 138.91, 136.71, 136.06, 132.04, 131.85, 129.99, 129.15, 128.12, 127.99, 127.80, 127.27, 126.64, 126.38, 125.71, 125.46, 125.33, 122.92, 115.83, 67.13, 15.45. IR (crystalline solid, cm^{-1}.): $\nu_{C\equiv O}$ 2021 (s), 1998 (s), 1962 (s), 1943 (s) $\nu_{C=N}$ 1599 (m). Anal. calcd. for $C_{64}H_{44}Fe_2N_4O_6S_2$: C 67.38, H 3.89, N 4.91; found: C 67.21, H 4.04, N 4.76.

[(Anth•C^{H2}NS^{off})Fe(CO)$_2$(Br)(AsPh$_3$)]. Compound **1** (40 mg, 65 μmol) and $AsPh_3$ (20 mg, 65 μmol) were stirred in 5 mL of DCM at room temperature for 2 hours then stored overnight at −20 °C. The solvent was removed in vacuo, and the residual solid was extracted with Et_2O. The Et_2O soluble fraction was concentrated to afford a yellow-orange solid. Single crystals for X-ray diffraction were grown from vapor diffusion of pentane in to a vial of the complex dissolved in FPh at −20 °C. Yield: 37 mg (62%). ^1H NMR (d^8-THF, 400 MHz): δ 2.46 (s, 3H), 4.10 (d, 1H), 4.56 (d, 1H), 6.70 (d, 1H), 7.14 (m, 2H), 7.31 (s, 15H), 7.37 (d, 2H), 7.47 (m, 2H), 7.57 (m, 2H), 7.73 (d, 1H), 8.12 (m, 3H), 8.61 (d, 1H), 8.66 (s, 1H). IR (solid-state, cm^{-1}): $\nu_{C\equiv O}$ 2024, 1971; $\nu_{C=O}$ 1642. Anal. calcd. for $C_{48}H_{36}BrAsFeNO_3S$: C 62.83, H 3.95, N 1.53; found: C 58.24, H 4.08, N 1.08.

[(Anth•C^{H3}NS^{Me})$_2$Fe$_2$(μ-Br)$_2$(Br)$_2$]. The Anth•C^{H3}NS^{Me} ligand (50 mg, 0.13 mmol) and $Fe(CO)_4Br_2$ (42 mg, 0.13 mmol) were mixed in 4 mL of DCM at –20 °C under N_2 atmosphere inside a glovebox. The solution was stirred at room temperature for ~1 min. Treatment of the solution with ~0.1 mL of THF resulted in the loss of the three $\nu_{C\equiv O}$ features observed at 2107, 2065, 2047 cm^{-1} formed upon initial coordination of the ligand. Volatiles were removed under

vacuum, and the residue was washed with Et_2O (3 × 2 mL). The resultant yellow solid was re-dissolved in DCM, where Et_2O was layered at room temperature to grow yellow block-shape crystals. The Fe(II) dibromide complex in Figure S41 was synthesized solely to use as a control example presenting the Fe-S,N coordination in the XPS analysis.

Other Relevant Syntheses

NEt_4[2,6-Di^{tert}butyl-4-methoxyphenolate]. Under N_2 atmosphere, 2,6-di^{tert}butyl-4-methoxyphenol (0.500 g, 2.12 mmol) was dissolved in dry acetonitrile. NaH (0.051 g, 2.12 mmol) was added to the solution and H_2 was evolved. The solution was stirred for one hour, forming a pale yellow slurry. Next, NEt_4Br (0.445 g, 2.12 mmol) dissolved in acetonitrile was added to the solution and the reaction was stirred for 2 hours. The precipitate (NaBr) was allowed to settle and the solution was filtered through Celite and concentrate in vacuo. The product was washed with pentane and Et_2O to afford an off-white powder, extracted with THF and concentrated in vacuo.

2,6-Lutidine•HBr. Under N_2 atmosphere using Schlenk line, 1.0 g of 2,6-lutidine was prepared in 20 mL of Et_2O in a flask; separately 1.0 g of NaBr, 1.5 mL of $HBr_{(aq)}$, and 0.5 mL of H_2SO_4 were mixed in another flask. The in situ generated HBr gas was transferred into the 2,6-lutidine solution through cannula as precipitating white 2,6-lutidine•HBr in Et_2O. The solvent and unreacted 2,6-lutidine was decanted, and the white solid was dried under vacuum at 60 °C. The residual solid was brought inside a glove box, and washed with Et_2O (3 × 4 mL). After drying under vacuum, the product was stored under N_2 until use.

2,6-Lutidine•HCl

2,6-Lutidine (0.50 mL, 4.3 mmol) was dissolved in 10 mL of Et_2O. The solution was cooled to 0 °C on an ice/water bath, and with stirring, 2 M HCl in Et_2O (3.2 mL, 6.4 mmol) was added

dropwise. The resulting white precipitate was collected by vacuum filtration and washed with 3 x 5 mL of Et_2O. The white solid was dried under vacuum and then stored under N_2 until use.

Reactivity Studies

All reaction solutions were prepared under N_2 atmosphere inside a glovebox prior to the injection of hydrogen gas or ^{13}CO gas.

^{13}CO Gas Exchange. Complex **1** (30 mg, 49 μmol) was dissolved in 0.6 mL of d^8-THF in a J-Y NMR tube. The NMR tube was incubated with 1 atm of ^{13}C labeled CO gas and mixed by inversion using Stuart Rotator SB2. The reaction was monitored periodically by ^{13}C NMR.

Deprotonation of Acyl-methylene C–H, and Reverse Protonation. A small batch of 10 mg (16 μmol) of complex **1** was prepared in 0.6 mL of THF at −30 °C, and the orange solution was treated with 7.0 mg (20 μmol) of 2,6-di-*tert*-butyl-4-methoxy-phenolate (abbreviated as $NEt_4[MeO^tBu_2ArO]$). The reaction instantly generated a turbid red solution, MeO^tBu_2ArOH, and NEt_4Br precipitate. The *in situ* generated, deprotonated species **2** was treated with 3.1 mg (16 μmol) of dry 2,6-lutidine•HBr to re-generate an orange solution, of which IR spectrum confirmed the identity of the species as **1** (Figure 3, main paper). This process was repeated utilizing 2 equiv $NEt_4[MeO^tBu_2ArO]$ (12 mg, 33 μmol), resulting in a dark red solution. The *in situ* prepared solution was subsequently treated 2,6-lutidine•HBr (6 mg, 32 μmol) to re-generate an orange solution of **1**, as evidenced by IR spectroscopy.

D_2/H_2 Activation and Hydride Transfer Studies

Reactivity of Compound 2 with D_2/H_2. Complex **2** (0.010 g, 8.7 μmol) was prepared in 0.4 mL of THF (or d^8-THF) at −20 °C, and the orange solution was treated with 0.3 mL of THF (or d^8-THF) containing [^{Tol}Im](BAr^F) (0.036 g, 32 μmol) was added. The solution was transferred to a high-pressure NMR tube. Following injection of 100 psi (~7 atm) of D_2/H_2 gas into the NMR tube, the reaction solution was gently mixed by periodic inversion using Stuart Rotator SB2. The first signs of H_2 activation (formation of $[HFe_3(CO)_{11}]^-$ and free ligand) were observed in the ^{1}H NMR after mixing overnight. Deuterium labeling of these sights were also observed. After 72 h, no further new resonances were observed by ^{2}H NMR spectroscopy (Figure 5A, main paper).

Hydride Transfer. Complex **1** (0.010 g, 16 μmol) was prepared in 0.4 mL of THF at −20 °C, and the orange solution was treated with 0.3 mL of THF containing NEt$_4$[MeO^{t}Bu$_2$ArO] (0.012 g, 33 μmol). The solution was filtered through Celite in to a vial containing [^{Tol}Im](BAr^F) (0.042 g, 35.6 μmol). The solution was transferred to a high-pressure NMR tube. Following injection of 100 psi (~7 atm) of D_2 gas into the NMR tube, the reaction solution was gently mixed by periodic inversion using Stuart Rotator SB2. After 48 h, three new resonances were observed by ^{2}H NMR spectroscopy at 6.14, 5.56, and 2.21 ppm corresponding to ^{Tol}ImD, MeO^{t}Bu$_2$ArOD, and an unassigned peak at 2.21 ppm, respectively (Figure 4B, main paper). The unassigned peak at 2.21 ppm may correspond to deuteration of the methyl group of the 4-tolyl substituent of the substrate, which is observed at 2.25 ppm in the ^{1}H NMR spectrum. The mechanism of this process though is presently not well understood.

Control Experiments and Complex Conversion Studies

D_2/H_2 activation and generation of [$HFe(CO)_4$]$^-$. Complex **1** (0.010 g, 16 μmol) was prepared in 0.4 mL of THF (or *d*^{8}-THF) at −20 °C, and the orange solution was treated with 0.3 mL of THF (or *d*^{8}-THF) containing NEt_4[MeO^tBu_2ArO] (0.012 g, 33 μmol). The solution was transferred to a high-pressure NMR tube. Following injection of 100 psi (~7 atm) of D_2 or H_2 gas into the NMR tube, the reaction solution was gently mixed by periodic inversion using Stuart Rotator SB2. After 48 h, two new resonances were observed by ^{1}H NMR spectroscopy at 5.56 ppm and −8.87 ppm (Figure S11). These resonance at −8.87 ppm corresponding to [$HFe(CO)_4$]$^-$ was first observed within one hour of mixing. In the ^{2}H NMR spectrum, resonances were observed at 5.34, 2.51, −8.87 ppm (Figure S10).

Generation of [$HFe(CO)_4$]$^-$ species with $NaHBEt_3$. Complex **1** (0.010 g, 16 μmol) was prepared in 0.4 mL of *d*^{8}-THF at −20 °C, and the orange solution was treated with 0.3 mL of *d*^{8}-THF containing NEt_4[MeO^tBu_2ArO] (0.006 g, 16 μmol) to generate deprotonated **2** *in situ*. Next, $NaHBEt_3$ (14 μL, 14 μmol, 1.0 M in THF) was added and the reaction was monitored by ^{1}H NMR spectroscopy for generation of the Fe-H species at -8.8 ppm (Figure S12).

[$HFe(CO)_4$]PPN and [^{Tol}Im](BAr^F). [$HFe(CO)_4$]PPN (0.009 g, 13 μmol) and [^{Tol}Im](BAr^F) (0.032 g, 26 μmol) were dissolved in 0.75 mL *d*^{8}-THF and the solution was transferred to a J-Y NMR tube. The reaction solution was gently mixed by periodic inversion using Stuart Rotator SB2. The hydride transfer product ^{Tol}ImH was observed by NMR spectroscopy at 6.14 ppm after overnight mixing. After days of mixing, a new resonance at -14.9 pppm was observed, corresponding to [$HFe_3(CO)_{11}$]$^-$ (Figure S15).

$(NDEt_3)[DFe_3(CO)_{11}]$ and $[^{tol}Im](BArF)$

$(NDEt_3)[DFe_3(CO)_{11}]$ (8.6 mg, 30 μmol) and $[^{tol}Im](BArF)$ (25.0 mg, 147 μmol) were dissolved in 0.7 mL of THF and the solution was transferred to a J-Y NMR tube. The reaction solution was gently mixed by periodic inversion using Stuart Rotator SB2. ^2H NMR spectroscopy showed resonances at -14.9 ppm corresponding to $[DFe_3(CO)_{11}]^-$. After three days of mixing, no new resonances had appeared in the ^2H NMR, indicating that hydride transfer did not occur (Figure S16).

Formation of $[Fe_2(CO)_8]2X$ from $[HFe(CO)_4]PPN$. $NEt_4[MeO^tBu_2ArO]$ (0.005 g, 13 μmol) was added to a d^8-THF solution containing $[HFe(CO)_4]PPN$ (0.009 g, 13 μmol) and $[^{Tol}Im](BAr^F)$ (0.016 g, 13 μmol). The formation of $[Fe_2(CO)_8]2X$ was observed within 10 minutes evidenced by precipitation of a dark red solid. The dark red solid was confirmed to be $[Fe_2(CO)_8]2X$ by IR spectroscopy (Figure S30).

$[(Anth•C^{H2}NS^{Me})Fe(CO)_4]Li$ conversion to $[HFe(CO)_4]NEt_4$. A portion of $Anth•C^{H3}NS^{Me}$ ligand (0.060 g, 0.15 mmol) was prepared in 5 mL of THF under N_2 atmosphere in Schlenk line. After cooling the solution to 0 °C, 1.6 M *n*-BuLi in hexanes (96 μL, 0.15 mmol) was added dropwise into the solution and stirred for 30 minutes. Next, the reaction solution was cooled to −80 °C, and $Fe(CO)_5$ (20 μL, 0.15 mmol) diluted in 3 mL of THF was slowly added to the solution and the solution was slowly warmed to −20 °C while stirring for 3 h under dark conditions. The solvent was removed and the product (Figure S31) was returned to the glovebox and washed with pentane. A portion (0.010 g) of the product was dissolved 0.7 mL THF and incubated with 7 atm D_2 gas in a high-pressure NMR tube. The reaction was monitored for two days by ^2H NMR spectroscopy, but did not alone indicate D_2 activation. Addition of MeO^tBu_2ArOD (0.008 g, 35

μmol) to the solution resulted in new resonances at 2.50 and -8.84 ppm in the ^{2}H NMR spectrum, indicating free ligand and $[HFe(CO)_4]Li$ formation, respectively (Figure S19) .

Monitoring the reaction of [(Anth•C^{H2}NS^{Me})Fe(CO)$_4$]Li and MeO^{t}Bu$_2$ArOD by UV-vis

[(Anth•C^{H2}NS^{Me})Fe(CO)$_4$]Li (10.0 mg, 16.8 μmol, preparation detailed earlier) and MeO^{t}Bu$_2$ArOD (4.0 mg, 16.8 μmol) were dissolved in 0.7 mL of d^8-THF. A small aliquot of the reaction mixture was taken and dissolved in 1 mL of THF in a quartz cuvette to be monitored by UV-vis spectroscopy, and the rest was transferred to a J-Y NMR tube. The reaction solution in the J-Y NMR tube was gently mixed by periodic inversion using Stuart Rotator SB2. UV-vis spectroscopy showed the disappearance of an absorbance at approximately 450 nm over the course of 20 hrs (Figure S36). After three days of stirring in the J-Y tube, ^{2}H NMR spectroscopy showed new peaks at 2.50 and -8.84 ppm indicating the formation of $[HFe(CO)_4]^-$ and free ligand.

Reaction of [(Anth•C^{H2}NS^{Me})Fe(CO)$_4$]Li and 2,6-Lutidine•HCl

[(Anth•C^{H2}NS^{Me})Fe(CO)$_4$]Li (20.0 mg, 35.4 μmol, preparation detailed earlier) and 2,6-lutidine• HCl (4.0 mg, 35.5 μmol) were dissolved in 0.7 mL of d^8-THF. A small aliquot of the reaction mixture was taken and dissolved in 1 mL of THF in a quartz cuvette to be monitored by UV-vis spectroscopy, and the rest was transferred to a J-Y NMR tube. The reaction solution in the J-Y NMR tube was gently mixed by periodic inversion using Stuart Rotator SB2. UV-vis spectroscopy showed the disappearance of an absorbance at approximately 450 nm over the course of 24 hrs (Figure S37). After three days of stirring in the J-Y tube, ^{1}H NMR spectroscopy showed new peak at -8.84 ppm indicating the formation of $[HFe(CO)_4]^-$ and free ligand.

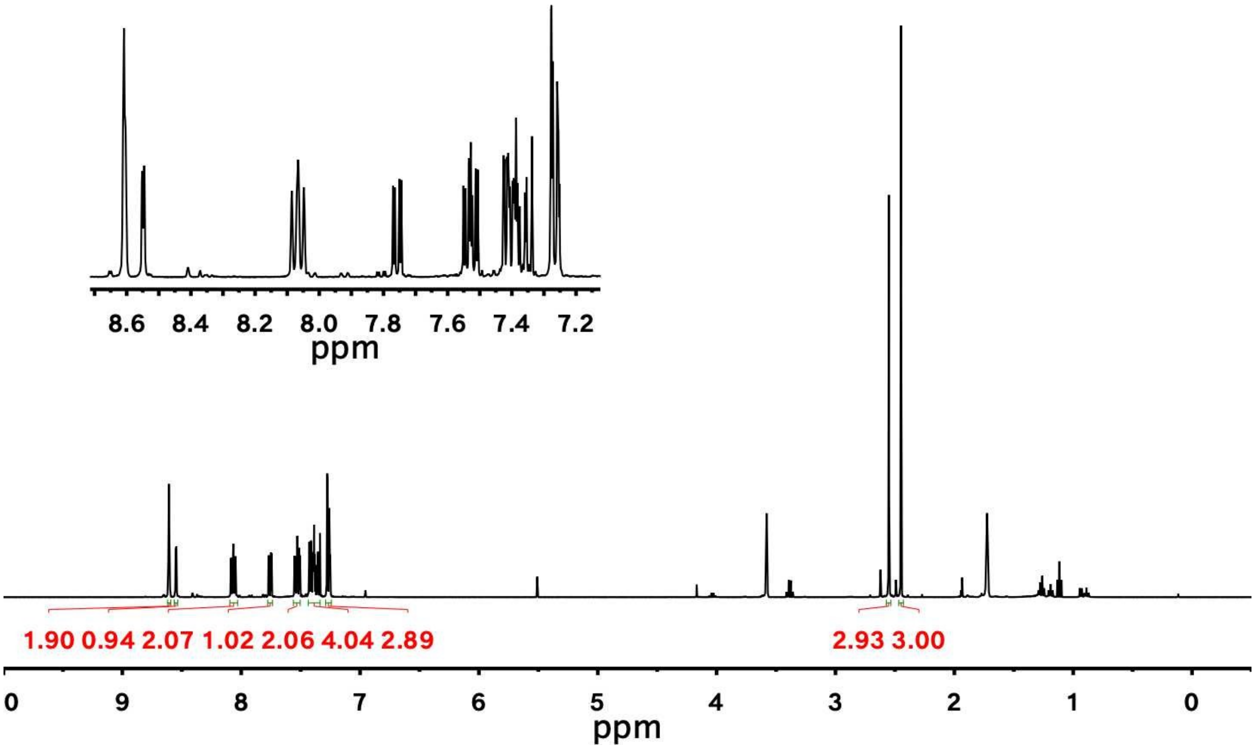

Figure S1. ^{1}H NMR (400 MHz) spectrum of **Anth•C$^{\mathbf{H3}}$NS$^{\mathbf{Me}}$** in d^{8}-THF.

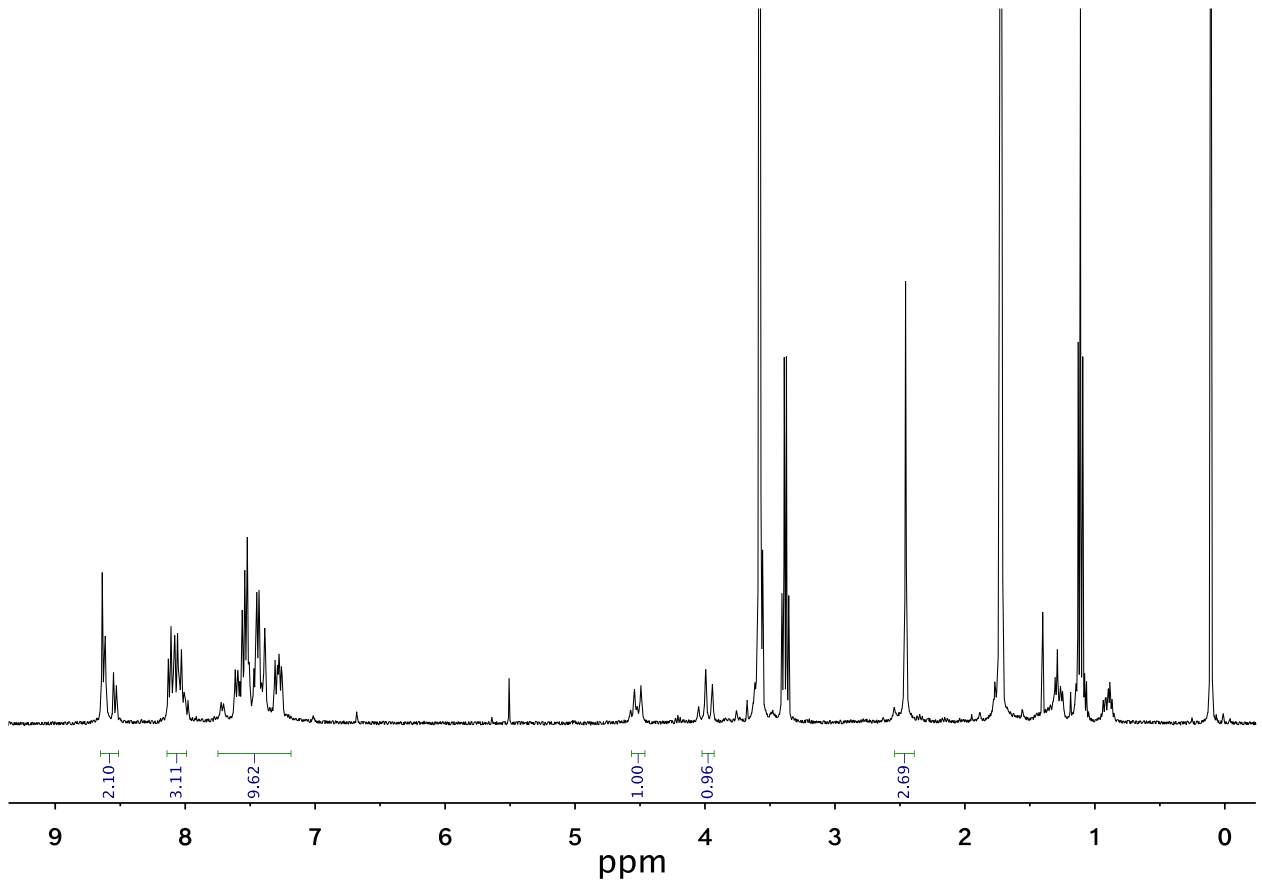

Figure S2. ^{1}H NMR (400 MHz) spectrum of **[(Anth•C^{H2}NS^{Me})Fe(CO)$_2$(Br)] (1)** in d^8-THF. Solvent impurities include DCM (5.53 ppm), Et_2O (1.12, 3.39 ppm), pentane (1.29, 0.89 ppm), and silicon grease (0.11 ppm).

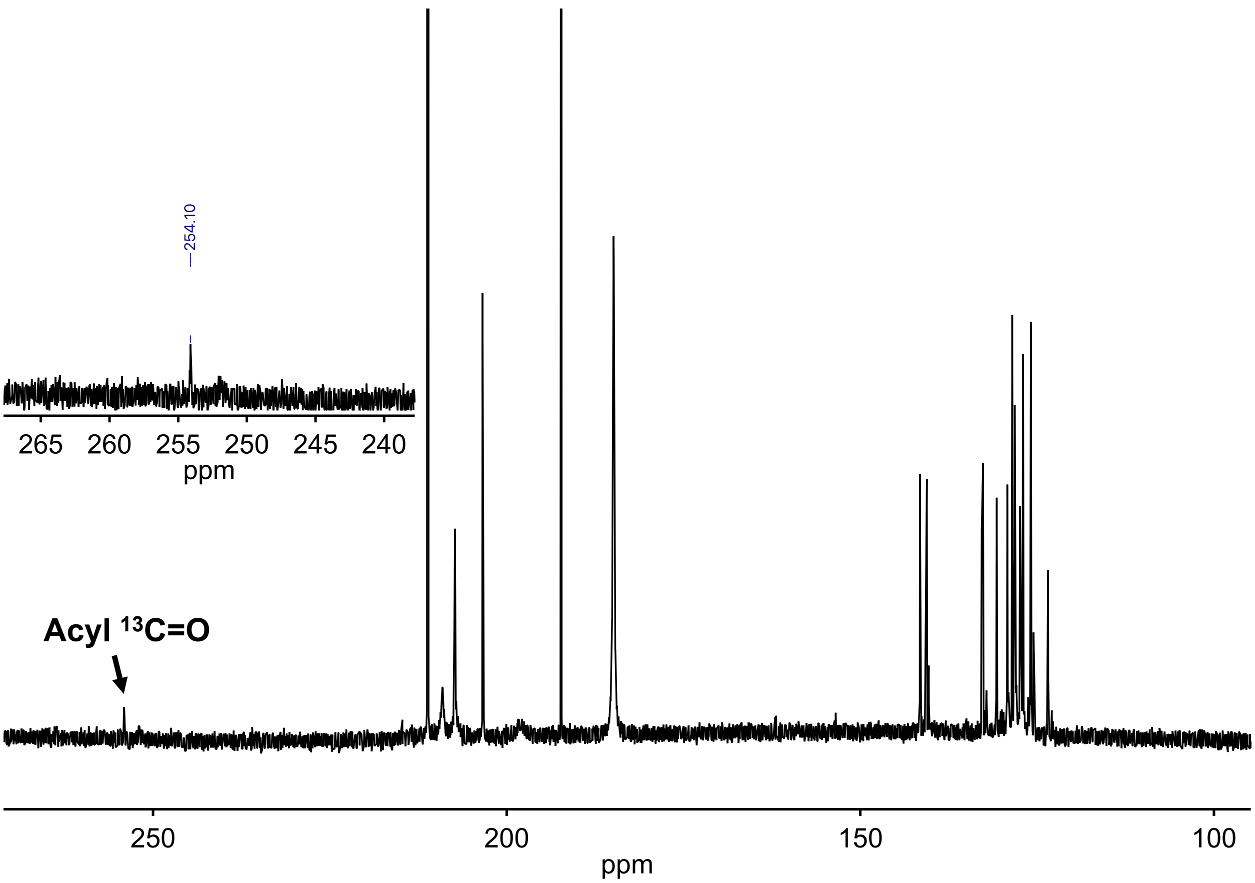

Figure S3. ^{13}C spectrum (100 MHz) of the reaction of **1** with ^{13}CO gas (1 atm) in THF.

Exchange of the acyl CO moiety is observed at 254.10 ppm.

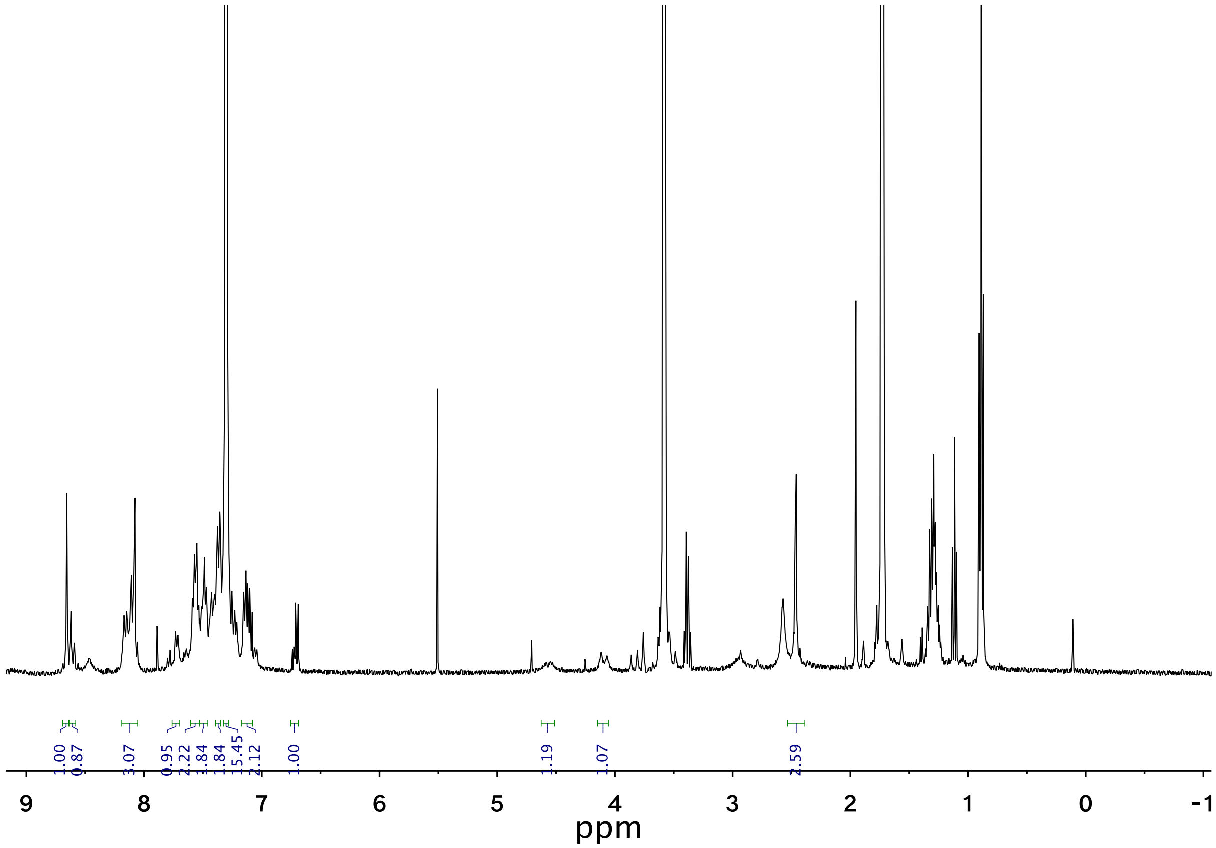

Figure S4. ^{1}H NMR (400 MHz) spectrum of **[(Anth•C^{H2}NS^{off})Fe(CO)$_2$(AsPh$_3$)] (2)** in d^8-THF. Solvent impurities include DCM (5.45 ppm), Et$_2$O (1.12, 3.42 ppm), pentane (0.89, 1.29 ppm).

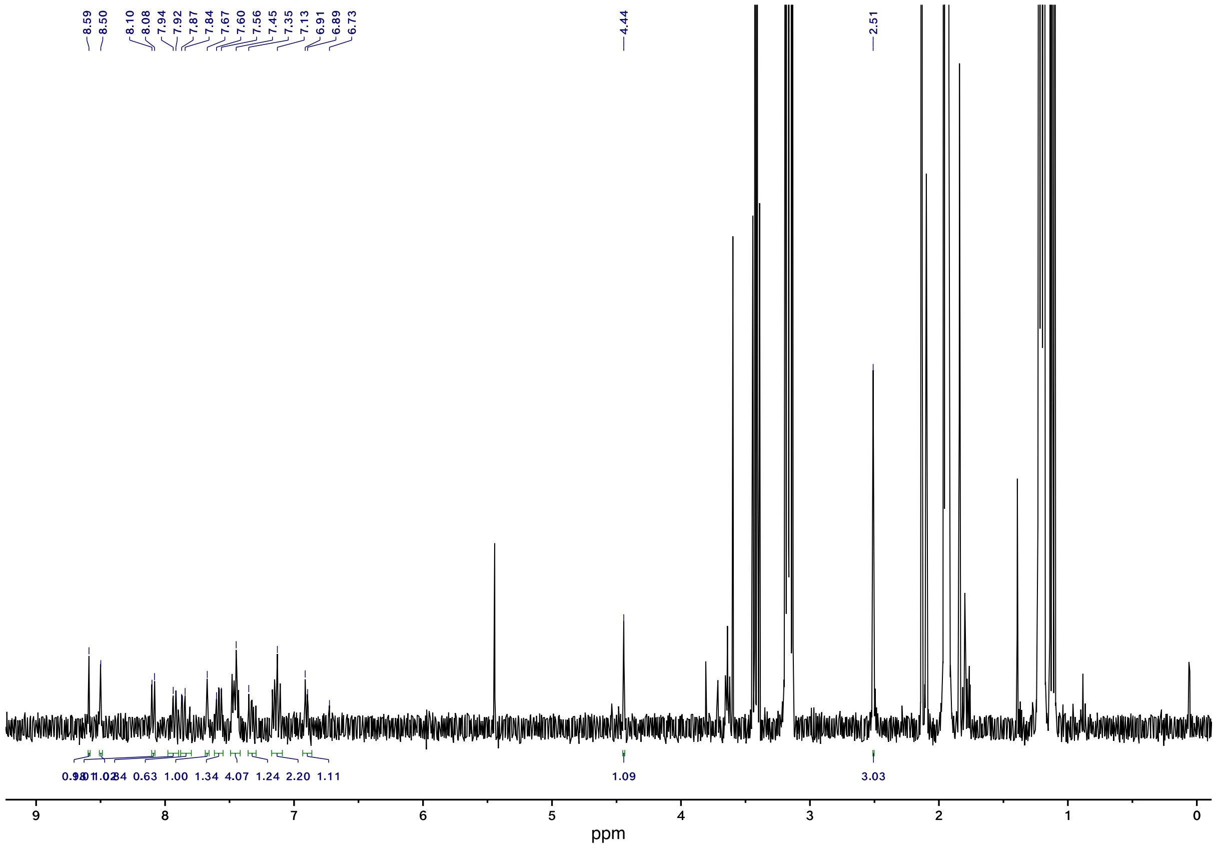

Figure S5. ^{1}H NMR (400 MHz) spectrum of **[(Anth•C^{H}NS^{off})Fe(CO)$_2$(MeCN)]$_2$ (2)** in 1:1 CD_3CN/CD_2Cl_2. Solvent impurities include DCM (5.45 ppm), Et_2O (1.12, 3.42 ppm), 1,4-dioxane (3.60 ppm), water (2.13 ppm), tetrahydrofuran (1.84, 3.60 ppm), pentane (0.89, 1.29 ppm), and NEt_4Br (1.21, 3.17 ppm).

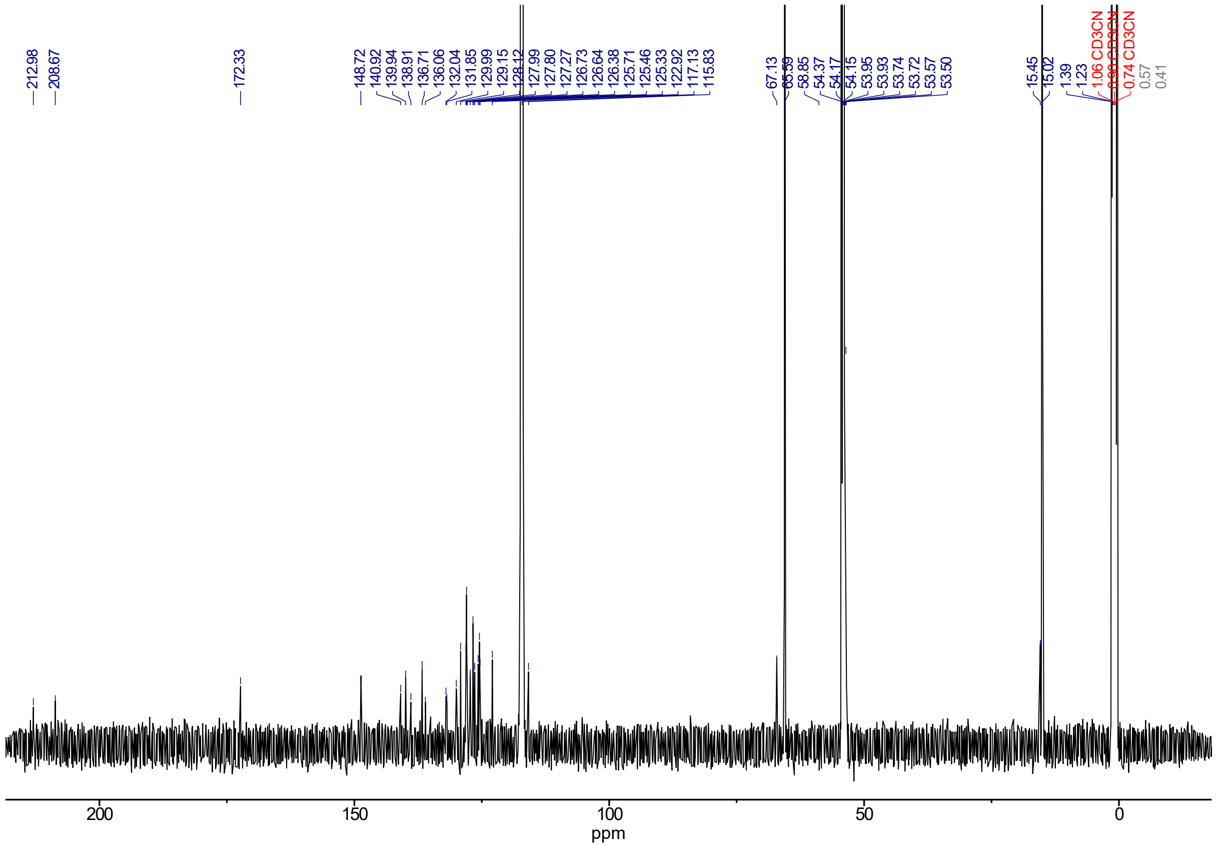

Figure S6. ^{13}C NMR (100 MHz) spectrum of **$[(Anth{\bullet}C^{H}NS^{off})Fe(CO)_2(MeCN)]_2$ (2)** in 1:1 CD_3CN/CD_2Cl_2. Resonances at 65.59 and 15.02 ppm correspond to excess NEt4Br.

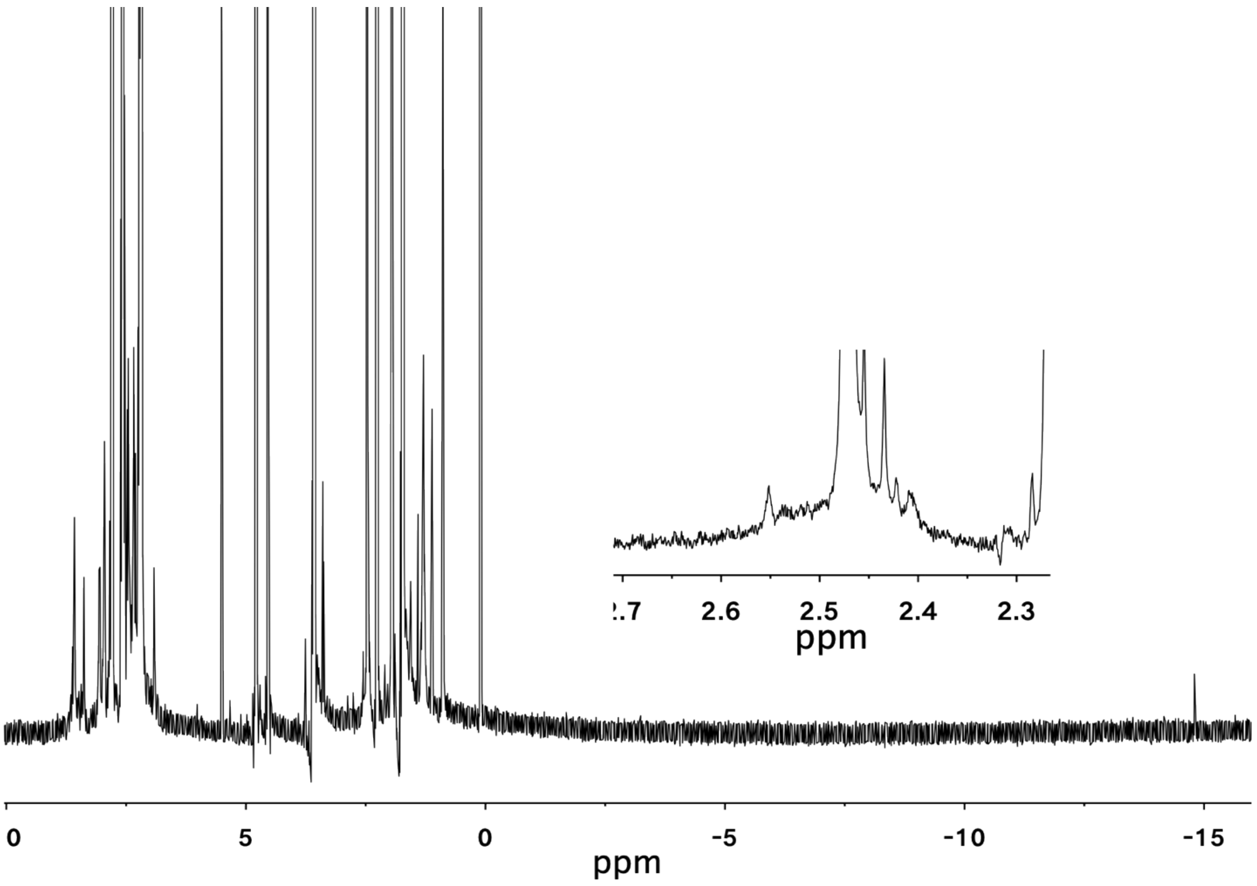

Figure S7. ^{1}H NMR (400 MHz) spectrum in d^8-THF of the reaction between **2**, H_2, and [^{Tol}Im](BAr^F) demonstrating that free ligand at 2.55 ppm and X[$HFe_3(CO)_{11}$] formation.

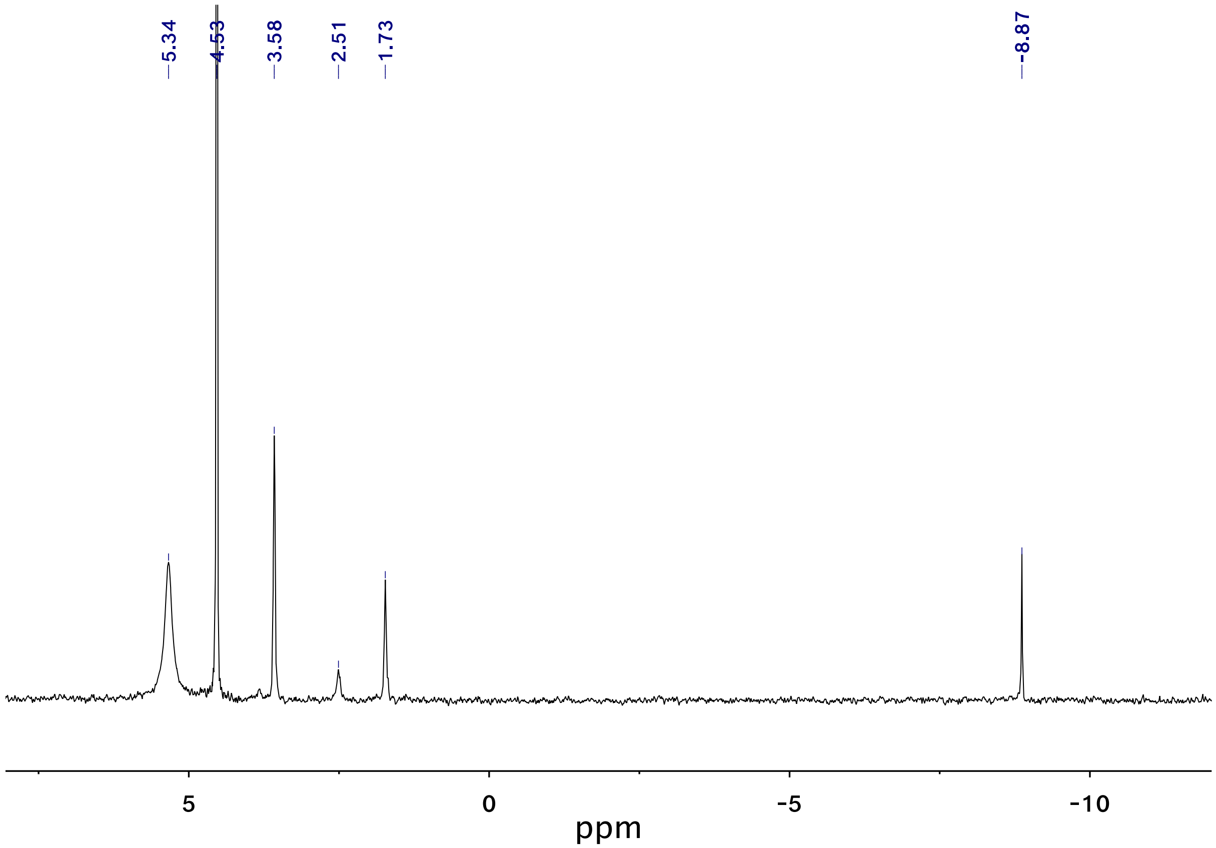

Figure S8. ^{2}H NMR (92 MHz) spectrum in THF from the reaction of **1**, 2 equiv of $NEt_4[MeO^tBu_2ArO]$, and D_2 (7 atm). Deuterium incorporation in to free ligand Anth•$C^{H3}NS^{Me}$ observed at 2.51 ppm is associated with decomposition of the acyl unit upon formation $[HFe(CO)_4]NEt_4$.

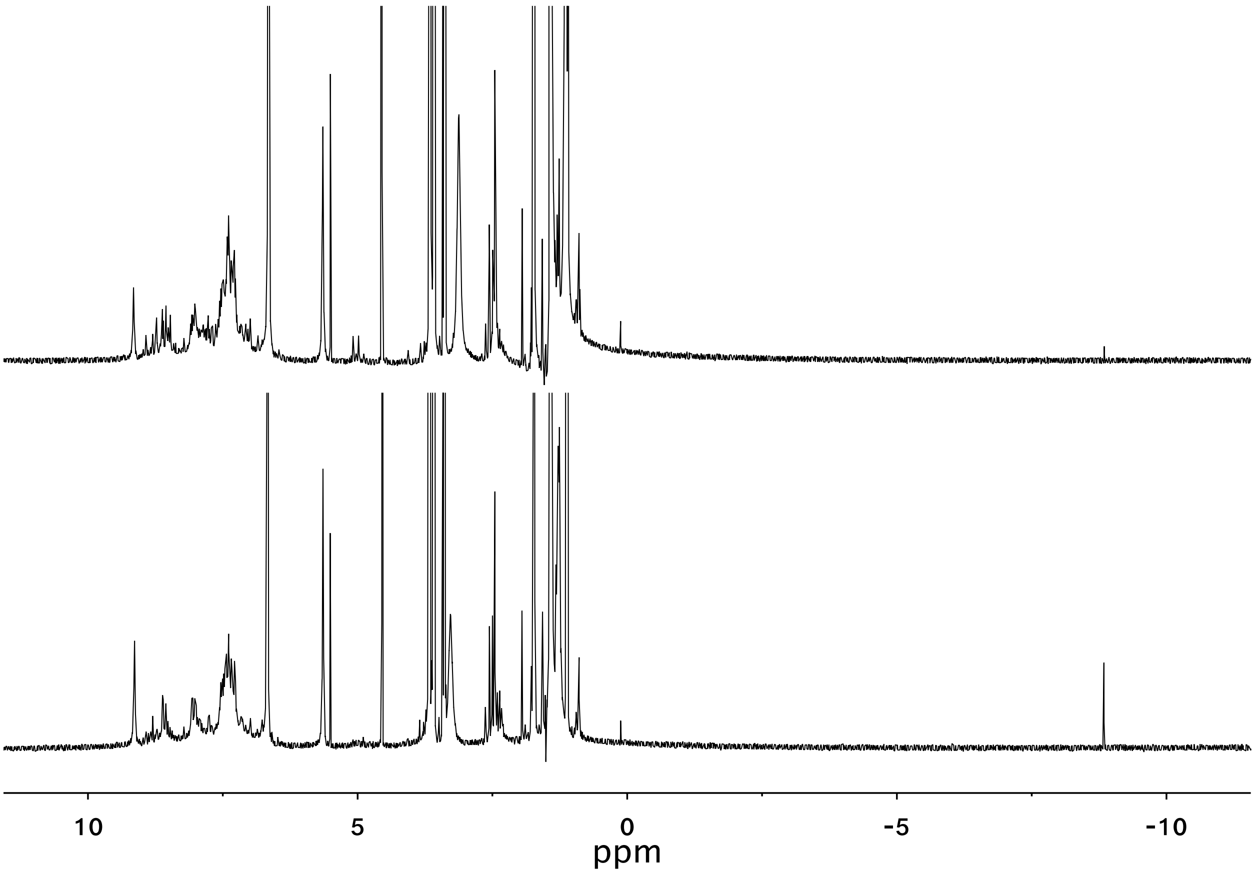

Figure S9. Stacked ^{1}H NMR (400 MHz) spectrum in d^{8}-THF demonstrating generation of $[HFe(CO)_4]NEt_4$ from the reaction of **1**, 2 equiv of $NEt_4[MeO^tBu_2ArO]$, and H_2 (7 atm) in the absence of substrate. The top spectrum is 1 hour after addition of gas and the bottom spectrum is 12 hours after addition of gas.

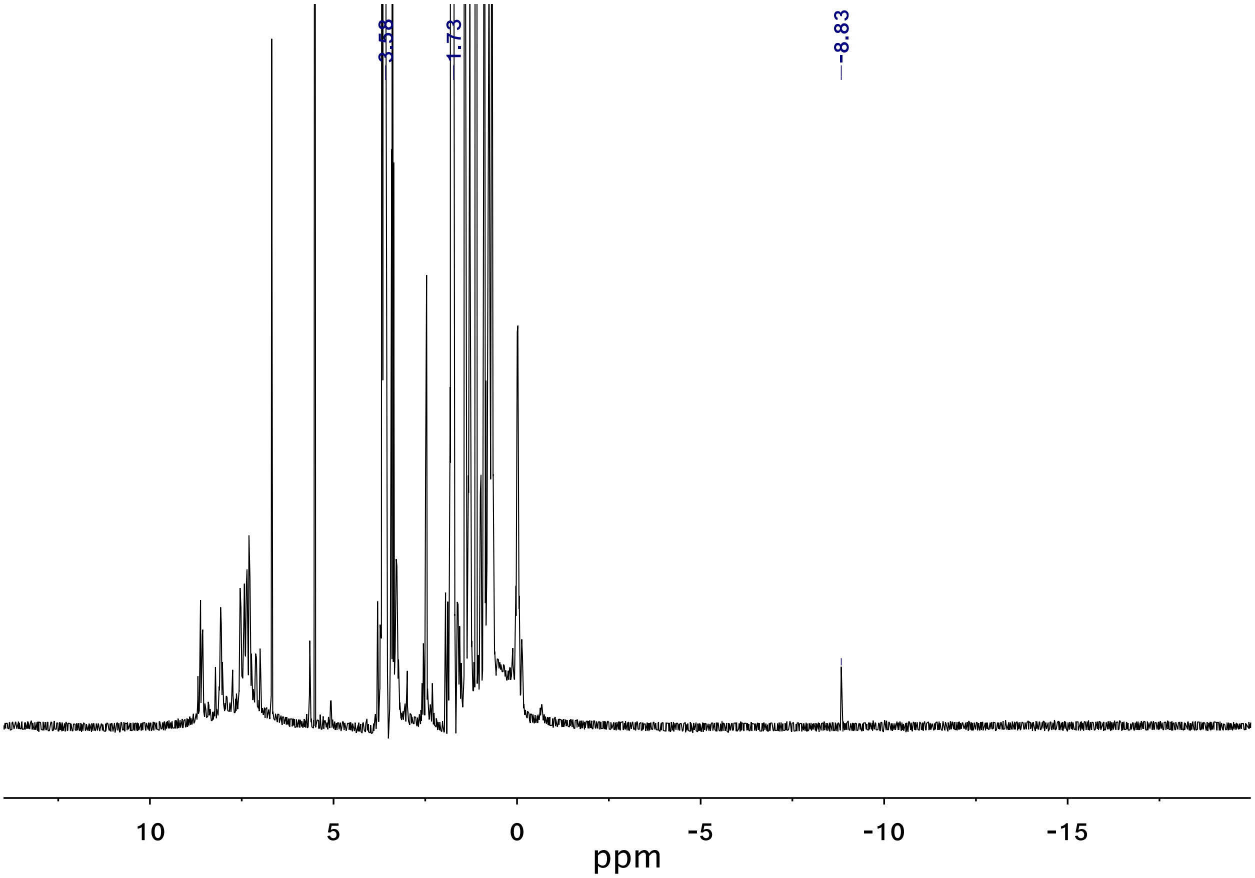

Figure S10. ^{1}H NMR (400 MHz) spectrum in d^8-THF demonstrating generation of $[HFe(CO)_4]NEt_4$ (Fe-H species observed at -8.8 ppm) by treatment of **1** with 1 equiv $NEt_4[MeO^tBu_2ArO]$ and 0.9 equiv $NaHBEt_3$.

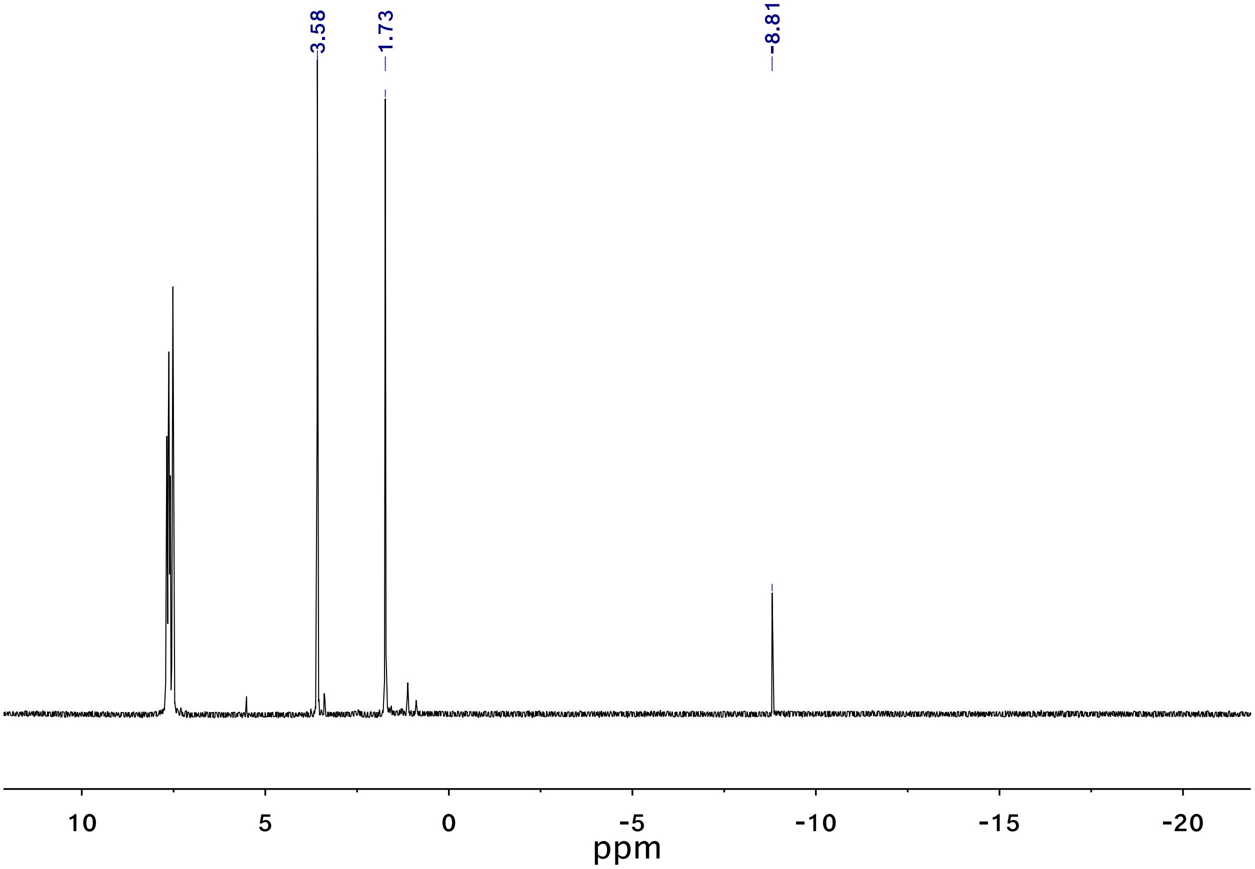

Figure S11. ^{1}H NMR (400 MHz) spectrum in d^8-THF of independently synthesized $[HFe(CO)_4]PPN$.

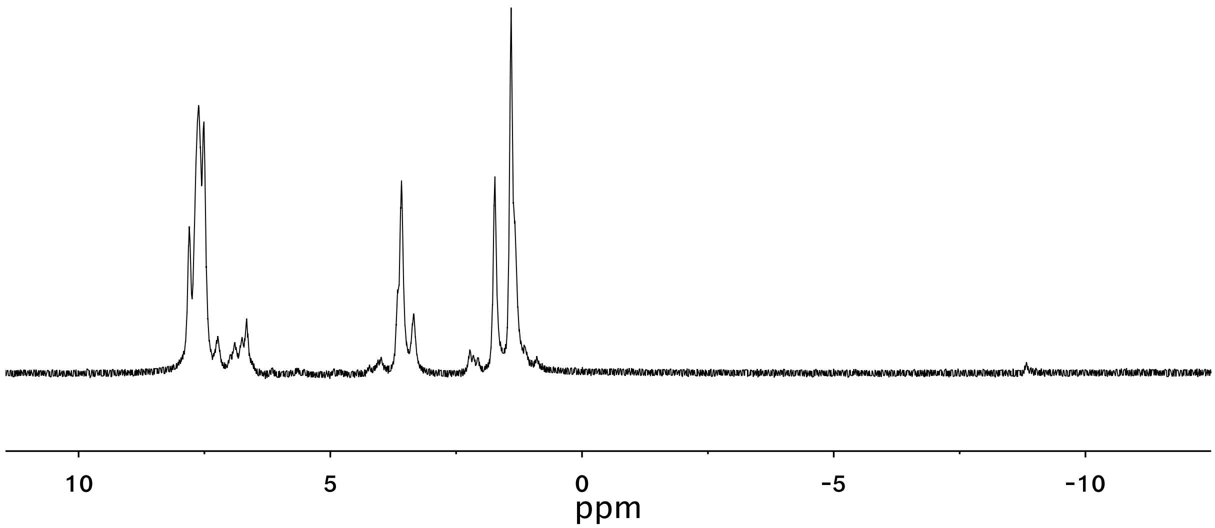

Figure S12. ^{1}H NMR (400 MHz) spectrum in d^8-THF demonstrating line broadening after addition of $NEt_4[MeO^tBu_2ArO]$ to $[HFe(CO)_4]PPN$ consistent with the formation of $(NEt_4)_2[Fe_2(CO)_8]$.

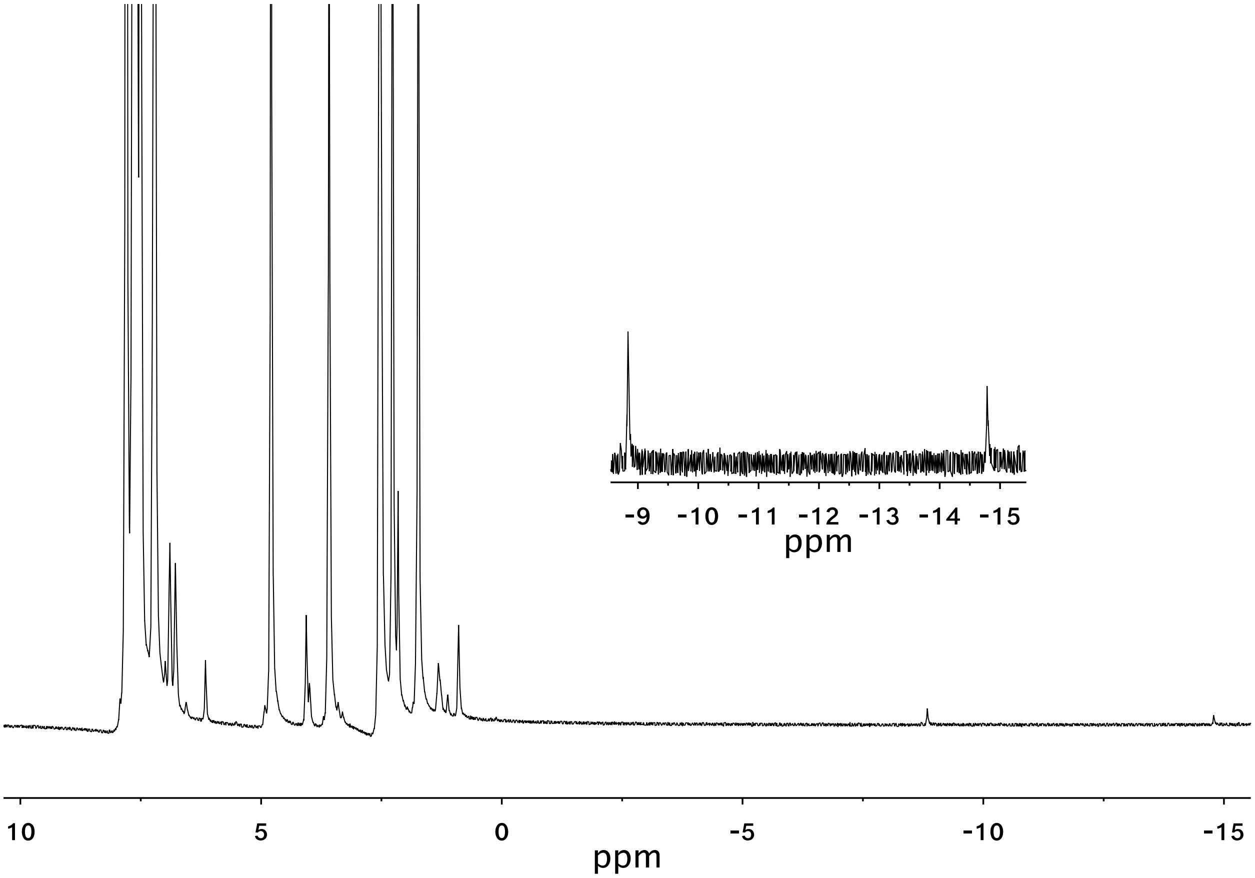

Figure S13. ^1H NMR (400 MHz) spectrum in d^8-THF of the reaction between synthesized $[HFe(CO)_4]PPN$ and $[^{Tol}Im](BAr^F)$ substrate after 24 hours, indicating formation of ^{Tol}ImH (6.14 ppm) and $[HFe_3(CO)_{11}]^-$ (-14.8 ppm).

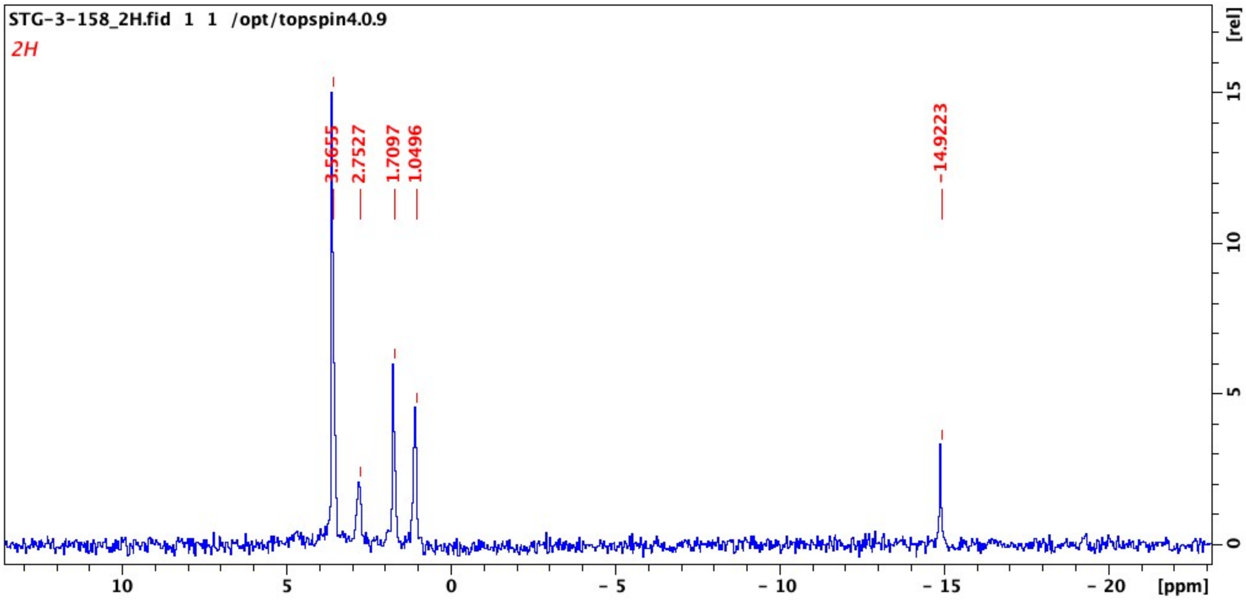

Figure S14. ^{2}H NMR (92 MHz) in THF from the control reaction between $[HFe_3(CO)_{11}]^-$ and $[^{Tol}Im](BAr^F)$ indicating that $[HFe_3(CO)_{11}]^-$ does not perform the hydride transfer reaction.

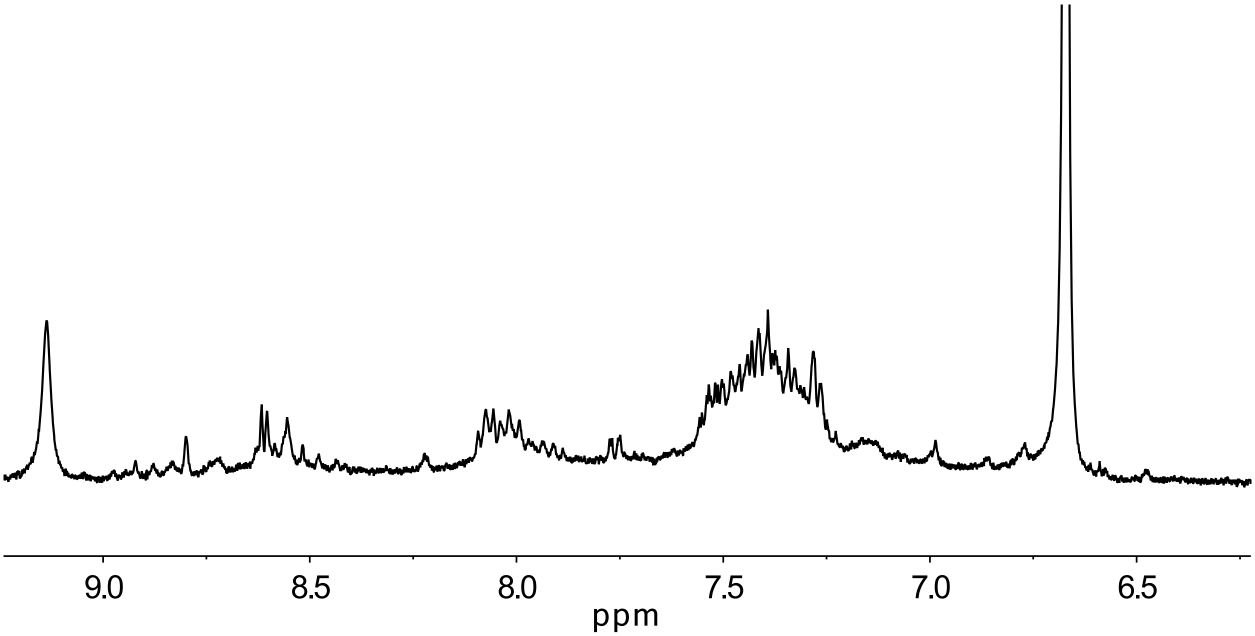

Figure S15. Overlaid ^{1}H NMR (400 MHz) spectrum in d^8-THF from the reaction of **1**, 2 equiv of $NEt_4[MeO^tBu_2ArO]$, and H_2 (7 atm) (black) and the free ligand Anth•$C^{H3}NS^{Me}$ (gray) demonstrating free ligand is generated upon formation $[HFe(CO)_4]NEt_4$.

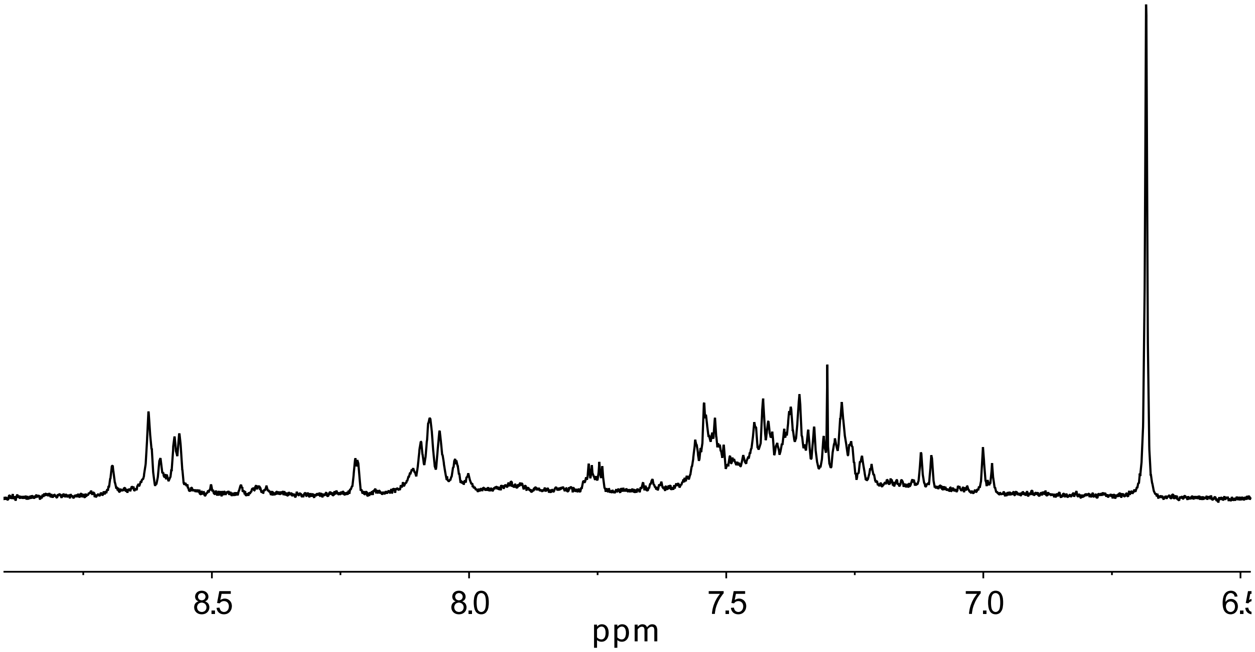

Figure S16. Overlaid ^{1}H NMR (400 MHz) spectrum in d^8-THF from the reaction of **1**, 1 equiv of $NEt_4[MeO^tBu_2ArO]$, and $NaHBEt_3$ (black) and the free ligand Anth•$C^{H3}NS^{Me}$ (gray) demonstrating free ligand is generated upon formation $[HFe(CO)_4]NEt_4$.

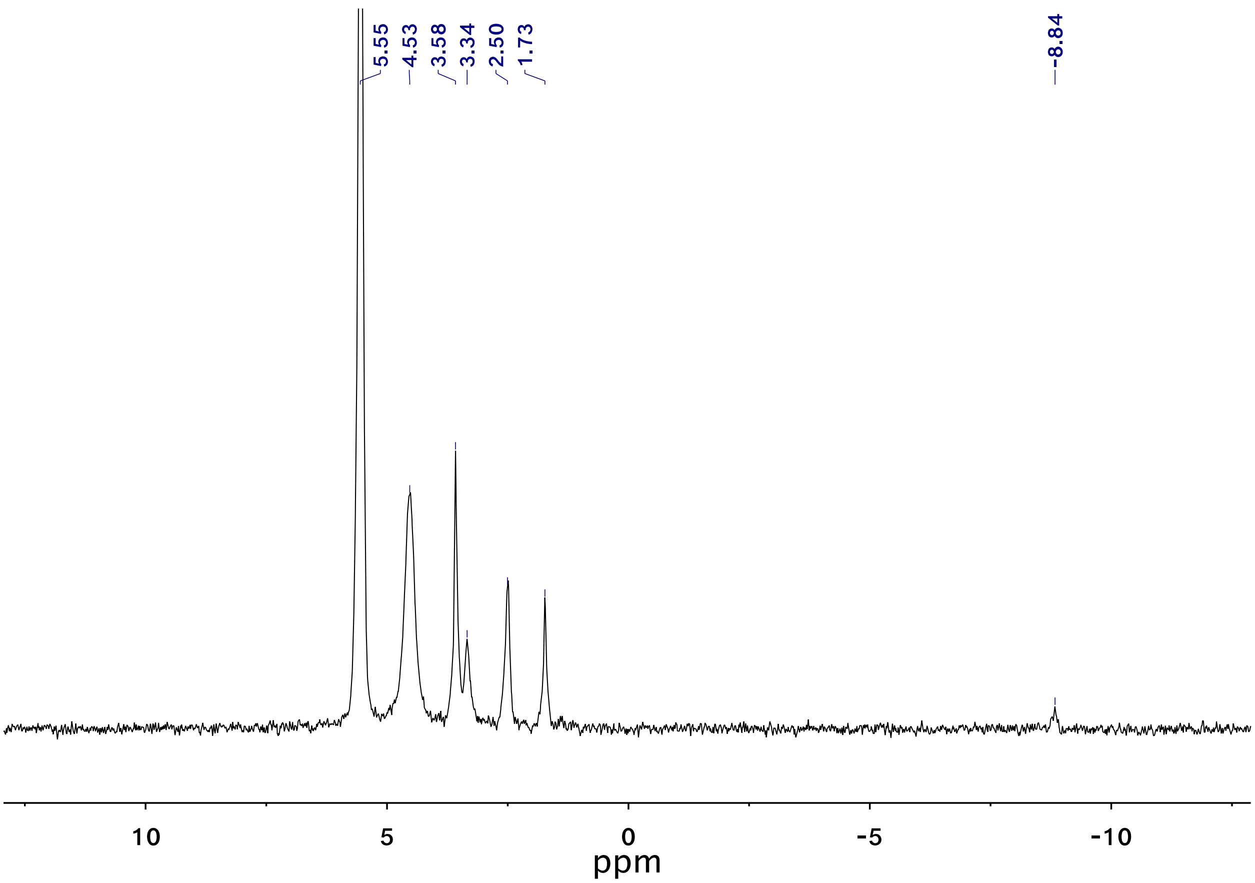

Figure S17. ^{2}H NMR spectrum (92 MHz) in THF of the reaction between independently synthesized Li[(Anth·$C^{H2}N^{off}S^{off}$)$Fe^{0}(CO)_4$] and MeO^{t}Bu$_2$ArOD.

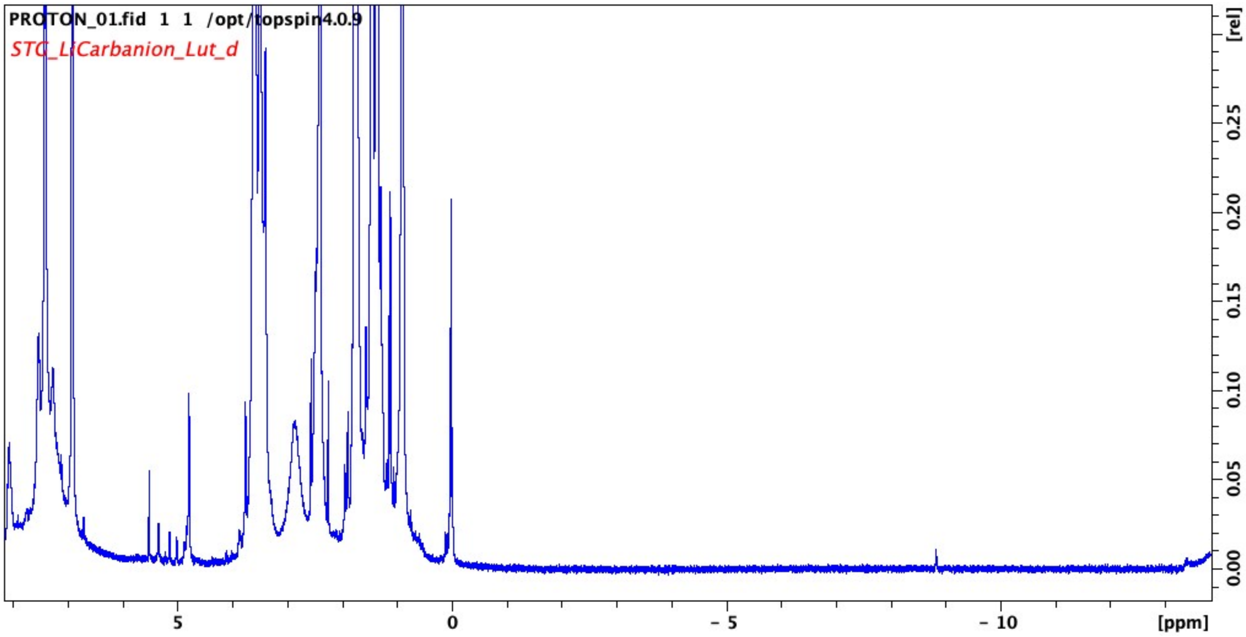

Figure S18. ^{1}H NMR spectrum (400 MHz) in d^{8}-THF of the reaction between independently synthesized Li[(Anth·$C^{H2}N^{off}S^{off}$)$Fe^{0}(CO)_4$] and Lut•HCl indicating formation of $[HFe(CO)_4]^{-}$.

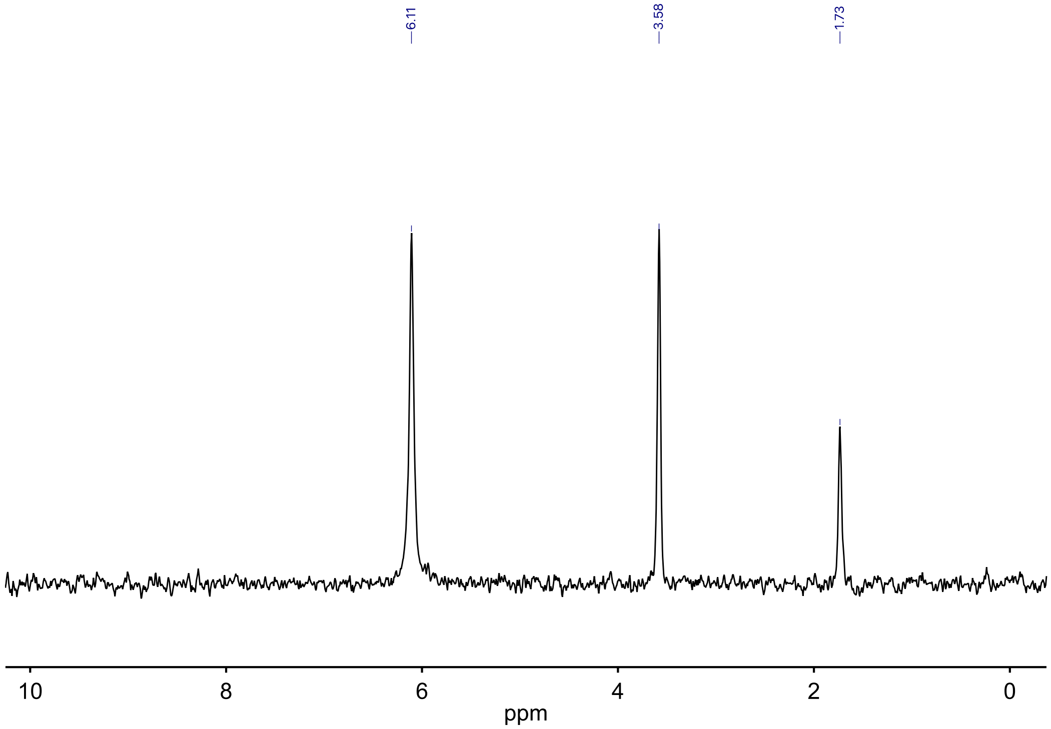

Figure S19. ^{2}H NMR (92 MHz) spectrum of ^{Tol}ImD synthesized from the reaction between [^{Tol}Im](BAr^{F}) and $NaBD_4$ as a control experiment. The procedure was previously reported in reference 3. ^{Tol}Im*D* is observed at 6.11 ppm and THF at 1.73, 3.58 ppm.

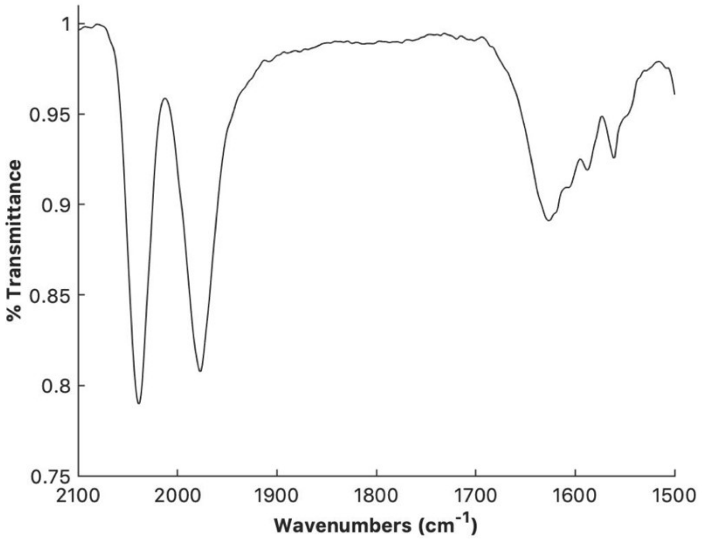

Figure S20. Solid-state (powder) IR spectrum of [(Anth•C^{H2}NS^{Me})Fe(CO)$_2$(Br)] (**1**).

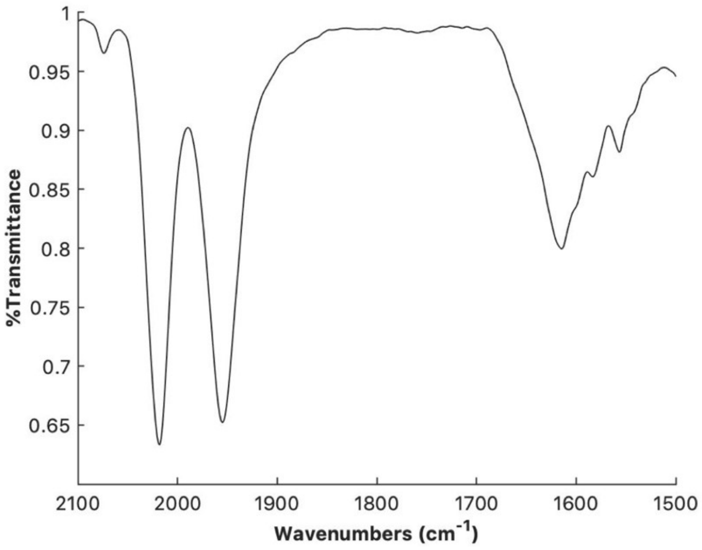

Figure S21. Drop-cast IR spectrum of [(Anth•C^{H2}NS^{Me})Fe(CO)$_2$(Br)] in THF (**1**).

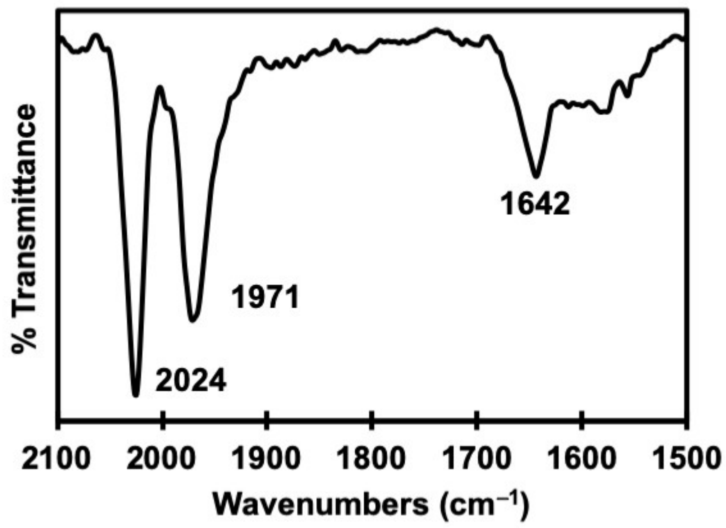

Figure S22. Solid-state IR spectrum of [(Anth•C^{H2}NS^{off})Fe(CO)$_2$(Br)(AsPh$_3$)].

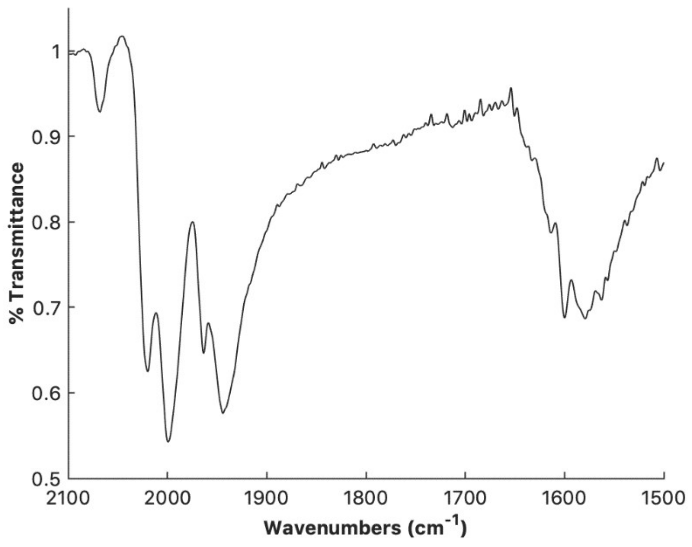

Figure S23. IR spectrum of a crystalline sample of **2**.

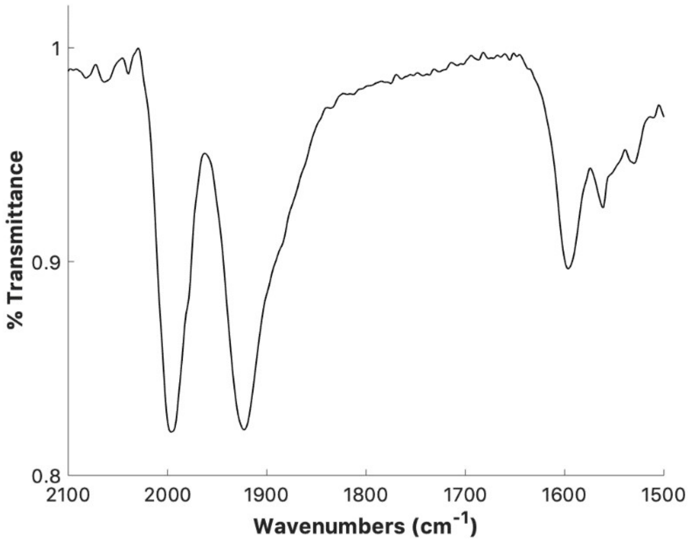

Figure S24. Drop-cast IR spectrum of **1** plus two equiv of NEt_4[MeO^{t}Bu$_2$ArO] in THF.

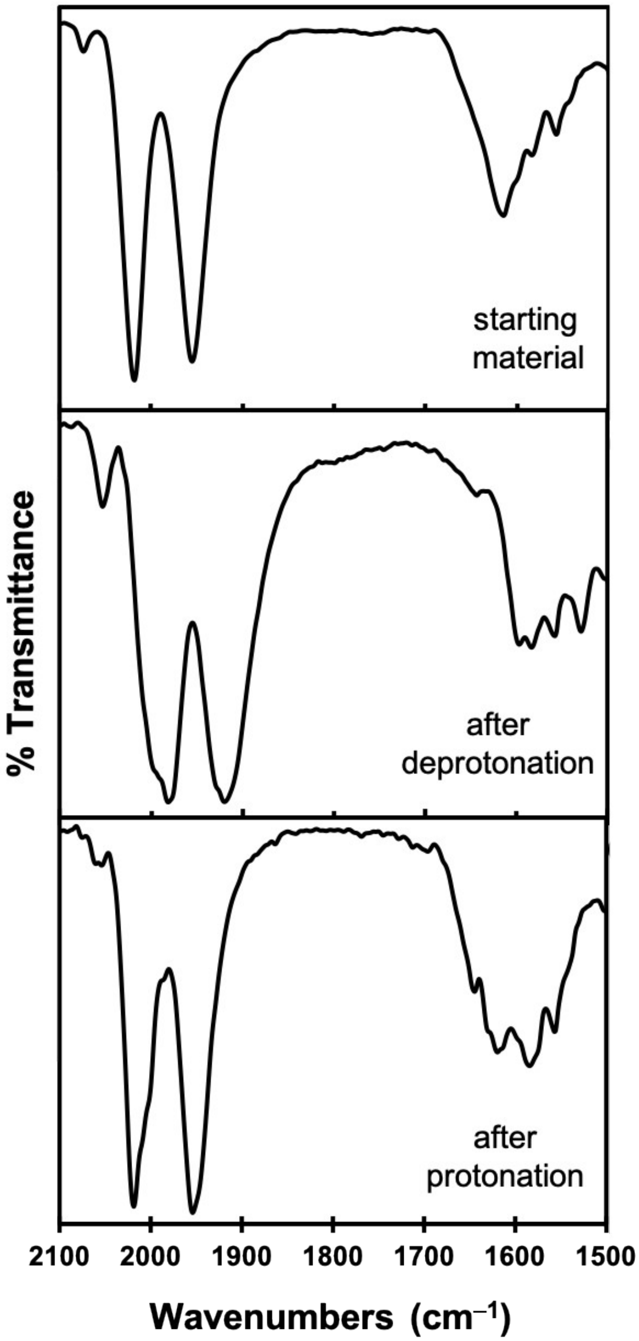

Figure S25. Drop-cast IR spectra in THF demonstrating the deprotonation of **1** by NEt_4[MeO^{t}Bu$_2$ArO] and coordination of one base unit *(middle)* and re-protonation to **1** with Lut•HBr *(bottom)*.

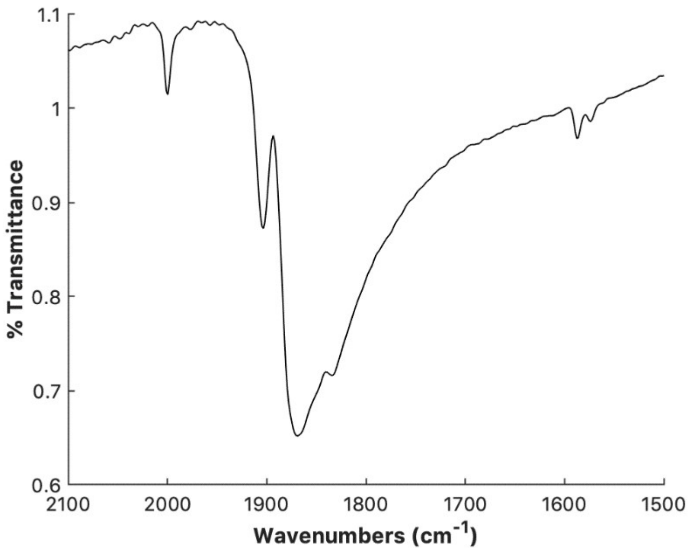

Figure S26. IR spectrum of a crystalline solid sample of independently synthesized $[HFe(CO)_4]PPN$.

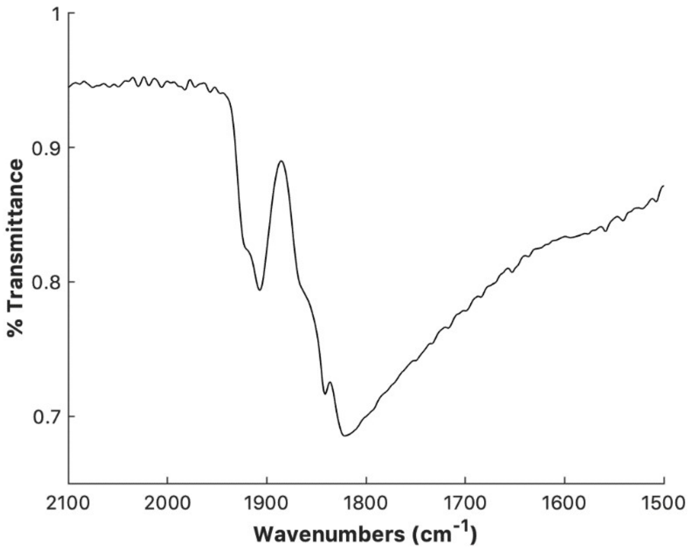

Figure S27. IR spectrum of a crystalline solid sample of $[Fe_2(CO)_8]2NEt_4$ isolated from gas experiment in the absence of model substrate $[^{Tol}Im](BAr^F)$.

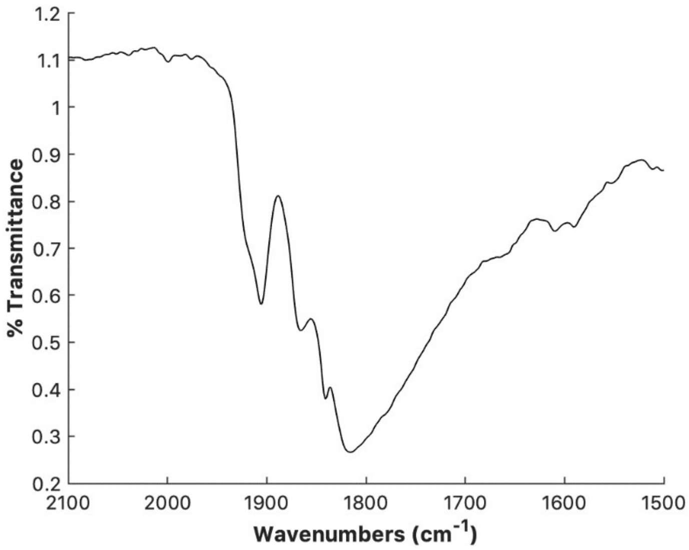

Figure S28. IR spectrum of a powder solid sample of $[Fe_2(CO)_8]2NEt_4$ isolated from the reaction of $[HFe(CO)_4]PPN$ with $NEt_4[MeO^tBu_2ArO]$ base.

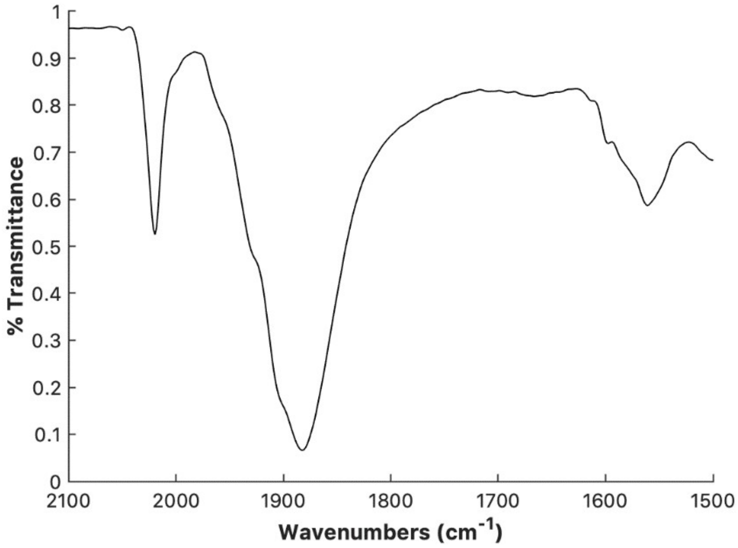

Figure S29. Drop-cast IR spectrum of product resulting from lithiation of Anth•C^{H3}NS^{Me} ligand and addition of $Fe(CO)_5$ in THF to synthesize Li[(Anth·C^{H2}N^{off}S^{off})$Fe^0(CO)_4$].

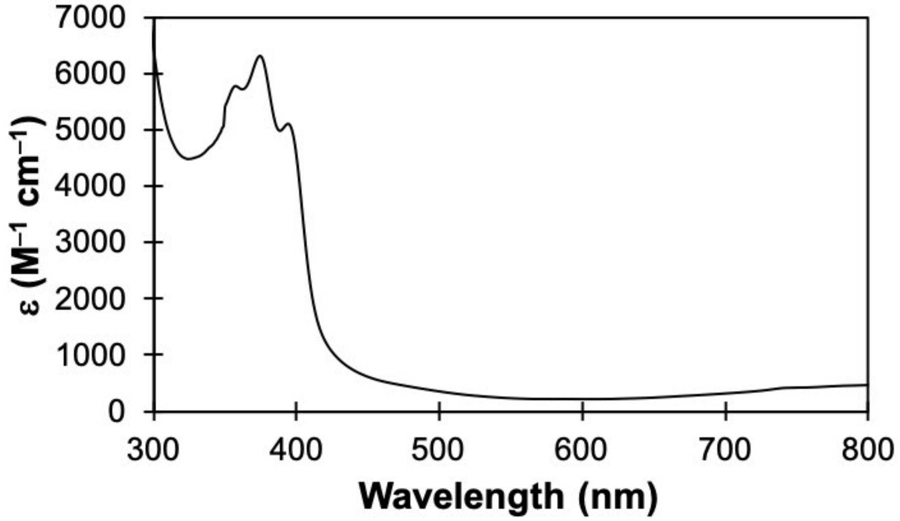

Figure S30: UV-vis spectrum of **1** in THF solvent.

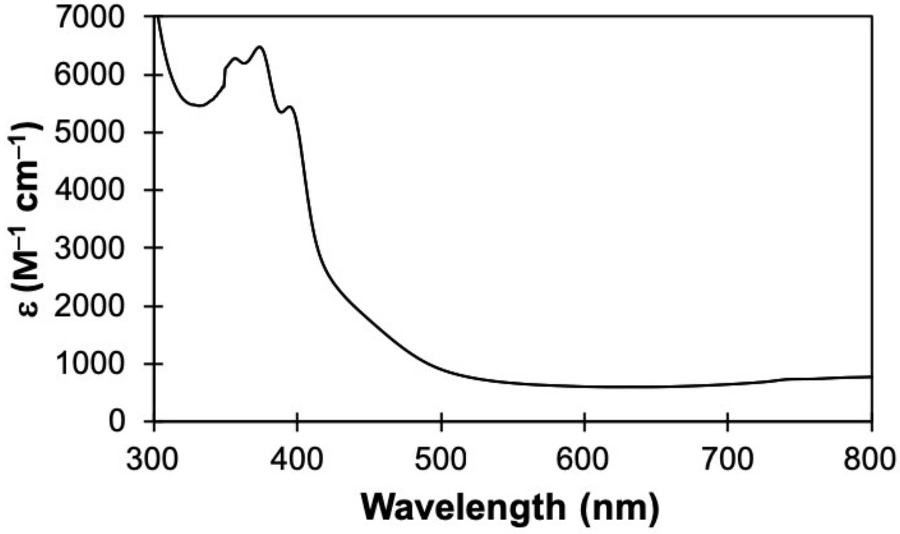

Figure S31: UV-vis spectrum of **2** in THF solvent.

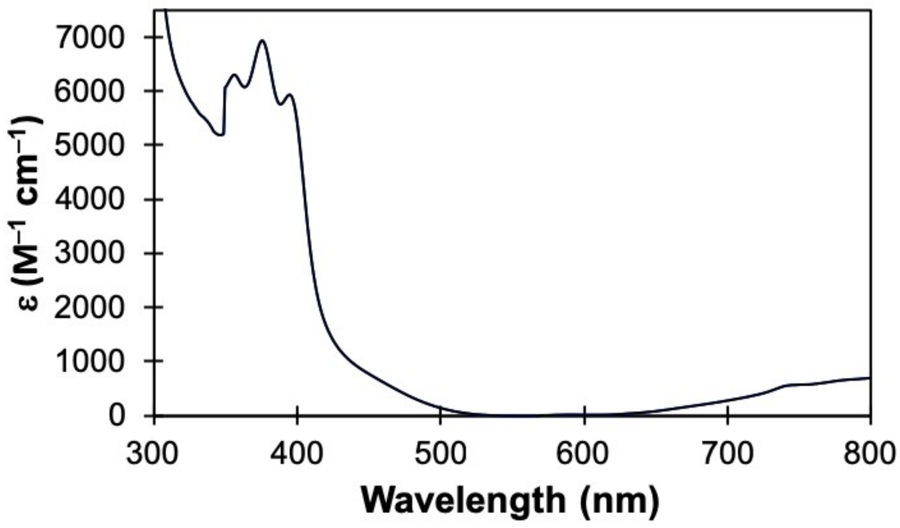

Figure S32: UV-vis spectrum of **1**+2 equiv NEt_4[MeO^tBu_2ArO] in THF solvent.

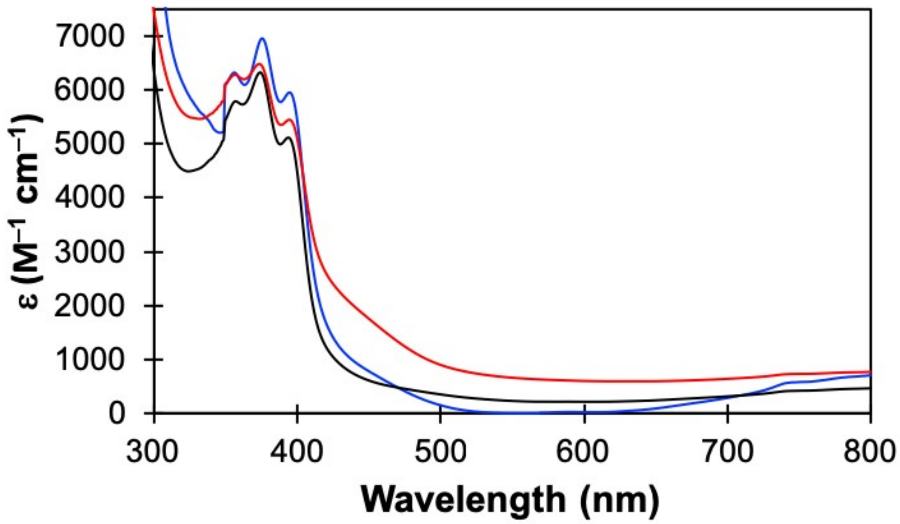

Figure S33: Stacked UV-vis spectra **1** (black), **2** (red), **1**+ 2 equiv NEt_4[MeO^tBu_2ArO] (blue) in THF solvent.

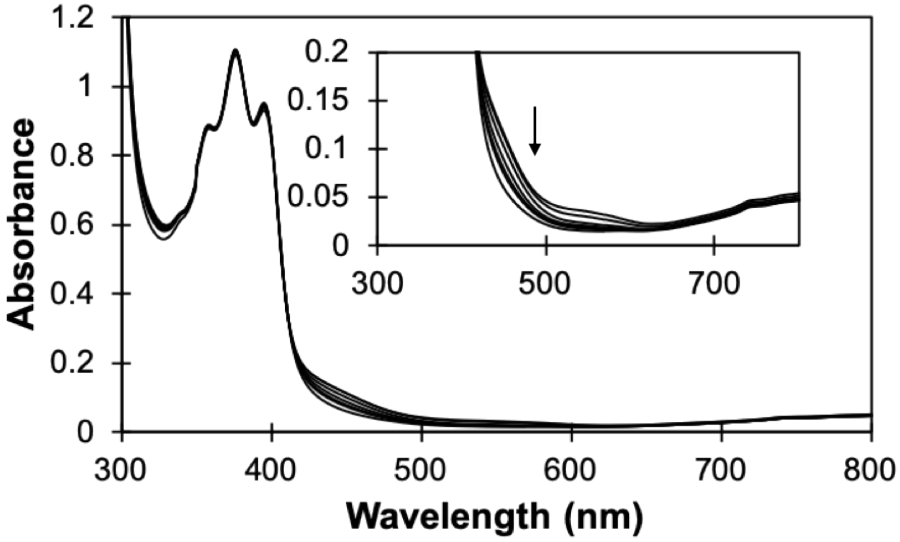

Figure S34: UV-vis spectrum of Li[(Anth·$C^{H2}N^{off}S^{off}$)$Fe^0(CO)_4$] treated with MeO^{t}Bu$_2$ArOH in THF over the course of 20 hours.

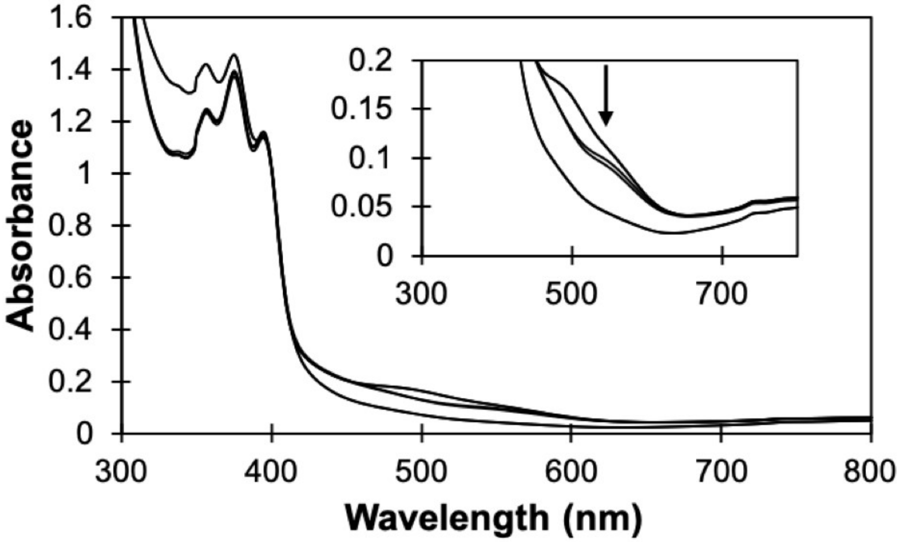

Figure S35: UV-vis spectrum of Li[(Anth·$C^{H2}N^{off}S^{off}$)$Fe^0(CO)_4$] treated with Lut•HCl in THF over the course of 24 hours.

X-ray Photoelectron Spectroscopy (XPS)

XPS was used to detect the presence of the Fe–S bond in complex **1** due to the current unavailability of a single crystal structure of the complex. The binding energy of the S 2*p* electron was compared between species with Fe–S bonding and thioether-S (without Fe–S bond): (*i*) Anth•$C^{H3}NS^{Me}$ ligand, thioether-S without an adjacent Fe ion; (*ii*) an Fe(II) dibromide complex [(Anth•$C^{H3}NS)_2Fe_2(\mu\text{-Br})_2(Br)_2$] (Figure S41), containing an authentic Fe–S bond proven by single crystal X-ray crystallography; (*iii*) [(Anth•$C^{H2}NS^{Me})Fe(CO)_2(Br)$] (**1**) complex.

XP spectra were obtained as using a Kratos Axis Ultra X-ray photoelectron spectrometer with a monochromated Al Kα X-ray source ($h\nu$ = 1486.5 eV). Photoelectron take-off angle was 45° with respect to the X-ray beam, and the analysis chamber pressure was maintained ~2×10^{-9} Torr during the measurement. The obtained spectra were analyzed by the Casa XPS software (version 2.3.15, Casa Software Ltd.). The binding energy of each spectrum was calibrated by adventitious carbon peak at 284.8 eV. The XPS samples were prepared inside a glovebox under N_2 atmosphere. The crushed, microcrystalline samples of the complexes were placed on carbon tape on a sample bar. Next, the bar was placed in a capsule designed for direct connection to XPS instrument without air exposure.

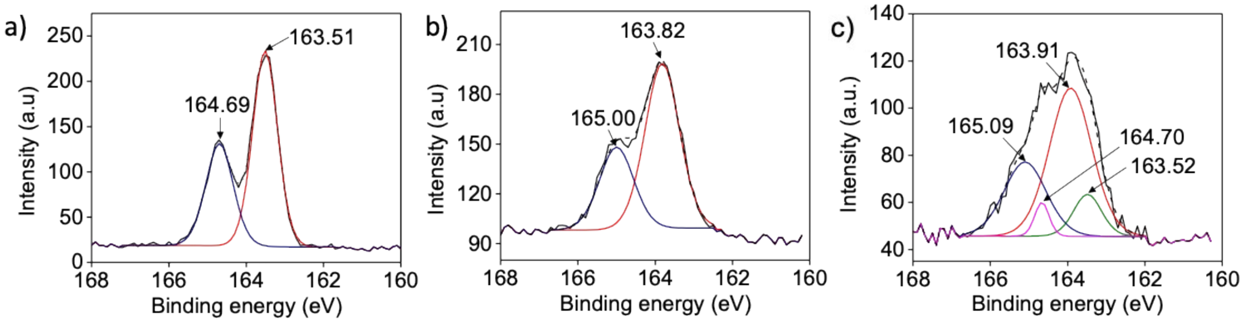

Figure S36. High resolution XP spectra of the S 2*p* region of a) Anth•$C^{H3}NS^{Me}$ (S $2p(^1/_2|^3/_2)$ = 164.69, 163.51 eV) ligand; b) the Fe(II) dibromide complex $[(Anth•C^{H3}NS)_2Fe_2(\mu\text{-}Br)_2(Br)_2]$ (S $2p(^1/_2|^3/_2)$ = 165.00, 163.82 eV); c) mixture (% area ratio = 95:5) of complex **1** (S $2p(^1/_2|^3/_2)$ = 165.09, 163.91 eV) and ligand (S $2p(^1/_2|^3/_2)$ = 164.70, 163.52 eV). (The co-detection of ligand (5%) is possibly due to decomposition of complex during the sample handling for the XPS analysis.)

The independently synthesized and crystallized iron(II) dibromide complex of the ligand, namely $[(Anth•C^{H3}NS^{Me})_2Fe_2(\mu\text{-}Br)_2(Br)_2]$ (X-ray: Fe–S = 2.556(2) Å; X-ray structure, Figure S41), exhibits a +0.4 eV higher energy S 2*p* feature (165.00, 163.82 eV) compared to the free ligand, thus corroborating the S-bound state in **1** that exhibits an S 2*p* feature at 165.09, 163.91 eV.

X-Ray Diffraction Data Collection and Structural Refinement

[(Anth•C^{H2}NS^{off})Fe(CO)$_2$(Br)(AsPh$_3$)]. Crystals grew as thin, orange prisms by slow diffusion of pentane into a FPh solution of the complex at −20 °C. The data crystal had approximate dimensions; 0.236 × 0.128 × 0.076 mm. The dataset was collected on an Agilent Technologies SuperNova Dual Source diffractometer using a μ-focus Cu Kα radiation source (λ = 1.5418 Å) with collimating mirror monochromators. The data were collected at 100 K using an Oxford Cryostream low temperature device. Details of crystal data, data collection and structure refinement are listed in Table S1. Data collection, unit cell refinement and data reduction were performed using Agilent Technologies CrysAlisPro V 1.171.37.31.[9] The structure was solved by direct methods using Superflip[10] and refined by full-matrix least-squares on F^2 with anisotropic displacement parameters for the non-H atoms using SHELXL-2016/6.[11] Structure analysis was aided by use of the programs PLATON98,[12] WinGX[13] and OLEX2.[14] The hydrogen atoms were calculated in ideal positions with isotropic displacement parameters set to 1.2 × U_{eq} of the attached atom (1.5 × U_{eq} for methyl hydrogen atoms).

The function $\Sigma w(|F_o|^2 - |F_c|^2)^2$ was minimized $w = 1/[(\Sigma^2(F_o^2))+(0.0723*P)^2+1.6636*P]$, where $P = (|F_o|^2+2|F_c|^2)/3$. $Rw(F^2)$ refined to 0.1115, with R(F) equal to 0.0417 and a goodness of fit, S, = 1.032. Definitions used for calculating R(F), $Rw(F^2)$ and the goodness of fit, S, are given below.[15] The data were checked for secondary extinction effects, but no correction was necessary. Neutral atom scattering factors and values used to calculate the linear absorption coefficient are from the International Tables for X-ray Crystallography (1992).[16] All figures were generated using SHELXTL/PC.[17] A fluorobenzene solvent molecule is disordered around a crystallographic inversion center at fractional coordinates ½, ½, 1.

Table S1. Crystallographic data and refinement parameters for [(Anth•$C^{H2}NS^{off}$)$Fe(CO)_2(Br)(AsPh_3)$]:

Empirical formula	$C_{51}H_{37.50}AsBrFeNO_3F_{0.50}S_2$	
Formula weight	964.56	
Temperature	99.97(11) K	
Wavelength	1.54184 Å	
Crystal system	Triclinic	
Space group	*P*-1	
Unit cell dimensions	*a = 9.5758(2) Å*	α = 92.631(2)°
	b = 11.0583(2) Å	β = *94.094(2)°*
	c = 19.9278(3) Å	γ = *91.383(2)°*
Volume	2101.80(7) Å	
Z	2	
Density (calculated)	1.524 g/cm^3	
Absorption coefficient	5.710 mm^{-1}	
F(000)	978.0	
Crystal size	0.236 x 0.128 x 0.076 mm^3	
Theta range for data collection	2.225 to 75.377°	
Index ranges	-12 < =h < =12, -13 < =k < =13, -24 < =l < =24	
Completeness to theta = 75.377°	99.0%	
Data / restraints / parameters	8599 / 22 / 569	
Goodness-of-fit on F^2	1.033	
Final R indices [I > 2sigma(I)]	R_1 = 0.0417, wR_2 = 0.1115	
R indices (all data)	R_1 = 0.0448, wR_2 = 0.1151	

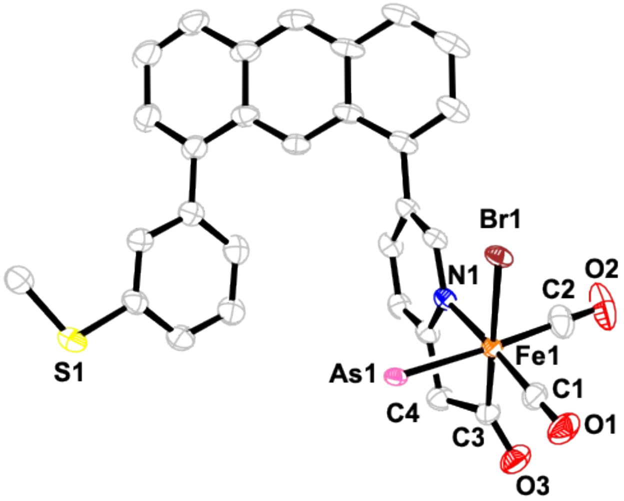

Figure S37. Molecular structure (50% thermal ellipsoids) for $[(Anth{\bullet}C^{H2}NS^{off})Fe(CO)_2(Br)(AsPh_3)]$ complex; hydrogen atoms and fluorobenzene solvent are omitted and the phenyl groups of $AsPh_3$ ligands are truncated for clarity. Selected bond distances (Å): Fe1–C1 = 1.776(5), Fe1–C2 = 1.793(5), Fe1–C3 = 1.942(4), Fe1–N1 = 2.036(3).

Complex 2. Crystals grew as clusters of yellow prisms from an MeCN solution of the complex stored at −20 °C. The data crystal had approximate dimensions; 0.222 × 0.111 × 0.051 mm. The dataset was collected on an Agilent Technologies SuperNova Dual Source diffractometer using a μ-focus Cu Kα radiation source (λ = 1.5418 Å) with collimating mirror monochromators. A total of 1854 frames of data were collected using ω-scans with a scan range of 1° and a counting time of 10 seconds per frame for frames collected with a detector offset of +/- 39.8° and 12.5 seconds per frame with frames collected with a detector offset of 108.6°. The data were collected at 100 K using an Oxford Cryostream low temperature device. Details of crystal data, data collection and structure refinement are listed in Table S2. Data collection, unit cell refinement and data reduction were performed using Rigaku Oxford Diffraction's CrysAlisPro V 1.171.40.39a.[9] The structure was solved by direct methods using SHELXT[18] and refined by full-matrix least-squares on F^2 with anisotropic displacement parameters for the non-H atoms using SHELXL-2016/6.[11] Structure analysis was aided by use of the programs PLATON98,[12] WinGX[13] and OLEX2.[14] The hydrogen atoms were calculated in ideal positions with isotropic displacement parameters set to 1.2 × U_{eq} of the attached atom (1.5 × U_{eq} for methyl hydrogen atoms).

There are two areas of disorder. In one, a methyl-thiophenyl group is disordered by rotation about the C-C bond connecting that group to an anthracene ring. In the second region, a molecule of acetonitrile is disordered. Both disordered groups were modeled using utility programs available in OLEX2. In the region of the disordered methyl-thiophenyl groups, three peaks persisted in the ΔF map near the S atoms. These atoms were assumed to be due to some water molecules. It was also assumed that the three peaks summed to a full water molecule. The SOF of these atoms were constrained to sum to 1 using the SUMP instruction. Their isotropic displacement parameters were constrained to be equal. Hydrogen atoms bound to these water molecules could not be located in a ΔF map and, therefore, were not included in the final refinement model.

The function $\Sigma w(|F_o|^2 - |F_c|^2)^2$ was minimized $w = 1/[(\Sigma^2(F_o^2))+(0.0906*P)^2+8.3422*P]$, where $P = (|F_o|^2+2|F_c|^2)/3$. $R_w(F^2)$ refined to 0.239, with R(F) equal to 0.0904 and a goodness of fit, S, = 1.03. Definitions used for calculating R(F), $R_w(F^2)$ and the goodness of fit, S, are given below.[15] The data were checked for secondary extinction effects, but no correction was necessary. Neutral atom scattering factors and values used to calculate the linear absorption coefficient are from the International Tables for X-ray Crystallography (1992).[16] All figures were generated using SHELXTL/PC.[17] Tables of positional and thermal parameters, bond lengths and angles, torsion angles and figures are found elsewhere.

Table S2. Crystallographic data and refinement parameters for complex **2**.

Empirical formula	$C_{68}H_{50}Fe_2N_6O_7S_2$
Formula weight	1238.96
Temperature	100.1(6) K
Wavelength	1.54184 Å
Crystal system	Triclinic
Space group	*P*-1
Unit cell dimensions	*a = 12.1400(12) Å* α = 85.196(8)°
	b = 14.7031(14) Å β = *78.200(8)°*
	c = 18.3340(17) Å γ = *68.001(9)°*
Volume	2970.0(5) $Å^3$
Z	2
Density (calculated)	1.385 Mg/m^3
Absorption coefficient	5.067 mm^{-1}
F(000)	1280.0
Crystal size	0.222 x 0.111 x 0.051 mm^3
Theta range for data collection	2.462 to 74.154°
Index ranges	-15 < =h < =15, -18 < =k < =17, -22 < =l < =22
Completeness to theta = 67.684°	97.5%
Data / restraints / parameters	11382 / 152 / 871
Goodness-of-fit on F^2	1.036
Final R indices [I > 2sigma(I)]	R_1 = 0.0904, wR_2 = 0.2078
R indices (all data)	R_1 = 0.1415, wR_2 = 0.2386

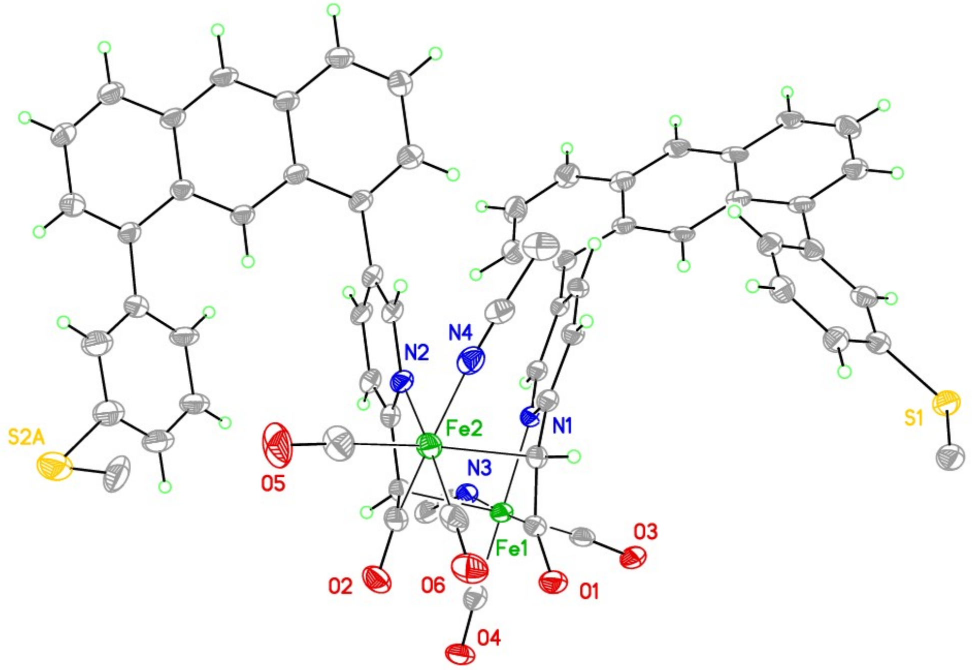

Figure S38. Molecular structure (30% thermal ellipsoids) for **2**.

[(Anth•$C^{H3}NS^{Me})_2Fe_2(\mu$-Br$)_2$(Br$)_2$]. Crystals grew as thin, yellow blocks from a DCM solution of the complex layered with Et_2O stored at room temperature. The data crystal had approximate dimensions; 0.236 × 0.111 × 0.103 mm. The dataset was collected on a Nonius-Kappa CCD diffractometer using a Bruker AXS Apex II detector and a graphite monochromator with Mo Kα radiation (λ = 0.71073 Å). The data were collected at 100 K using an Oxford Cryosystems 700 low-temperature device. Details of crystal data, data collection and structure refinement are listed in Table S3. Data reduction were performed using SAINT V8.27B.[19] The structure was solved by direct methods using Superflip[10] and refined by full-matrix least-squares on F^2 with anisotropic displacement parameters for the non-H atoms using SHELXL-2016/6.[11] Structure analysis was aided by use of the programs PLATON98[12] and WinGX[13]. The hydrogen atoms were calculated in ideal positions with isotropic displacement parameters set to 1.2 × U_{eq} of the attached atom (1.5 × U_{eq} for methyl hydrogen atoms).

The function $\Sigma w(|F_o|^2 - |F_c|^2)^2$ was minimized $w = 1/[(\Sigma^2(F_o^2))+(0.0558P)^2+1.7432*P]$, where $P = (|F_o|^2+2|F_c|^2)/3$. $Rw(F^2)$ refined to 0.0945, with R(F) equal to 0.0377 and a goodness of fit, S, = 1.031. Definitions used for calculating R(F), $Rw(F^2)$ and the goodness of fit, S, are given below.[15] The data were checked for secondary extinction effects, but no correction was necessary. Neutral atom scattering factors and values used to calculate the linear absorption coefficient are from the International Tables for X-ray Crystallography (1992).[16] All figures were generated using SHELXTL/PC.[17]

Table S3. Crystallographic data and refinement parameters for complex [(Anth•$C^{H3}NS^{Me}$)$_2$Fe$_2$(μ-Br)$_2$(Br)$_2$].

Empirical formula	$C_{56}H_{46}Br_4Cl_4Fe_2N_2S_2$	
Formula weight	1384.21	
Temperature	100(2) K	
Wavelength	0.71073 Å	
Crystal system	Triclinic	
Space group	*P*-1	
Unit cell dimensions	*a = 8.769(3) Å*	α = 95.038(6)°
	b = 11.132(3) Å	β = 102.*343(7)°*
	c = 15.368(5) Å	γ = *110.942(6)°*
Volume	1346.3(7) Å	
Z	1	
Density (calculated)	1.707 g/cm^3	
Absorption coefficient	3.823 mm^{-1}	
F(000)	688.0	
Crystal size	0.222 x 0.111 x 0.051 mm^3	
Theta range for data collection	2.768 to 30.564°	
Index ranges	-12 < =h < =12, -15 < =k < =15, -21 < =l < =21	
Completeness to theta = 30.564°	88.7%	
Data / restraints / parameters	7326 / 210 / 318	
Goodness-of-fit on F^2	1.031	
Final R indices [I > 2sigma(I)]	R_1 = 0.0377, wR_2 = 0.0945	
R indices (all data)	R_1 = 0.0565, wR_2 = 0.1037	

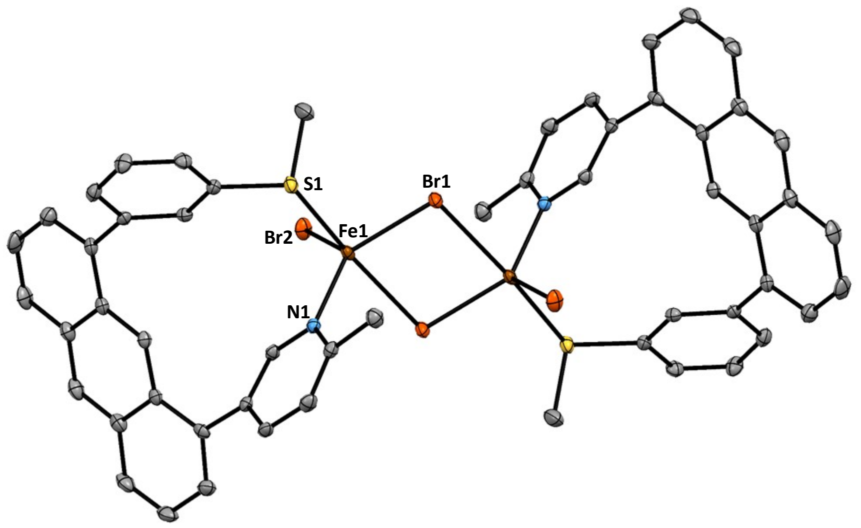

Figure S39. Molecular structure (50% thermal ellipsoids) of the Fe(II) dibromide complex [(Anth•$C^{H3}NS^{Me}$)$_2$Fe$_2$(μ-Br)$_2$(Br)$_2$], determined by X-ray crystallography. Hydrogen atoms and solvent molecules are omitted for clarity. Selected bond distances (Å): Fe1–S1 = 2.5569(11); Fe1–N1 = 2.121(2).

Iron K-edge X-ray Absorption Spectroscopy. Finely ground powders of crystalline **2** or **1** treated with two equiv of $NEt_4[MeO^tBu_2ArO]$ (washed with pentane to remove generated MeO^tBu_2ArOH) were dispersed in nujol (~6.0 mM final concentration in Fe) and mounted into aluminum sample holders between Kapton tape windows and quickly frozen in liquid nitrogen. All data were recorded at the Canadian Light Source (Saskatoon, Saskatchewan, CA) on beamline 06ID-1 (HXMA) at 20 K with temperatures maintained using an Oxford liquid He cryostat. Light was monochromatized using a Si(220) double crystal monochromator, which was detuned 50% for harmonic rejection, and focused using a Rh mirror. Spectra were obtained in fluorescence mode using a 30 element solid-state Ge detector (Canberra) with a 3 micron Mn filter placed between the sample and detector. Spectra were calibrated against the first inflection point of Fe-foil, which was simultaneously recorded with the iron-complex data (7111.2 eV). Data were obtained in 10 eV steps in the pre-edge region (6911 – 7081 eV, 1 s integration time), 0.3 eV steps in the pre-edge region (7081 – 7131 eV, 2 s integration time), 1.0 eV steps in the edge region (7131 – 7311 eV, 2 s integration time), 2.0 eV steps in the near edge region (7311 – 7500 eV, 3 s integration time), and 0.05 k steps in the far edge region (7500 eV – 17.0 Å^{-1}, 3 s integration time). To avoid sample photodamage the 1 × 1 mm beam spot was moved after every 3 scans with no appreciable photodamage noted over this time period. Total fluorescence counts were maintained under 30 kHz, and a deadtime correction yielded no appreciable change to the data. The reported spectra represent the averaged spectra from 6 individual data sets. Prior to data averaging each spectrum and detector channel was individually inspected for data quality.

All refinements were performed on the unfiltered k^3 EXAFS data. Although data were recorded to 17 Å^{-1}, the data were analyzed only to 16.2 Å^{-1} owing to noise at high k. Data were processed

and analyzed as previously reported using *EXAFS123* and FEFF 9.4.[20] Fe-CO multiple scattering pathways were constructed in an identical manner as those outlined for the construction of Fe-NO pathways in reference S1a, except we used 48 "reference" spectra generated over a Fe-C distance range of 1.68 – 1.95 Å and a Fe-C-O bond angle range of 167 – 180°. Errors to the models are reported as ε^2 values over a data range of k = 2.2 – 16.2 $Å^{-1}$ and R' = 1.0 – 3.75 Å. Reported solutions to the EXAFS use an Fe-N scatterer to simulate the non-CO based light atom inner-sphere scatterers, which are a mixture of C, N and O ligand donors. This was done because the N scatterer represented a good average of the O, N, and C phase and amplitude functions of the unresolvable inner-sphere scattering pathways. Wavelet transforms to the k^3-weighted EXAFS data were performed as previously outlined using in-house routines written for MatLab (MathWorks Inc.) using a Morlet motherwavelet with frequency (η = 9.0) and Gaussian broadening parameters (σ = 1.0) for the motherwavelet adjusted such as to achieve a good compromise between resolution in k and R space.[21]

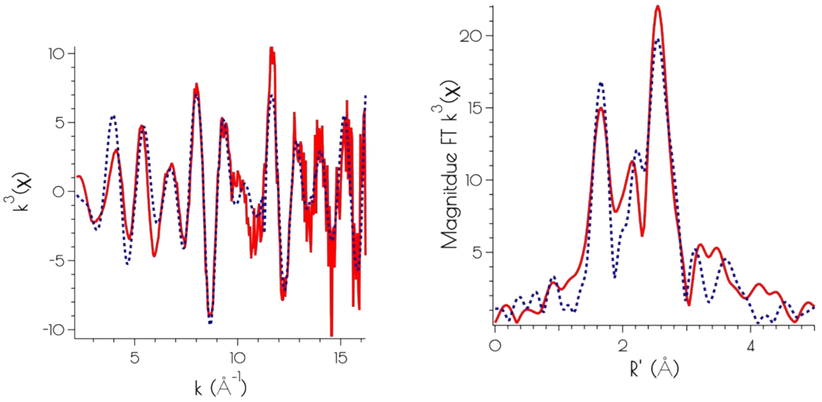

Figure S40. k^3-weighted (left) and magnitude FT k^3-wighted (right) EXAFS data for **2** (experimental as the solid red spectrum and best model as the blue dashed spectrum.

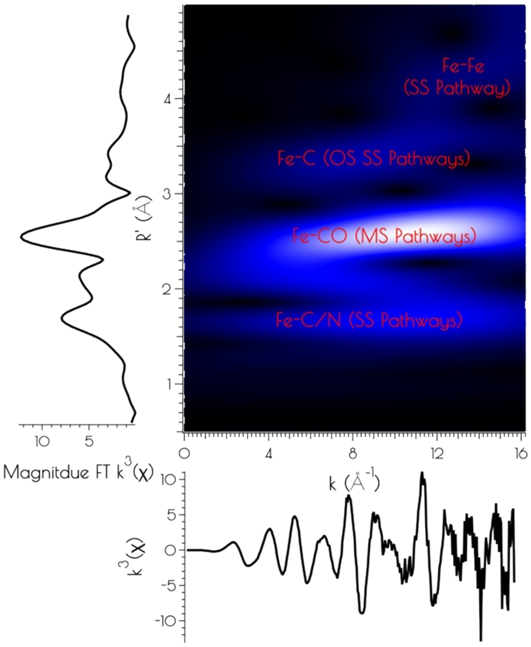

Figure S41. Wavelet transform of **2** with contributions from the various pathways scattering pathways highlighted on the 2D wavelet transform.

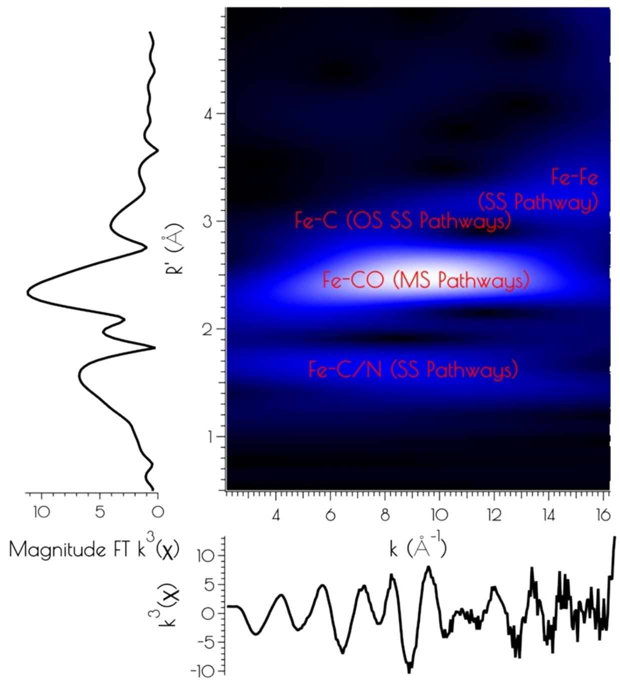

Figure S42. Wavelet transform of **1** treated with two equiv of NEt_4[MeO^{t}Bu$_2$ArO] with contributions from the various pathways scattering pathways highlighted on the 2D wavelet transform.

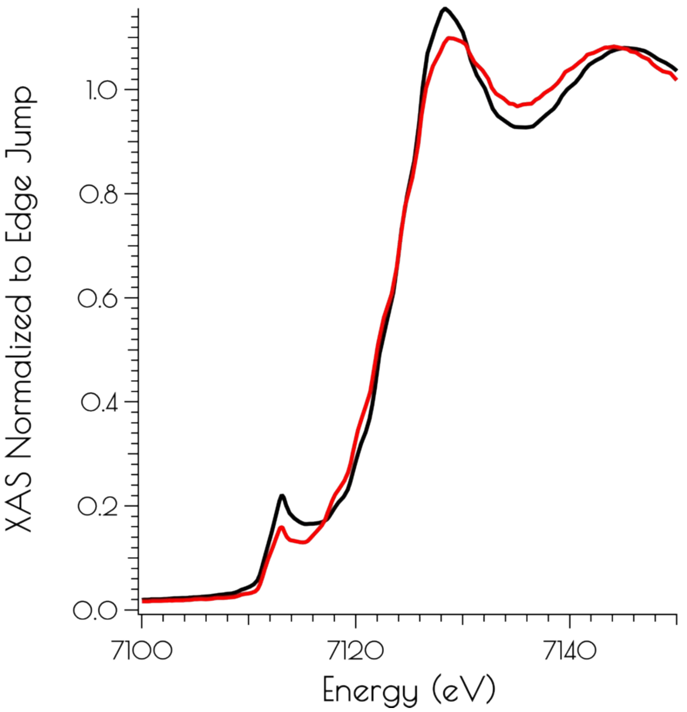

Figure S43. Overlay of the XANES region for **2** (red) and **1** treated with two equiv of $NEt_4[MeO^tBu_2ArO]$ (black).

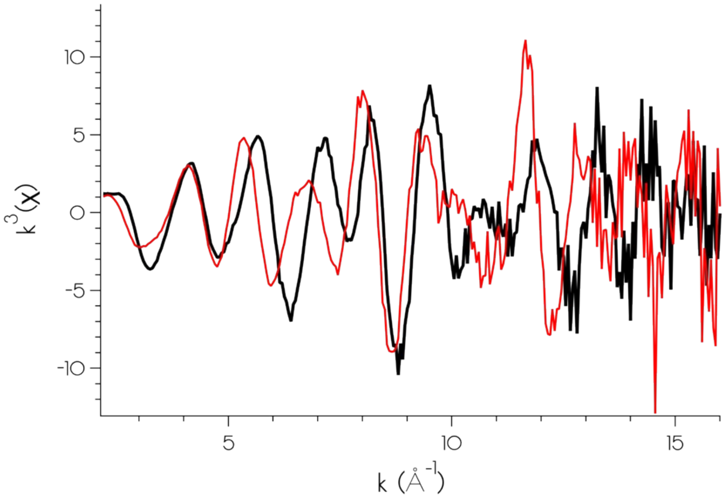

Figure S44. Overlay of k^3-weighted EXAFS data for **2** (red) and **1** treated with two equiv of $NEt_4[MeO^tBu_2ArO]$ (black).

Table S4. Reported and alternative fits to the EXAFS data for **2**. E_o = 7132.7 eV.

	Unrestrained (X = N)	5 coord. (X = N)	6 coord. (X = N)	No Fe Shell (X = N)	No CO MS (X = N)
Fe-CO					
N	1.6(2)	2	2	2	2
R (Å)	1.788(3)	1.781(3)	1.793(5)	1.784(2)	1.816(5)
σ^2(Å^2)	0.0035(9)	0.0037(17)	0.0035(10)	0.0034(2)	0.0016(5)
θ (°)	174.3(6)	175.0(12)	175.1(8)	174.6(7)	N/A
R' Fe•••O (Å)	2.819(5)	2.820(7)	2.820(8)	2.822(5)	N/A
Fe-X					
N	3.4(3)	3	4	3.5	3.5
R (Å)	2.047(5)	2.041(5)	2.051(5)	2.040(6)	2.041(11)
σ^2(Å^2)	0.0013(8)	0.0034(19)	0.0031(5)	0.0027(4)	0.0032(5)
Fe-Fe					
N	1.2(14)	1	1	---	1
R (Å)	3.80(10)	3.81(13)	3.80(11)		3.878(15)
σ^2(Å^2)	0.0057(8)	0.005(2)	0.005(1)		0.001(5)
Fe-C					
N	1.8(4)	1.8(4)	1.8(4)	1.4(4)	6(2)
R (Å)	2.978(4)	2.97(1)	2.980(8)	2.966(3)	2.793(14)
σ^2(Å^2)	0.0024(7)	0.003(1)	0.003(1)	0.0027(5)	0.0011(13)
Fe-C					
N	2.8(2)	2.6(4)	2.8(5)	3.1(4)	5.1(2)
R (Å)	3.51(1)	3.49(1)	3.503(16)	3.61(1)	2.977(2)
σ^2(Å^2)	0.0025(11)	0.002(1)	0.0033(9)	0.001(1)	0.007(3)
ε^2	2.19	2.04	2.15	4.11	5.46

Table S5. Reported and alternative fits to the EXAFS data for **1** treated with two equiv of $NEt_4[MeO^tBu_2ArO]$. E_o = 7131.9 eV.

	Reported (X = N)	Unrestrained (X = N)	Alt Fit #1 (X = O)	Alt Fit #2 (X = C)	No Fe Shell (X = N)	No CO MS (X = N)
Fe-CO						
N	2	1.81(13)	2	2	2	2
R (Å)	1.767(2)	1.766(2)	1.769(2)	1.767(2)	1.765(2)	1.794(10)
σ^2(Å^2)	0.002(1)	0.0016(4)	0.0022(2)	0.0019(2)	0.0020(2)	0.0028(10)
θ (°)	176.8(7)	176.7(2)	176.3(5)	176.5(4)	176.0(9)	N/A
R' Fe•••O (Å)	2.883(3)	2.880(3)	2.885(2)	2.883(2)	2.876(3)	N/A
Fe-X						
N	3	2.6(3)	3	3	3	3
R (Å)	2.028(9)	2.047(4)	2.003(4)	2.091(4)	2.034(5)	2.063(11)
σ^2(Å^2)	0.004(2)	0.0020(8)	0.0048(2)	0.0025(4)	0.0031(4)	0.0049(13)
Fe-Fe						
N	1	0.82(14)	1	1	---	1
R (Å)	3.442(4)	3.441(4)	3.443(3)	3.442(3)		3.448(7)
σ^2(Å^2)	0.002(1)	0.00011(8)	0.002(1)	0.001(1)		0.0004(5)
Fe-C						
N	3.2(4)	1.3(4)	2.8(3)	2.4(4)	2.6(5)	0.3(3)
R (Å)	2.54(1)	2.545(6)	2.539(6)	2.540(6)	2.555(7)	2.389(3)
σ^2(Å^2)	0.005(1)	0.0091(10)	0.006(1)	0.0063(10)	0.0011(9)	0.0005(3)
ε^2	1.51	1.88	1.84	1.77	2.75	8.89

References

(1) Hieber, W.; Bader, G. *Chem. Ber.* **1928**, *61*, 1717.
(2) Robertson, E. W.; Wilkin, O. M.; Young, N. A. *Polyhedron* **2000**, *19*, 1493.
(3) Kalz, K. F.; Brinkmeier, A.; Dechert, S.; Mata, R. A.; Meyer, F. *J. Am. Chem. Soc.* **2014**, *136*, 16626.
(4) Cullinane, J.; Jolleys, A.; Mair, F. S. *Dalton Trans.* **2013**, *42*, 11971.
(5) Goichi, M.; Segawa, K.; Suzuki, S.; Toyota, S. *Synthesis* **2005**, *13*, 2116.
(6) Pérez-Trujillo, M.; Maestre, I.; Jaime, C.; Alvarez-Larena, A.; Piniella, J. F.; Virgili, A. *Tetrahedron: Asymmetry* **2005**, *16*, 3084.
(7) M. Y. Darensbourg, D. J. Darensbourg and H. L. C. Barros, *Inorg. Chem.*, 1978, **17**, 297–301.
(8) Shupp, J. P.; Rose, A. R.; Rose M. J.; Synthesis and Interconversion of Reduced, Alkali-Metal Supported Iron-Sulfur-Carbonyl Complexes. *Dalton Trans.* **2017**, *46*, 9163-9171.
(9) CrysAlisPro. Rigaku Oxford Diffraction (2019). CrysAlisPro Software System, 1.171.40.39a.
(10) Superflip. Palatinus, L. and Chapuis, G. *J. Appl. Cryst.* **2007**, *40*, 786-790.
(11) Sheldrick, G. M. SHELXL-2016/6. Program for the Refinement of Crystal Structures. *Acta Cryst.* **2015**, *C71*, 9-18.
(12) Spek, A. L. PLATON, A Multipurpose Crystallographic Tool. Utrecht University, The Netherlands. **1998**.
(13) WinGX 1.64. An Integrated System of Windows Programs for the Solution, Refinement and Analysis of Single Crystal X-ray Diffraction Data. Farrugia, L. J. *J. Appl. Cryst.* **1999**, *32*, 837-838.
(14) OLEX2: a complete structure solution, refinement and analysis program. Dolomanov, O. V., Bourhis, L. J., Gildea, R. J., Howard, J. A. K., Puschmann, H. J. Appl. Cryst. 2009, 42, 339-341.
(15) $R_w(F^2) = \{\Sigma w(|Fo|^2 - |Fc|^2)^2 / \Sigma w(|Fo|)^4\}^{1/2}$ where w is the weight given each reflection.
$R(F) = \Sigma(|Fo| - |Fc|)/\Sigma|Fo|\}$ for reflections with $Fo > 4(\sigma(Fo))$.
$S = [\Sigma w(|Fo|^2 - |Fc|^2)\ 2 /(n - p)]^{1/2}$, where n is the number of reflections and p is the number of refined parameters.
(16) International Tables for X-ray Crystallography 1992, Vol. C, Tables 4.2.6.8 and 6.1.1.4, A. J. C. Wilson, Editor, Boston: Kluwer Academic Press.
(17) Sheldrick, G. M. (1994). SHELXTL/PC (Version 5.03). Siemens Analytical X-ray Instruments, Inc., Madison, Wisconsin, USA
(18) SHELXT. (2015). G. M. Sheldrick. A program for crystal structure solution. Acta Cryst. A71, 3-8.
(19) Saint V8.27 B. SAINT V8.27B Bruker AXS Inc, (2012): Madison, WI.
(20) (a) Scarrow, R. C.;Strickler, B. S.; Ellison, J. J.; Shoner, S. C.; Kovacs, J. A.; Cummings, J. G.; Nelson, M. J. *J. Am. Chem. Soc.*, **1998**, *120*, 9237-9245. b) Brennan, B. A.; Alms, G.; Nelson, M. J., Curney, L. T.; Scarrow, R. C. *J. Am. Chem. Soc.*, **1996**, *118*, 9194-9195. c) Scarrow, R. S.; Shearer, J. EXAFS123, v. 0.5; Trinity University: San Antonio, TX, 2019. (d) Rehr, J.J.; Kas, J.J.; Vila, F.D.; Prange, M.P.; Jorissen, K. *Phys. Chem. Chem. Phys.* **2010**, *12*, 5503.
(21) Penfold, T.J.; Tavernelli, I.; Milne, C.J.; Reinhard, M.; El Nahhas, A.; Abela, R.; Rothlisberger, U; Chergui, M. *J. Chem. Phys.* **2013**, *138*, 014104.
